# Simple and Complex Centromeric Satellites in *Drosophila* Sibling Species

**DOI:** 10.1534/genetics.117.300620

**Published:** 2018-01-05

**Authors:** Paul B. Talbert, Sivakanthan Kasinathan, Steven Henikoff

**Affiliations:** * Basic Sciences Division, Fred Hutchinson Cancer Research Center, Seattle, Washington 98109; † Howard Hughes Medical Institute, Seattle, Washington 98109; ‡ Medical Scientist Training Program, University of Washington School of Medicine, Seattle, Washington 98195

**Keywords:** ChIP, Cid, rotational phasing, prod

## Abstract

Centromeres are the chromosomal sites of assembly for kinetochores, the protein complexes that attach to spindle fibers and mediate separation of chromosomes to daughter cells in mitosis and meiosis. In most multicellular organisms, centromeres comprise a single specific family of tandem repeats—often 100–400 bp in length—found on every chromosome, typically in one location within heterochromatin. *Drosophila melanogaster* is unusual in that the heterochromatin contains many families of mostly short (5–12 bp) tandem repeats, none of which appear to be present at all centromeres, and none of which are found only at centromeres. Although centromere sequences from a minichromosome have been identified and candidate centromere sequences have been proposed, the DNA sequences at native *Drosophila* centromeres remain unknown. Here we use native chromatin immunoprecipitation to identify the centromeric sequences bound by the foundational kinetochore protein cenH3, known in vertebrates as CENP-A. In *D. melanogaster*, these sequences include a few families of 5- and 10-bp repeats; but in closely related *D. simulans*, the centromeres comprise more complex repeats. The results suggest that a recent expansion of short repeats has replaced more complex centromeric repeats in *D. melanogaster*.

THE separation of chromosomes in mitosis and meiosis is orchestrated by the kinetochore, a protein complex usually found at one location on each chromosome, termed the centromere. The kinetochore attaches chromosomes to spindle microtubules and mediates alignment on the metaphase plate, senses tension, and controls entry into anaphase (Musacchio and Desai 2017; Salmon and Bloom 2017). A key protein of the kinetochore is a centromeric variant of histone H3 (cenH3), which forms specialized nucleosomes that wrap centromeric DNA (Steiner and Henikoff 2015). Despite their conserved function, both centromeres and cenH3s evolve rapidly (Henikoff *et al.* 2001; Rosin and Mellone 2017), with little conservation of centromere sequences between closely related species (Lee *et al.* 2005; Gong *et al.* 2012). Despite sequence divergence, in most plants and animals centromeres have a common organization: they are embedded in heterochromatin and typically comprise megabase-scale arrays of tandem repeats (satellite DNAs) that are recalcitrant to genome assembly methods, with repeat monomers often of lengths of ∼100–400 bp (Melters *et al.* 2013). A single repeat family typically dominates the centromeres of all chromosomes in a species, and is partially occupied by cenH3 nucleosomes (Schueler *et al.* 2001; Cheng *et al.* 2002; Zhong *et al.* 2002; Nagaki *et al.* 2003; Henikoff *et al.*, 2015).

This pattern does not apply to *Drosophila melanogaster*. Instead, major satellite repeats are mostly tandem arrays of very short 5- to 12-bp sequences, often following the pattern RRNRN or RRNRNRN, where R is a purine and N is any nucleotide (Lohe and Brutlag 1986). The distribution of these short satellites, which have been mapped by *in situ* hybridization to specific bands in heterochromatin (Lohe *et al.* 1993), appears to preclude the possibility of a single repeat family found at the centromere of every chromosome. Nor does it appear that any satellite sequence is restricted to centromeres. A summary of localization of selected satellites in prior studies is given in Table 1.

**Table 1:** Selected candidate centromeric satellite repeats in *D. melanogaster*

Repeat	Prior localization*^a^*	Localization confirmed by FISH in this report	Anti-Cid ChIP enrichment in this report
AATAT	*X*,*Y*, *4*; centromeric (*X*)*^b^*	X, 4; centromeric (*X*)	Yes
AATAG	*X*, *Y*, *2*, *4*; noncentromeric	2; noncentromeric	Yes
AAGAG	*X*, *Y*, *2*, *3*, *4*; centromeric (X)*^c^*	Noncentromeric (*X*)	No
*Prodsat*: AATAACATAG	*2*, *3*; pericentromeric*^d^*	—	Yes
*Dodecasatellite*: CGGTCCCGTACT or GGTCCCGTACT	*3*; centromeric*^e^*	—	No
359-bp repeat	*X*; noncentromeric*^b^*	—	No

a Lohe *et al.* (1993) and Jagannathan *et al.* (2017).

b Tolchkov *et al.* (2000).

c Sun *et al.* (2003).

d Torok *et al.* (1997, 2000), Blower and Karpen (2001) and Garavís *et al.* (2015).

e Garavís *et al.* (2015).

The centromere of the *D. melanogaster* minichromosome *Dp 1187* which is derived from *In*(*1*)*sc8*, an inversion of the *X* chromosome, was one of the first metazoan centromeres investigated in molecular detail (Le *et al.* 1995; Murphy and Karpen 1995; Sun *et al.* 1997, 2003). Deletion derivatives defined a 440-kb region that was necessary for full centromere function, and which encompassed complex DNA from transposons embedded in uniform arrays of the satellites AATAT and TTCTC (AAGAG) from left to right. Fluorescent *in situ* hybridization (FISH) mapping of AAGAG on the pericentric inversion *In*(*1LR*)*pn2b* demonstrated that this pentamer is present on the short right arm of the *X* chromosome (Koryakov *et al.* 1999). Heterochromatic banding patterns on a series of secondary rearrangements of the paracentric inversion *In*(*1*)*pn2a* mapped the centromere to the heterochromatic band corresponding to AATAT (Tolchkov *et al.* 2000). This cast doubt on whether the AAGAG satellite is required for normal *X* centromere function, and raised the question of whether AATAT is found at centromeres on other chromosomes. AATAT is distributed throughout chromosome *4*, but is generally undetectable by *in situ* hybridization on chromosome *2*, and does not appear to be centromeric on chromosome *3* (Lohe *et al.* 1993; Jagannathan *et al.* 2017); indicating that it is most likely not at the centromeres of these metacentric autosomes.

The centromere of chromosome *3* was mapped by using a *SuUR* mutant or the double mutant *SuUR Su*(*var*)*3-9^06^*, both of which suppress underreplication of heterochromatic sequences in salivary gland polytene chromosomes, and also by using *otu* mutants, which polytenize chromosomes of pseudonurse cells. The centromere was found to be the constriction between blocks of the 10-mer AATAACATAG and the 11- or 12-mer GGTCCCGTACT or CGGTCCCGTACT, known as *dodecasatellite* (Koryakov *et al.* 2002; Andreyeva *et al.* 2007; Garavís *et al.* 2015). The AATAACATAG 10-mer is also known as *Prodsat* because, during mitosis, it is specifically bound by the protein encoded by *proliferation disruptor* (*prod*) (Platero *et al.* 1998; Torok *et al.* 2000). *prod* mutants have defects in chromosome condensation near centromeres *2* and *3* and in anaphase chromatid separation (Torok *et al.* 1997). Simultaneous immunolocalization of the Prod protein with the *Drosophila* CENP-A protein found that they are immediately adjacent on chromosomes *2* and *3* (Blower and Karpen 2001), indicating that both proteins may occupy *Prodsat* at the centromeres of chromosomes *2* and *3*. Alternatively, colocalization of CENP-A with *dodecasatellite* on chromatin fibers suggested that *dodecasatellite* may be all or part of the centromere on chromosome *3* (Garavís *et al.* 2015).

In the sibling species *D. simulans*, which has been separated from *D. melanogaster* by ∼5 MY (Tamura *et al.* 2004), *dodecasatellite* is similarly near the centromeres of chromosomes *2* and *3* (Carmena *et al.* 1993; Jagannathan *et al.* 2017). This is in contrast to many other satellites, whose locations change dramatically in *D. simulans*.

A common method for identifying centromere sequences in other organisms has been chromatin immunoprecipitation (ChIP) of cenH3. Because immunoprecipitates (IPs) of ChIP experiments always have background DNA from the whole genome, centromere sequences are identified as those that are quantitatively enriched over the same sequences in the input DNA (Takahashi *et al.* 2000; Zhong *et al.* 2002; Nagaki *et al.* 2003, 2004; Marques *et al.* 2015). Here we use a native ChIP protocol to identify centromere sequences in *D. melanogaster* and *D. simulans*. We find that a few families of tandem repeats, including AATAT, AATAG, and *Prodsat*, are enriched at *D. melanogaster* centromeres. In *D. simulans*, we find larger complex tandem repeats at the centromeres. We show that centromere repeats have been expanding in both species. Our results indicate rapid divergence of centromeres in these species and suggest that small repeats are replacing older, complex repeats, especially in *D. melanogaster*.

## Materials and Methods

### Nomenclature

The *Drosophila* cenH3 variant is encoded by the *centromere identifier* (*cid*) locus. Early phylogenetic analyses failed to establish orthology of the Cid protein with the vertebrate cenH3 protein CENP-A (Malik and Henikoff 2003; Baker and Rogers 2006; Dawson *et al.* 2007; Postberg *et al.* 2010); however, recent sequencing of cenH3 genes from a broad range of insects supported their orthology with CENP-A (Drinnenberg *et al.* 2014), and here we refer to the Cid protein as CENP-A. We continue to refer to the antibodies used to bind *Drosophila* CENP-A as anti-Cid antibodies because they cross-react with CENP-A proteins only in *D. melanogaster* and close relatives. We use the traditional designations (Carmena *et al.* 1993; Lohe *et al.* 1993; Torok *et al.* 2000) for common short satellites (Table 1), and the terms “*simcent1*” and “*simcent2*” for newly identified centromere sequences in *D. simulans*.

### Fly stocks and cell lines

The embryonic cell lines S2-DRSC from *D. melanogaster* (hereafter S2) and ML82-19a from *D. simulans* were obtained from the *Drosophila* Genomics Resource Center, grown as recommended, and used for ChIP. S2 cells are approximately tetraploid for chromosomes *2* and *3*, triploid for the *X*, and diploid for chromosome *4*, with some variability (Lee *et al.* 2014). ML82-19a cells are diploid for autosomes with a single *X* chromosome (Supplemental Material, Figure S1A). Neither line has a *Y* chromosome, and our cell line experiments cannot address the nature of the *Y* centromere.

A *Cid-GFP* construct (Henikoff *et al.* 2000) was injected into *w^1118^* flies and a stable line, *P*[*Cid-GFP*]*8-10*, was obtained in which CENP-A-GFP localizes to *D. melanogaster* centromeres (Figure S1B). CENP-A-GFP is undetectable by Western blots in protein extracts of these flies using either anti-Cid or anti-GFP antibodies; however endogenous CENP-A is detected with anti-CidM antibody, which we take as evidence that CENP-A-GFP has a very low expression level. *T*(*1*;*3*)*e^H2^*, *e^v^/In*(*3R*)*C*, *e l*(*3*)*e* was previously described (Henikoff 1980). Oregon R (*D. melanogaster*) was obtained from the Bloomington Stock Center, and *w^501^* (*D. simulans*) was a gift from H. Malik.

### Antibodies, immunocytology, and microscopy

Two independent rabbit anti-Cid antibodies were used to confirm results in *D. melanogaster*. The anti-CidH antibody, raised to the epitope acetyl-CAKRAPRPSANNSKSPNDD-amide, has been previously described (Henikoff *et al.* 2000). The anti-CidM antibody, raised to the epitope MPRHSRAKRAPRPSA, is a gift from H. Malik. Although the amino acid sequences of CENP-A in both *D. melanogaster* and *D. simulans* are identical in the region of the epitopes, only anti-CidM localizes to centromeres in *D. simulans* tissue culture cells and larval brains (Figures S1 and S2). Chicken anti-GFP antibody was from ThermoFisher Scientific (Waltham, MA). Anti-phospho-H3S10 antibody was Millipore 06-570 (Billerica, MA).

Dissection and fixation of larval brains for antibody detection followed (Larracuente and Ferree 2015), with the omission of acetic acid, which interferes with detection of CENP-A for both anti-Cid antibodies. Slides were collected in PBS with Tween 20 (PBST), blocked with Odyssey Block or PBST plus 10% goat serum for 30 min, and then incubated for 1 hr at room temperature or overnight at 4° with anti-Cid or anti-GFP antibodies diluted 1:1000 in blocking solution. Slides were then washed 3× 5 min with blocking solution, incubated for 1 hr at room temperature with a fluorescent secondary antibody (Jackson ImmunoResearch) diluted 1:100 in blocking solution, washed again 3× 5 min in blocking solution or PBS, stained with DAPI solution, mounted with a cover glass using Vectashield, and sealed with nail polish. Antibody detection in tissue culture cells was similar, except that cells were allowed to adhere to coverslips for at least 1 hr before swelling with sodium citrate and spinning coverslips in a Cytospin 4 at 1900 rpm for 1 min before fixing in 2% formaldehyde for 15 min. Subsequent steps were the same as for brains, except carried out on coverslips. Chromosomes were visualized using a DeltaVision microscope and software (Applied Precision, now GE Healthcare). Images were false colored using ImageJ (Schneider *et al.* 2012).

### FISH

End-labeled fluorescent oligonucleotide probes and unlabeled probes were ordered from Eurofins Genomics (Louisville, KY). Unlabeled oligonucleotide probes were labeled with digoxigenin-11-dUTP (Roche) or biotin-16-dUTP using terminal deoxynucleotidyl transferase (Gibco/BRL) according to the manufacturer’s instructions. Probes for pentamers were 5′-(AATAT)_10_-3′, 5′-(AAGAG)_10_-3′, and 5′-[Alexa488]-(AATAG)_10_-3′. Probes for 10-mers were 5′-[Alexa594]-(AATAGAATTG)_3_-3′ and 5′-[Alexa594]- (AATAGAAGAG)_3_-3′_._ The *simcent1* probe was 5′-[Alexa488]-AGTAAGTACTTATGTTGTTTTGATAATCGGCAATCAGACTC-3′.

Larval brain squashes were prepared and fixed as for immunolocalization, except that the fixative was 2% formaldehyde, 45% acetic acid. Hybridization was carried out according to Larracuente and Ferree (2015), or as follows: chromosomes were denatured in 0.07 M NaOH for 3 min, followed by a wash in 2× SSC for 5 min, dehydrated in 70% ethanol 2× 5 min and 95% ethanol 2× 5 min, and then allowed to air dry. Slides were prehybridized with hyb mix (50% formamide, 10% dextran sulfate, 3× SSC buffer) for 30 min at 25 or 37°. Probes (∼30 ng in 2 μl) were heated at 100° for 3 min, mixed with 12 μl of hyb mix, added to the slide, covered with a cover glass, sealed with rubber cement, and incubated in a moist chamber at 25° overnight (18° for AATAT). Slides were washed 2× 30 min in 50% formamide, 2× SSC, and then 3× 5 min in PBS or 0.1× SSC. For probes labeled with digoxigenin or biotin, slides were incubated for 1 hr with anti-digoxigenin fluorescent antibody or streptavidin-rhodamine and washed 3× 5 min in PBS. Slides were stained, mounted, and sealed as above. For combined CENP-A detection and FISH, immunolocalization and FISH protocols were carried out successively.

### ChIP

Approximately 10^8^ S2 cells or ML82-19a cells were harvested per sample and subjected to native ChIP in buffer containing 0.05% SDS to solubilize the kinetochore as described (Skene and Henikoff 2017). MNase digestion was for 5 or 15 min at 37°. Protein G Dynabeads were from Invitrogen (Carlsbad, CA). For embryo ChIP, 0- to 8-hr embryos were collected, washed and dechorionated as described (Steiner *et al.* 2012), and stored at −80°. Approximately 1 g of frozen embryos per sample was ground in liquid nitrogen, and nuclei were prepared as described (Steiner *et al.* 2012). Purified nuclei were then processed for ChIP as above (Skene and Henikoff 2017). Camelid GFP-nAb beads were obtained from Allele Biotechnology (San Diego, CA). Sequencing libraries were prepared as described (Neiman *et al.* 2012) using 14 PCR cycles with a 10 sec, 60° combined annealing/extension step.

### Sequence analysis

The 250-bp, single-end reads from input and IP libraries of S2 cells were trimmed using Trim Galore! version 0.3.7 with the parameters: quality 20, adapter AGATCGGAAGAGC, stringency 3, phred 33, and length 25. For trimmed reads that occurred more than once, CD-HIT-EST version 4.6 (Fu *et al.* 2012) was used to cluster identical sequences, selecting the longest sequence, using the parameters -n 10 -r 1 -M 10000. The IP clusters with the most contributing reads were used to make reference sequences. Reads from input and IP libraries were mapped using Burrows–Wheeler Aligner (BWA) version 0.7.12-r1039 (Li and Durbin 2009) to the IP clusters, and the 100 clusters with the most mapped reads for input and IP were selected as reference sequences. For ML82-19a cells, reference sequences were generated from a 10-min MNase digest of chromatin. BWA was used to map 25- × 25-bp reads from input and IP libraries to the clusters. The 100 clusters with the most mapped reads for input and IP were selected as reference sequences. The reference sequences were analyzed for tandem repeats using the Tandem Repeats Finder server (https://tandem.bu.edu/trf/trf.html) with default parameters (Benson 1999).

The “grep” function of Unix was used to search for and count 9- to 16-mers and their reverse complements in raw Illumina sequencing reads. Counts of each candidate 9- to 16-mer were added to counts of its reverse complement, and then normalized to the total number of raw reads for either the input or IP libraries. Enrichment was calculated as the ratio of normalized IP counts to normalized input counts. Similar counts were made from sequencing data for *Drosophila* species downloaded from the Sequence Read Archive (SRA), normalizing to total reads to determine abundance. The National Center for Biotechnology Information Blast server was used to identify clones homologous to *simcent1* and *simcent2* sequences. Alignments were made using the Clustal Omega server of the European Molecular Biology Laboratory–European Bioinformatics Institute and adjusted by hand. Dot matrices were made with Dotter (Sonnhammer and Durbin 1995).

### Data availability

Fly stocks and antibodies are available upon request. Figure S1 shows cell line karyotypes and centromere staining with anti-CidM and anti-GFP. Figure S2 shows hybridization of AATAG and 10-mer probes to *D. simulans* neuroblasts. File S1 and File S2 contain reference sequences for input and IP for S2 cells. File S3 and File S4 contain the reference sequences for ML82-19a cells. File S5 and File S6 display alignments of reference sequences containing *simcent1* and *simcent2*, respectively. Table S1 lists the candidate 9- to 16-mers. Table S2 contains the SRA accessions counted for *Drosophila* species. Sequencing data are available at Gene Expression Omnibus with the accession number GSE105100.

## Results

### FISH mapping the *X* centromere of *D. melanogaster*

**Figure 1:**
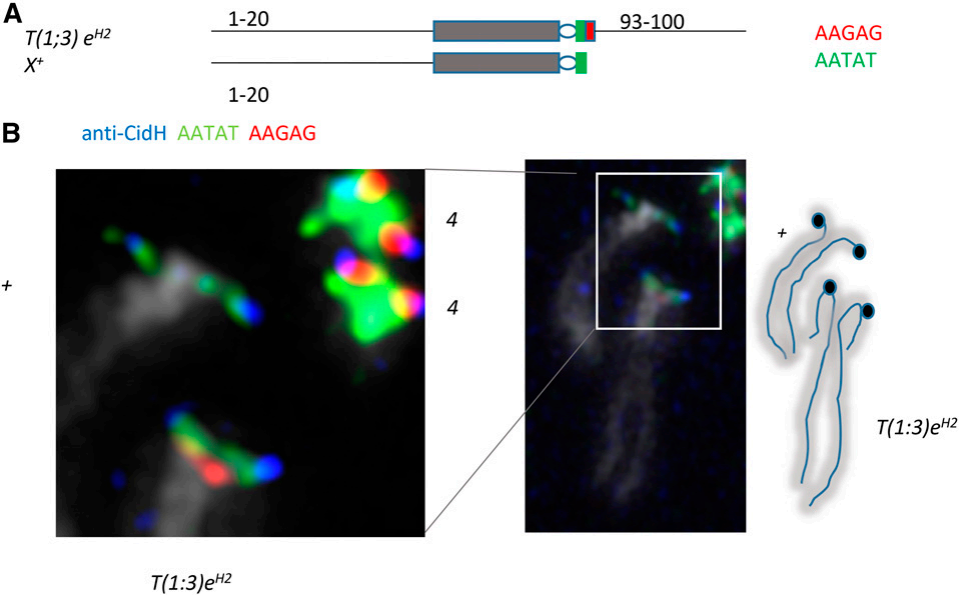
AAGAG is not necessary for centromere function on the *D. melanogaster X* chromosome. (A) Translocation *T*(*1*;*3*) *e^H2^* breaks on the short right arm of the *X* chromosome and at 93 on chromosome *3*. (B) The wild-type *X* in the stock *T*(*1*;*3*) *e^H2^/In*(*3R*)*C* lacks detectable AAGAG and CENP-A signal overlaps with AATAT signal. Hybridization signal is not found under all of the anti-CidH signal, suggesting that the kinetochore interferes with hybridization.

Previous studies had used chromosome rearrangements to map the *X* centromere in *D. melanogaster* to the heterochromatic band corresponding to a block of AATAT (Tolchkov *et al.* 2000), or used sequencing to map it to adjacent blocks of AATAT and CTCTT (= AAGAG) (Sun *et al.* 2003). To address this apparent discrepancy, we combined anti-CidH antibody staining with FISH to look at the translocation stock *T*(*1*;*3*)*e^H2^/+*, which has a rare break on the short right arm of the *X* chromosome (Figure 1A). Although the break does not separate AATAT and AAGAG repeats, fortuitously we found that the wild-type *X* chromosome in this stock lacks detectable AAGAG, which therefore appears to be unnecessary for the centromere. In chromosomes that appear to be under tension, the anti-CidH signal partially overlaps AATAT, apparently confirming that AATAT forms the *X* centromere (Figure 1B). In the same chromosome spread, partial overlap of AATAT, AAGAG, and anti-CidH signals on the fourth chromosome makes it unclear whether one or both of these pentamers are centromeric, underscoring the resolution limits of FISH for centromere mapping. We therefore turned to ChIP sequencing (ChIP-seq) to identify sequences directly bound by *Drosophila* CENP-A.

### *D. melanogaster* centromeres comprise 5- and 10-mers

ChIP for centromeres typically uses quantitative PCR to determine the enrichment of candidate centromeric repeats in the IP from an anti-cenH3 antibody, relative to the input DNA. This approach is not feasible when the candidate repeats are shorter than a typical PCR primer length. Instead, we used a counting approach to quantify the numbers of candidate sequence strings in raw reads in libraries made from the input and IP from ChIP experiments, using chromatin from the *D. melanogaster* cell line S2. To identify centromeric sequences in as unbiased a manner as possible, we selected as candidate sequences 9- to 16-mers (and their reverse complements) representing the 32 most abundant sequences identified in *D. melanogaster* by k-mers of 31 bp (Krassovsky and Henikoff 2014). The 9- to 16-mer candidate sequences consist of three or two tandem copies of 3- to 8-mer repeats or single copies of 10- to 15-mer repeats.

To identify additional candidate centromere sequences, we performed native ChIP-seq of CENP-A with 250-bp, single-end reads. Identical trimmed reads from the IP were clustered, selecting the longest read in a cluster. Both input and IP reads were mapped to the clusters (File S1 and File S2), ranking the clusters by the number of reads mapping to them, and selecting the top 100 clusters for both input and IP as reference sequences (Henikoff *et al.* 2015). Of the 100 reference sequences for the IP, 86 had at least two tandem copies of the *Prodsat* 10-mer AATAACATAG, whereas this sequence was in only 29 of the input reference sequences. A 20-mer of AATAG (four tandem copies) was found in five reference sequences of the IP compared with one input sequence. Together, these two repeats were found in 91 of the 100 IP reference sequences. We used Tandem Repeats Finder (Benson 1999) to identify additional repeats in the IP reference sequences. We found three tandem dimers or trimers that were unrepresented in the 32 most abundant short repeats and were apparently derived from and interspersed with *Prodsat*. We added these to our collection of candidate sequences, along with 10-mer negative controls selected from 5S DNA and from the complex 359-bp repeat family, which is not centromeric (Tolchkov *et al.* 2000; Sun *et al.* 2003). We also included 10-mer candidate centromere sequences identified in the *D. simulans* cell line ML82-19a (described further below). In total, we identified 74 9- to 16-mers and their reverse complements (Table S1) as candidate centromere sequences and controls.

We performed additional native ChIP-seq experiments with 25- × 25-bp paired-end reads, and counted the 9- to 16-mers in the raw reads of the inputs and IPs of all experiments. The 9- to 16-mers that were enriched at least twofold in the IPs of at least three experiments are shown in Table 2. The three most abundant sequences enriched in the IPs are *Prodsat*, AATAG, and AATAT; consistent with both our IP reference sequences and our FISH mapping of the *X* centromere. Other enriched candidates are also likely to be centromeric, but are more minor components, each comprising <5% of the number of counts for *Prodsat*, the most abundant centromeric sequence. The *dodeca* satellite, previously proposed to be part of the centromere of chromosome *3*, and the AAGAG repeat, found near the centromere of chromosome *2* and in *Dp1187* derived from the *X* chromosome (Sun *et al.* 2003; Garavís *et al.* 2015), are consistently depleted in our IPs. We therefore conclude that *Prodsat*, AATAG, and AATAT are the major components of *D. melanogaster* centromeres.

**Table 2:** Enrichment of sequences in CENP-A ChIP experiments in S2 cells

Repeat unit*^a^*	IP count*^b^*	IP/IN 1*^c^*	IP/IN 2*^d^*	IP/IN 3*^e^*	IP/IN 4*^b^*	Median	Minimum	Maximum
**AATAACATAG** **(*Prodsat*)**	15,020,471	3.06	14.78	6.76	8.96	7.9	3.1	14.8
**AATAGAATAG**	2,162,779	13.47	11.69	14.61	15.42	14	11.7	15.4
**AATATAATAT**	1,147,564	60.48	1.76	3.25	6.88	5.1	1.8	60.5
ATTATATTTT	527,674	2.04	0.98	2.03	3.03	2.0	0.98	3.0
AAGATAAGAT	90,458	4.74	0.91	9.1	2.67	3.7	0.9	9.1
AGAATAACATATAAC	68,335	11.24	36.35	61.09	89.7	48.7	11.2	89.7
ATAACATATAACAT	65,591	19.28	4.97	0.61	7.15	6.1	0.6	19.3

Candidate sequences enriched at least twofold in at least three experiments. Enrichment is the ratio of normalized IP counts to normalized input counts. IN, input.

a Counts of sequences not in bold are <5% of those of *Prodsat*.

b 15 min MNase, anti-CidM, 250-bp single-end reads.

c 5 min MNase, anti-CidM, 25- × 25-bp reads.

d 5 min 2 × MNase, anti-CidH, 25- × 25-bp reads.

e 15 min MNase, anti-CidM, 25- × 25-bp reads.

To verify that these three sequences represent centromeres in flies as well as in S2 cells, and that they can be immunoprecipitated using an independent epitope, we performed ChIP using an anti-GFP antibody on *P*[*Cid-GFP*]*8-10* embryos. These flies express a CENP-A-GFP fusion protein at a very low level, which localizes to centromeres (Figure S1B). Because anti-GFP is known to enrich for certain sequences in ChIP experiments (Teytelman *et al.* 2013), we also performed anti-GFP ChIP on wild-type Oregon R embryos as a control, and subtracted enrichment in Oregon R from enrichment in *P*[*Cid-GFP*]*8-10*. The sequences enriched at least twofold (Table 3) substantially match the results from S2 cells, including enrichment for AATAT, AATAG, AAGAT, and *Prodsat*. Since half of the embryos are male, it is possible that differences between the relative abundance of sequences in ChIP from embryos and from S2 cells reflect the inclusion of the *Y* centromere in embryos and aneuploidy in S2 cells, though it is also possible that these differences are simply experimental variation. By FISH, AATAG maps distally on the *Y*, while *Prodsat* was not found to be on the *Y*, and AATAT is known from multiple locations on the *Y* (Lohe *et al.* 1993); suggesting that either AATAT or AAGAT are likely candidates for the *Y* centromere. Enrichment of *Prodsat*, AATAT, AATAG, and AAGAT in both S2 cells and embryos using unrelated antibodies leads us to conclude that these sequences are major components of natural *D. melanogaster* centromeres.

**Table 3:** Enrichment of sequences in GFP ChIP from embryos

Repeat unit	IP count*^a^*	Cid-GFP IP/IN − OR IP/IN*^b^*
**AATAACATAG** (*Prodsat*)	371,367	6.75
**AATATAATAT**	215,558	4.44
**AAGATAAGAT**	53,939	13.05
**AATAGAATAG**	45,962	24.51
AATAGAATTG	5,061	3.18

Counts of AATAGAATTG (not in bold) are <5% of those of *Prodsat*. IN, input; OR, Oregon R.

a Counts from Cid-GFP IP.

b 5 min MNase, anti-GFP, 25- × 25-bp reads. Enrichment of sequences in the IP was calculated separately for Cid-GFP and Oregon R embryos, and then subtracted.

**Figure 2:**
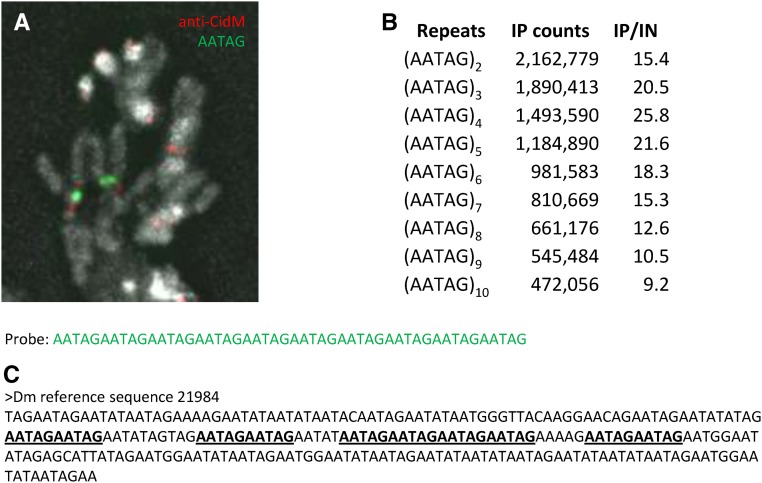
AATAG repeats in *D. melanogaster*. (A) A fluorescent (AATAG)_10_ probe detects repeats left of the centromere on chromosome *2* in larval brain squashes. (B) Enrichment of varying numbers of (AATAG)*_n_* repeats in 250-bp reads from anti-CidM ChIP of S2 cells. (C) An example of a reference sequence containing (AATAG)_2_ repeats.

Enrichment of AATAT in centromeres was expected both from our FISH mapping of the *X* centromere (Figure 1) and previous mapping (Tolchkov *et al.* 2000). Likewise, *Prodsat* has previously been mapped by FISH in or very near to the primary constrictions of chromosomes *2* and *3* (Platero *et al.* 1998; Koryakov *et al.* 2002), and *Drosophila* CENP-A abuts Prod protein (Blower and Karpen 2001), which binds to *Prodsat* (Torok *et al.* 2000). In contrast, AATAG was previously mapped by *in situ* hybridization using tritiated probes to the left of the centromere on chromosome *2*, on the distal long arm of the *Y* chromosome (which is absent in S2 cells), and in small amounts to chromosome *4* (Lohe *et al.* 1993). We confirmed using an (AATAG)_10_ fluorescent probe that the main site of AATAG hybridization on chromosome *2* is separated from the anti-Cid signal (Figure 2A).

To address whether AATAG is present at centromeres in interrupted arrays that might not hybridize to our (AATAG)_10_ probe, we counted *n*-mers of (AATAG) in the 250-bp, single-end reads, with *n* ranging from 2 to 10 (Figure 2B). Although all *n*-mers were enriched in the IP relative to input, the greatest enrichment was for *n* = 4, indicating that tandem arrays of AATAG do seem to be predominantly interrupted with mismatched sequences in the IP. While 69% of the (AATAG)_2_ 10-mers were also counted as (AATAG)_4_ 20-mers, only 22% were counted as (AATAG)_10_ 50-mers. Thus, we conclude that AATAG at centromeres is largely present as interrupted arrays rather than homogeneous arrays.

Four of the five (AATAG)_4_-containing reference sequences show interspersion of AATAG with other mismatched sequences, frequently AATAT, but not *Prodsat* (Figure 2C). The interrupting sequences generally maintain the 5-bp AANRN periodicity, as previously noted (Lohe and Brutlag 1987). In contrast to (AATAG)_10_ 50-mers, which are present in only one of the five (AATAG)_4_-containing reference sequences, (AATAACATAG)_5_ 50-mers occur in 68 of the 86 *Prodsat*-containing reference sequences. Only two of the *Prodsat*-containing reference sequences also have (AATAG)_2_, although the occurrence of a single AATAG among *Prodsat* repeats is fairly common. These patterns suggest that some or all of the (AATAG)_2_ counts in the ChIP may come from chromosome *4* (rich in AATAT), rather than chromosome *2* (rich in *Prodsat*).

### *D. simulans* centromeres are enriched in complex sequences

Since centromeres evolve quickly (Henikoff *et al.* 2001), we also performed ChIP-seq on centromeres from the ML82-19a cell line from the sibling species *D. simulans*, which diverged from *D. melanogaster* ∼5 MYA (Tamura *et al.* 2004). Although *D. simulans* shares several repeats with *D. melanogaster* (Lohe and Roberts 1988; Jagannathan *et al.* 2017), others are not shared, and there has been no investigation of what sequences might be centromeric in *D. simulans*. We digested chromatin from ML82-19a cells with MNase for 10 min, made a library, and sequenced 250-bp, single-end reads, clustering them as before. We then performed ChIP-seq on ML82-19a chromatin and mapped 25- × 25-bp, paired-end reads from a 15 min MNase digest onto the clusters, selecting the 100 clusters with the most mapped reads for both input and IP as reference sequences from which to identify candidate centromeric sequences (File S3 and File S4).

Two complex sequence families that we termed *simcent1* and *simcent2* were present in 46 and 14 of the IP reference sequences, respectively, and one reference sequence contained sequence homologous to both. Neither of these families was found in the reference sequences for the input. Using Tandem Repeats Finder, we found that in both the *simcent1* and *simcent2* families, some but not all reference sequences have a local tandem duplication of a core sequence of ∼76 bp (*simcent1*) or 44 bp (*simcent2*) within the longer complex sequence. We also found that the 10-mer AATAGAATTG was in five of the IP reference sequences and none of the input sequences; whereas the 10-mer AATAGAAGAG was present in 27 of the IP sequences, and in 30 of the input sequences. An abundant 15-mer GAACAGAACATGTTC was present in 36 of the input sequences but in only one IP reference sequence, where it was a minor component at the end of AATAGAAGAG repeats. (AATAG)_2_ was present in 17 input and 13 IP sequences, predominantly interspersed within AATAGAAGAG or AATAGAATTG repeats. The 10-mer (AAGAG)_2_ was present in 31 of the input and 17 of the IP sequences, usually interspersed with AATAGAAGAG or in the sequence (AAGAG)_2–3_AACAA. Thus, while the *D. simulans* IP reference sequences contain both complex and simple repeats, only *simcent1*, *simcent2*, and the 10-mer AATAGAATTG are present in more IP sequences than input sequences.

We made alignments of the *simcent1* and *simcent2* reference sequences (File S5 and File S6) to select 10-mers from these sequences to count in the raw reads from the ChIP. From the *simcent1* alignment, we selected 12 10-mers and their reverse complements, spanning a region of ∼200 bp that included the ∼76-bp duplicated sequence. From the *simcent2* alignment, we selected five 10-mers and reverse complements, spanning 66 bp including both the 44-bp duplication and flanking sequence. We also selected the 10-mers AATAGAAGAG and AATAGAATTG, 10-mers from the abundant 15-mer repeat and one of its variants, and all the 9- to 16-mers from the most abundant short repeats of *D. melanogaster* plus 5S and 359-bp repeat controls, so that the same sequences were counted in both *D. melanogaster* and *D. simulans* ChIPs (Table S1). Sequences enriched at least twofold in the anti-CidM IP of two experiments are shown in Table 4. The complex *simcent1* and *simcent2* sequences were highly enriched and were the most abundant centromere sequences. The variability in the abundance of 10-mers nearly adjacent to one another in the *simcent1* and *simcent2* alignments probably reflects the relative conserva]tion of individual 10-mers across the repeat family, since we counted only exact matches. Among 5-mer and 10-mer tandem repeats, only AATAGAATTG was twofold enriched in two experiments; although (AATAT)_2_, (AATAG)_2_, (AAGAG)_2_, and AATAGAAGAG were all twofold enriched in one experiment, along with the 8-mers (ATATACAT)_2_ and (AATAATAT)_2_. Three additional 7-mers were enriched in all three experiments, but were <5% as abundant as most of the *simcent1* and *simcent2* 10-mers (Table 4). We therefore conclude that *simcent1* and *simcent2* are the major centromere sequences in *D. simulans*, with some contribution from AATAGAATTG.

**Table 4:** Enrichment of sequences in CENP-A ChIP experiments in ML82-19a cells

10- to 14-mer*^a^*	IP counts*^b^*	IP/IN 1*^c^*	IP/IN 2*^c^*	IP/IN 3*^b^*	Repeat family
**ACAATCGTTT**	285,765	23.28	27.68	56.12	*simcent2*
**TTGCTTTGAG**	165,104	26.41	22.00	50.96	*simcent2*
**ACTGCAACGC**	149,863	20.52	20.66	39.06	*simcent2*
**TAATGGTTTT**	114,881	3.12	2.13	3.52	*simcent1*
**TTGTGTTTAC**	105,305	3.31	3.46	5.48	*simcent1*
**AGTACTTATG**	87,881	15.39	16.23	19.70	*simcent1*
**TGATAATCGG**	68,580	47.69	36.80	53.00	*simcent1*
**TTTAATATTA**	51,479	3.66	2.50	3.34	*simcent1*
**AAATAACACT**	50,478	3.74	4.07	4.88	*simcent1*
**ATTATATTTT**	48,229	3.56	2.35	4.16	*simcent1*
**CAATCAGACT**	38,353	35.92	30.76	38.84	*simcent1*
**AAAACTACTT**	34,493	2.49	2.09	5.64	*simcent2*
**AGCATGCAAC**	25,443	5.24	4.90	9.77	*simcent2*
**AATAGAATTG**	22,660	2.01	0.92	2.19	10-mer
**TCTGCGAGCC**	22,372	8.87	7.55	16.25	*simcent1*
ATTAGCGTTT	4,247	5.40	4.11	5.72	*simcent1*
AACAAATAACAAAT	1,278	5.56	2.75	6.36	7-mer
ATATAATATATAAT	238	19.70	2.11	2.15	7-mer
AATAGACAATAGAC	24	*^d^*	9.32	41.75	7-mer

IN, input.

a Candidate sequences enriched at least twofold in at least two experiments. Enrichment is the ratio of normalized IP counts to normalized input counts. Counts of sequences not in bold are <5% of those of ACAATCGTTT, the most abundant sequence counted in the IP.

b 15 min MNase, anti-CidM, 25- × 25-bp reads.

c 5 min MNase, anti-CidM, 25- × 25-bp reads.

d Enrichment undefined due to absence in input.

**Figure 3:**
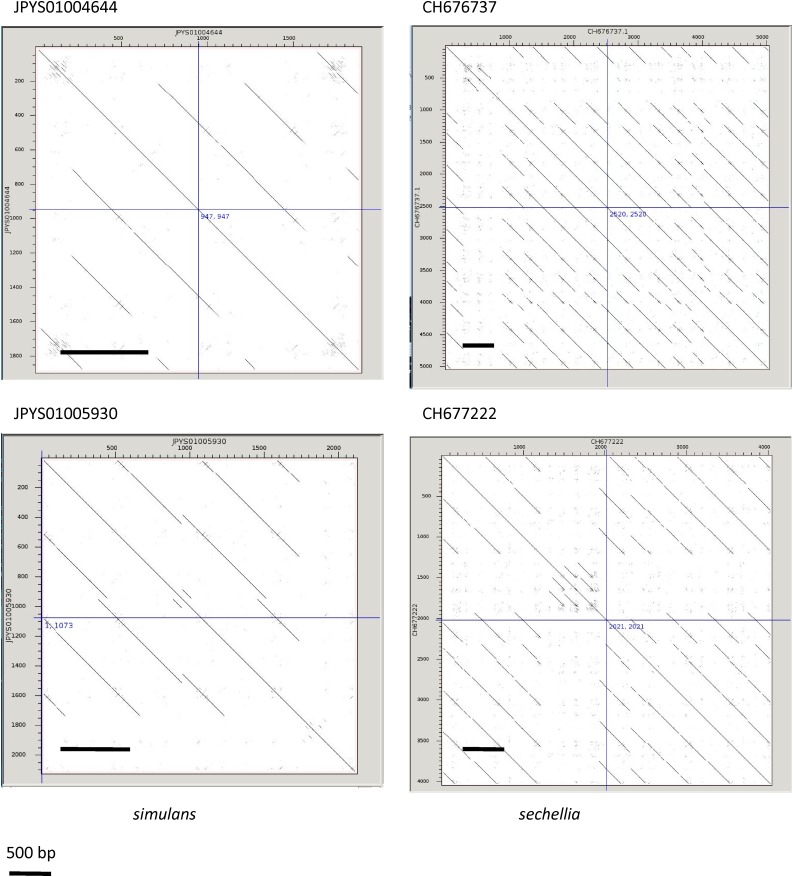
Periodicity of *simcent1* repeats. Dot matrix plots of similarity for four clones with homology to *simcent1* compared against themselves reveal repeats with a periodicity of ∼500 bp.

It is evident from the *simcent1* alignment that the *simcent1* sequence family exists in multiple configurations, and is subject to numerous indels. To better understand the nature of the *simcent1* family, we used blastn to search the *D. simulans* genome assembly with the core regions of the *simcent1* sequences to identify homologous clones. As in the reference sequences, the *simcent1* sequences in the clones exist in multiple complex arrangements, but a ∼500-bp periodicity can be discerned in clones JPYS01004644 and JPYS01000309 from *D. simulans* and in numerous clones from *D. sechellia* such as CH676566 and CH677222 (Figure 3). A 500-bp repeat present in *D. simulans* and the closely related *D. mauritiana* was previously described (Strachan *et al.* 1982), and we suggest that the *simcent1* family corresponds at least in part to this repeat family. The *simcent2* family is found in a 299-bp repeat in clone JPYS01006388, however the size is inconsistent in other clones such as JPYS01003943. This family appears to be absent in blast searches of the *D. melanogaster* and *D. sechellia* genomes.

**Figure 4:**
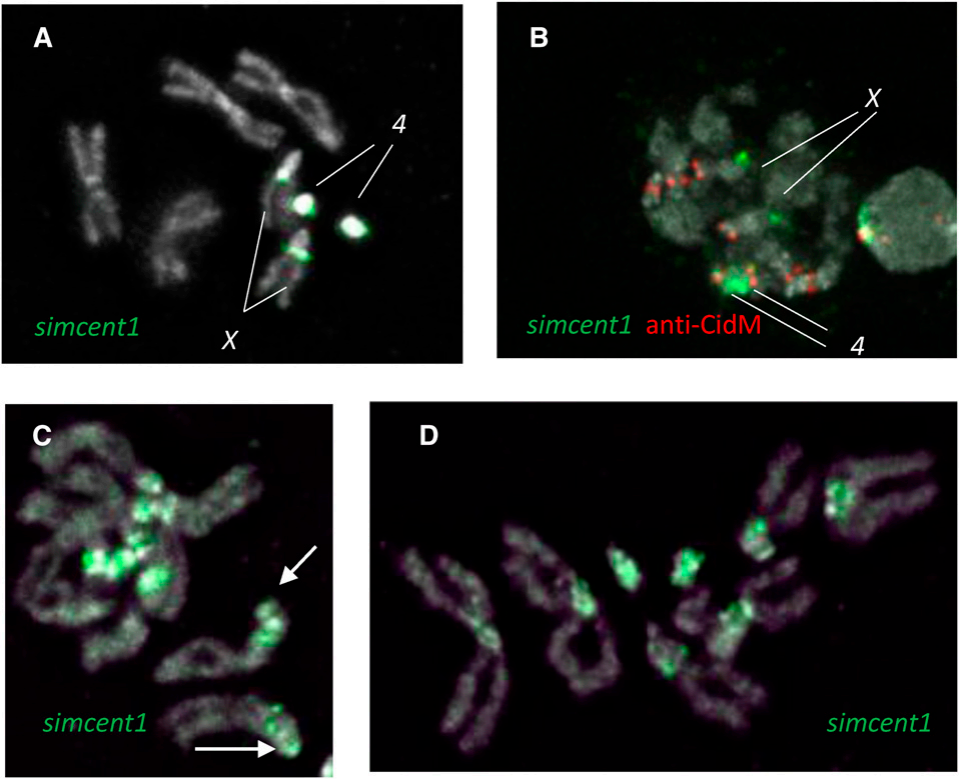
FISH of the *simcent1* probe in *D. simulans*. (A) Hybridization of *simcent1* to *D. simulans* larval neuroblasts using formaldehyde fixation. (B) *simcent1* hybridization together with CENP-A detection. (C and D) Hybridization of *simcent1* using formaldehyde fixation with 45% acetic acid. Arrow points to weak signal at the expected position of the *X* centromere.

We used the core tandem duplication in the *simcent1* sequence as a fluorescent probe for FISH to *D. simulans* chromosomes using formaldehyde fixation. This probe consistently hybridized to the *X* and *4* (Figure 4, A and B) in a pattern similar to that of a probe for (AATAG)_10_ (Figure S2, A and B). On the *X* chromosome, the *simcent1* probe seems to hybridize as dots on the side of the *X* heterochromatin, remarkably reminiscent of centromere signals (Figure 4A). However, these dots are not at the centromeres, which are at the apparent end of the *X* chromosome, as in *D. melanogaster* (Figure 4B). In contrast, the *simcent1* signal on chromosome *4* appears to touch the centromere. In hybridizations following fixation with formaldehyde and acetic acid, the *simcent1* probe also hybridized more weakly to all *D. simulans* chromosomes (Figure 4, C and D); suggesting that it is detecting a diverged repeat family present in the centric heterochromatin of all chromosomes. Hybridization at the tip of the *X* (Figure 4C) may indicate the presence of diverged repeats at the *X* centromere. We are unable to use acetic acid with either of the anti-Cid antibodies as it eliminates anti-Cid signal, and we believe this variable hybridization of the *simcent1* probe is the result of the absence or presence of acetic acid in the initial fixation, and that the weaker signal represents diverged repeats related to *simcent1* present on all chromosomes.

We also made 30-bp probes for the 10-mers AATAGAATTG and AATAGAAGAG. Perhaps consistent with finding these 10-mers are interspersed with (AATAG)_2_ in the reference sequences, the (AATAGAATTG)_3_ probe has a hybridization pattern similar to (AATAG)_10_, with stronger hybridization to the fourth chromosome (where it may be centromeric) than to the noncentromeric site on the *X* (Figure S2C). The (AATAGAAGAG)_3_ probe hybridized strongly to the *X* and the tip of the *Y*, with no visible hybridization to any centromere (Figure S2D). Together these results are consistent with the conclusion that *simcent1* is a major centromere component, while AATAGAATTG may be a centromere component on chromosome *4*.

### Different satellite repeats have expanded during divergence of *Drosophila* sibling species

**Figure 5:**
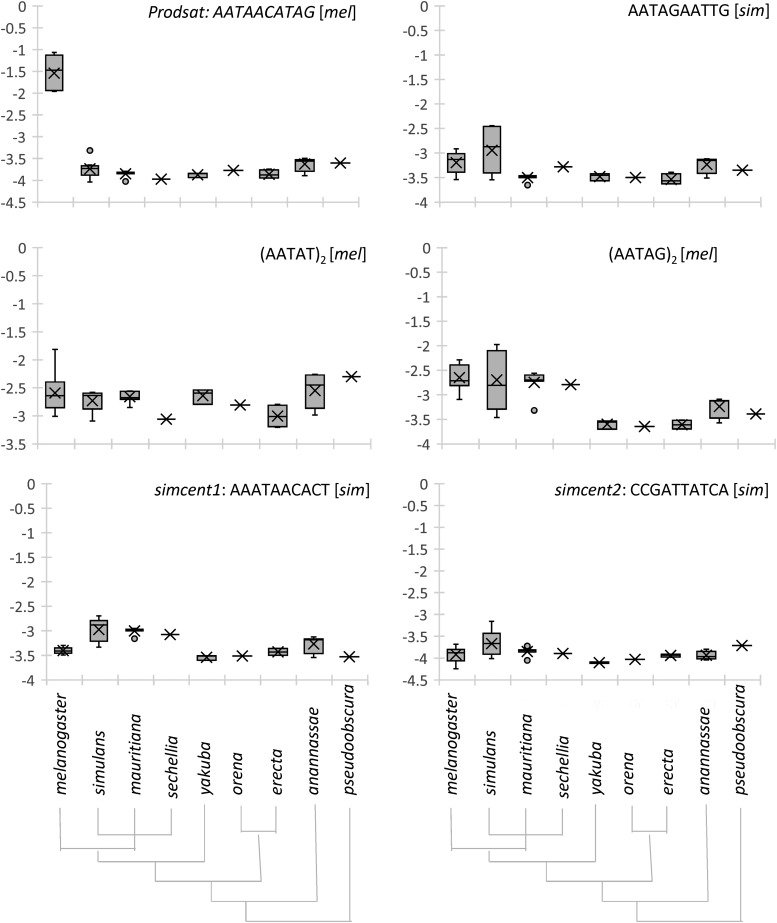
Abundance of simple and complex repeats in nine *Drosophila* species. All species are in the melanogaster subgroup, except *D. ananassae*, which is in the melanogaster group, and *D. pseudoobscura*, which serves as an outgroup. The cladograms at the bottom depict the relationships of the species. The *y*-axis represents the fraction of the reads that contain the repeat, presented on log_10_ scale. In the box and whisker plots, the box ends represent the second and third quartiles of the data separated by the median, while the whiskers represent the first and fourth quartiles. The mean is marked by X. For each centromere-enriched repeat, [*mel*] and [*sim*] indicate that in the corresponding species, the abundance of the repeat in anti-Cid IP was at least 5% of that of the most abundant centromere repeat in that species.

To better understand the evolution of the stark difference in centromeric repeats in *D. melanogaster* and *D. simulans*, we determined the abundance of these short and complex repeats in several *Drosophila* species with sequencing data available in the SRA (Figure 5). Although the amount of data differs for different species and there is substantial variation in data sets of the same species, several repeats appear to have expanded in the species in which they are enriched in centromeres. Most notably, *Prodsat* has expanded from its virtual absence in other species to comprise roughly 5 ± 2% of the genome in *D. melanogaster*. The *simcent1* family, though far less abundant, appears to have expanded in the recently diverged *simulans/sechellia/mauritiana* clade in comparison with more distantly related species. Both *simcent2* and AATAGAATTG have also expanded in *D. simulans*. (AATAG)_2_ and (AATAT)_2_ are slightly expanded in *D. melanogaster* compared with its closest relatives, although (AATAG)_2_ is comparable in *D. simulans*. Thus, most centromere sequences in these two species have undergone recent expansions.

## Discussion

We used ChIP with antibodies to the kinetochore protein CENP-A to identify sequences enriched in *Drosophila* centromeres. Our approach relied on counting short candidate centromere sequences to determine whether they are enriched at centromeres, and we therefore cannot exclude the possibility that there are other centromere sequences that we did not count. However, we think it is unlikely that there are other major centromere sequences because we would expect them to be captured in our 250-bp IP reference sequences.

In other organisms with known centromeric repeats, CENP-A typically occupies only a fraction of those repeats and, if this fraction is small, it will reduce the enrichment of repeats in ChIP experiments even if the ChIP is efficient. Thus the highly abundant *Prodsat* is less enriched than AATAG even though it is approximately seven times more abundant in the IP. We therefore consider both the abundance and enrichment of candidate sequences in the IP when judging their likely importance for centromere function.

A limitation of ChIP is that it cannot identify the chromosome(s) on which the enriched sequences occur. Because simple repeats are well mapped in *D. melanogaster*, our finding that AATAT and *Prodsat* are at centromeres is consistent with previous work (Torok *et al.* 1997; Tolchkov *et al.* 2000; Blower and Karpen 2001; Sun *et al.* 2003), suggesting that these repeats form the centromeres of chromosomes *X* (AATAT), *2*, and *3* (*Prodsat*). Enrichment of AATAG was not anticipated by previous work, and it is uncertain which chromosome(s) uses this sequence as centromere, with chromosomes *2* and *4* being the most likely possibilities. Based on the greater enrichment of (AATAG)_4_, over longer arrays, much of the centromeric AATAG does not appear to be in extended homogeneous tandem arrays. Frequent heterogeneity in some AATAG repeats has been previously described (Lohe and Brutlag 1987).

Two satellites previously proposed as centromeric, AAGAG and *dodecasatellite*, were consistently depleted in anti-Cid ChIP from S2 cells and *P*[*Cid-GFP*]*8-10* embryos. AAGAG was required for segregation of derivatives of the minichromosome *Dp1187* (Sun *et al.* 1997) and comprises the *bw^D^* heterochromatic element (Platero *et al.* 1998), which behaves as a neocentromere (Platero *et al.* 1999). The selection process for *Dp1187* or its derivatives could also have resulted in a neocentromere (Maggert and Karpen 2001). Fiber-FISH experiments found CENP-A on chromatin fibers containing *dodecasatellite* (Garavís *et al.* 2015). Although we do not dispute that such fibers occur, our data indicate that CENP-A bound to *dodecasatellite* is not a significant component of centromeres in S2 cells or *P*[*Cid-GFP*]*8-10* embryos. *Dodecasatellite* forms secondary structures (Garavís *et al.* 2015) and might be more sensitive to MNase, leading to its overall depletion in ChIP, but it is unclear why this would result in preferential depletion of *dodecasatellite* in the IP if it were a significant component of centromeres.

In stark contrast to the 5-mer and 10-mer centromeric sequences in *D. melanogaster*, the complex *simcent1* and *simcent2* repeats were the most abundant and enriched centromere sequences in *D. simulans*. Larger complex repeats are typical of centromeric sequences (Melters *et al.* 2013) and are found in other *Drosophila* species in the *buzzatii* and Hawaiian picture-wing complexes (Miklos and Gill 1981; de Lima *et al.* 2017), as well as in other insects (Lorite *et al.* 2004; Mravinac *et al.* 2004). The short repeat AATAGAATTG was also enriched at *D. simulans* centromeres. AATAGAATTG is found on the *X* near the nucleolus in a peculiar hybridization pattern on the side of the chromosome, raising the possibility that it participates in some unusual sequence organization or structure there. Based on this noncentromeric location on the *X*, AATAGAATTG in the IP of anti-CidM more likely derives from chromosome *4*. The weaker hybridization signal of *simcent1* to centromeric regions of all chromosomes when using acetic acid fixation is typical for diverged repeat families. The variable hybridization pattern of *simcent1* may reflect the combined effects of divergent sequences and reduced accessibility when omitting acetic acid in the initial formaldehyde fixation (Shapiro *et al.* 1978).

Our alignment of *simcent1* family sequences (File S5) reveals divergence in the form of base pair substitutions, small indels, and rearrangements juxtaposing different sequences. Complex sequence arrangements of satellites have also been observed in the *D. buzzatii* complex (Kuhn *et al.* 2009). Indeed, our 41-bp *simcent1* probe has seven to eight differences from the homologous sequences in the 500-bp repeat clone JPYS01004644. Although there is very limited cross-hybridization of the 500-bp repeats of *D. simulans* and *D. erecta* (Strachan *et al.* 1982), the *D. erecta* 500-bp repeats were also reported to hybridize to the centromeric regions of all chromosomes, including that of the acrocentric *X* where we see weak hybridization in *D. simulans* (Figure 4C); suggesting that this family may be ancestrally centromeric in the melanogaster subgroup.

Rapid change in animal and plant centromeres is thought to be a result of centromere drive, which was originally proposed to explain the rapid divergence of CENP-A between *D. melanogaster* and *D. simulans* (Malik and Henikoff 2001). Variant satellite arrays compete for recruitment of cenH3 and inclusion in the egg or megaspore during asymmetric female meiosis, in which only one meiotic product survives. It is therefore of interest that several centromere sequences– including *Prodsat* in *D. melanogaster*, and *simcent1*, *simcent2*, and AATAGAATTG in *D. simulans*– appear to be expanding over their relatively low levels in most sibling species in the melanogaster subgroup (Figure 5). In particular, *Prodsat* has expanded to become an order of magnitude more abundant than the other satellites counted here. It may have displaced the complex satellites present in *D. simulans*. What might be its advantage in centromere evolution? In rice, cenH3 nucleosomes exhibit rotational phasing of the DNA that wraps them (Zhang *et al.* 2013). A single turn of the DNA double helix is ∼10 bp, and sequences with 10-bp periodicity in WW dinucleotides (W = A or T) favor wrapping of nucleosomes by reducing the bending energy of wrapping (Struhl and Segal 2013). In particular, a 10-bp periodicity of AA dinucleotides, which is found in almost all 5-mer and 10-mer *Drosophila* satellites, is a “driving force” for nucleosome formation and minimizes bending energy (Prytkova *et al.* 2011), presumably stabilizing nucleosomes that may be under tension during anaphase. In addition to their 10-bp periodicity and sequences beginning with AA, an interesting feature of the centromere-enriched 5-mers and each half of the 10-mers is that almost all can be derived by a zero or one base change from AATAG (AAGAT requires two changes), probably reflecting sequence constraints on rotational phasing. Although short repeats are not common at centromeres, the 20-bp centromeric repeat of *Astragulus sinicus* (Tek *et al.* 2011) may serve the same proposed function as the 5- and 10-bp repeats of *Drosophila*.

If these repeats are advantageous for centromere formation, why are they not more common, particularly in other species of the melanogaster subgroup where they appear to have been present in low levels for millions of years? Rotational phasing at centromeres can be achieved without short repeats (Zhang *et al.* 2013), and a short repeat may need to reach a certain threshold expansion level in the right location before it can effectively recruit CENP-A nucleosomes to compete in female meiosis. In addition, if another protein binds to its sequence, its expansion may titrate the availability of the protein. The Prod protein binds specifically to *Prodsat* during mitosis in *D. melanogaster*, whereas it does not bind centric heterochromatin in *D. simulans* (Platero *et al.* 1998); suggesting that this binding is a new function that has become essential for condensation near the centromeres of chromosomes *2* and *3* (Torok *et al.* 1997) as *Prodsat* has expanded. Similarly, the complex 359-bp repeat, present in a large array on the *X* chromosome of *D. melanog*aster but in much smaller amounts on the autosomes of *D. simulans*, causes lethality in daughters of *D. melanogaster* males and *D. simulans* females because of mitotic defects associated with lagging 359-bp DNA during mitosis (Ferree and Barbash 2009); suggesting that a maternal protein in *D.melanogaster* is essential for proper condensation of the 359-bp repeats. Conversely, OdsH in *D. mauritiana* actively decondenses heterochromatin in *D. simulans* (Bayes and Malik 2009), leading to hybrid sterility. An imbalance between heterochromatic binding proteins and satellites may also be the cause of other hybrid incompatibilities and drive speciation (Satyaki *et al.* 2014). With this in mind, short repeats may be relatively uncommon because their amplification potentially provides tens of times more binding sites for matching DNA-binding proteins in the same length of DNA as more complex repeats, with potentially deleterious consequences.

## Supplementary Material

Supplemental material is available online at www.genetics.org/lookup/suppl/doi:10.1534/genetics.117.300620/-/DC1.
