# Supplementary material for: Simple and Complex Centromeric Satellites in Drosophila Sibling Species
Source: Genetics. 2018 Jan 5;208(3):977–90. doi: 10.1534/genetics.117.300620 (PMC5844345; doi:10.1534/genetics.117.300620)
Supplement: Supplementary file 1 [file 977FigureS1.pptx]

## Slide 1
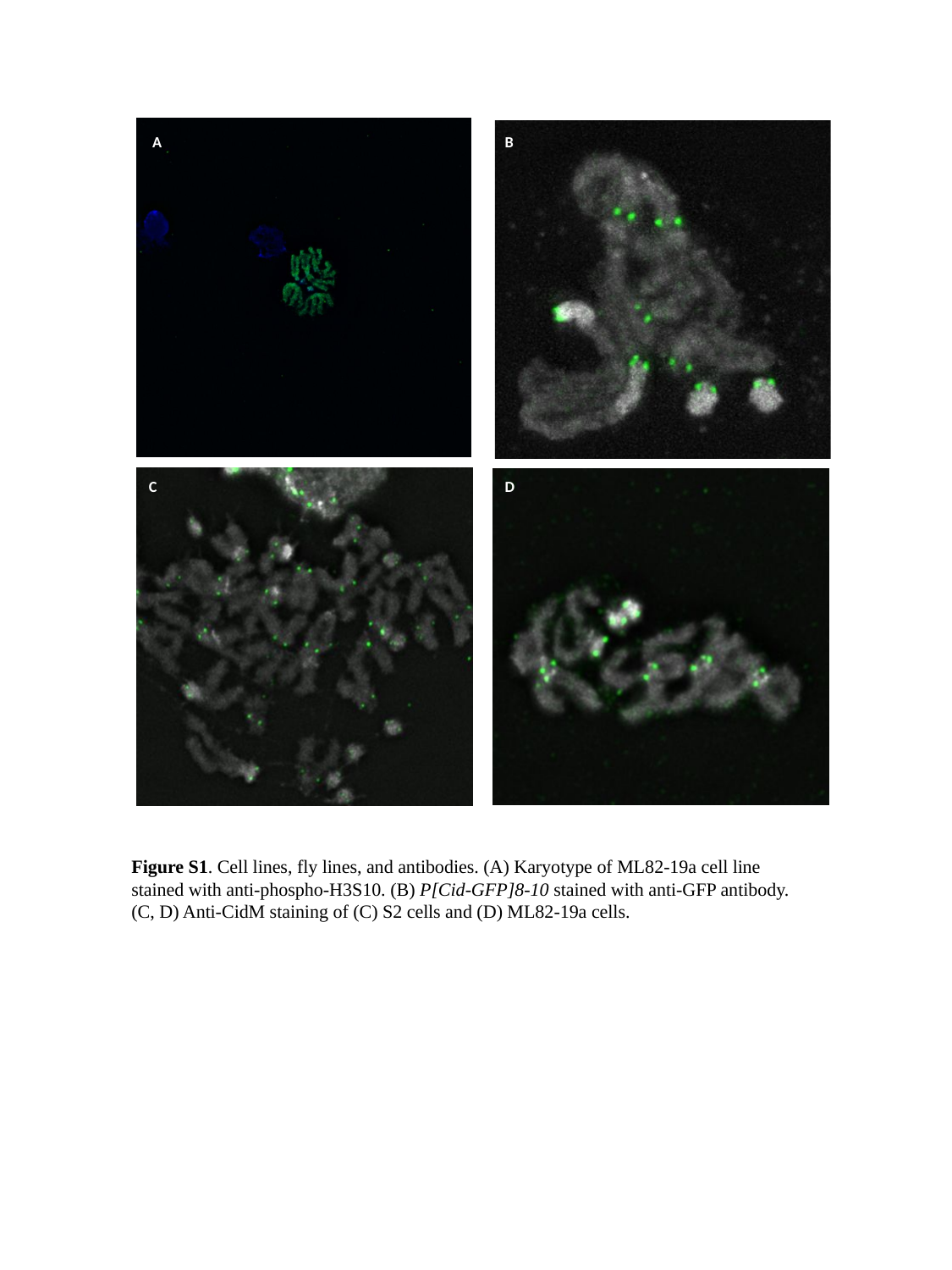

A
B
C
D
Figure S1. Cell lines, fly lines, and antibodies. (A) Karyotype of ML82-19a cell line stained with anti-phospho-H3S10. (B) P[Cid-GFP]8-10 stained with anti-GFP antibody. (C, D) Anti-CidM staining of (C) S2 cells and (D) ML82-19a cells.
