# Supplementary material for: Simple and Complex Centromeric Satellites in Drosophila Sibling Species
Source: Genetics. 2018 Jan 5;208(3):977–90. doi: 10.1534/genetics.117.300620 (PMC5844345; doi:10.1534/genetics.117.300620)
Supplement: Supplementary file 2 [file 977FigureS2.pptx]

## Slide 1
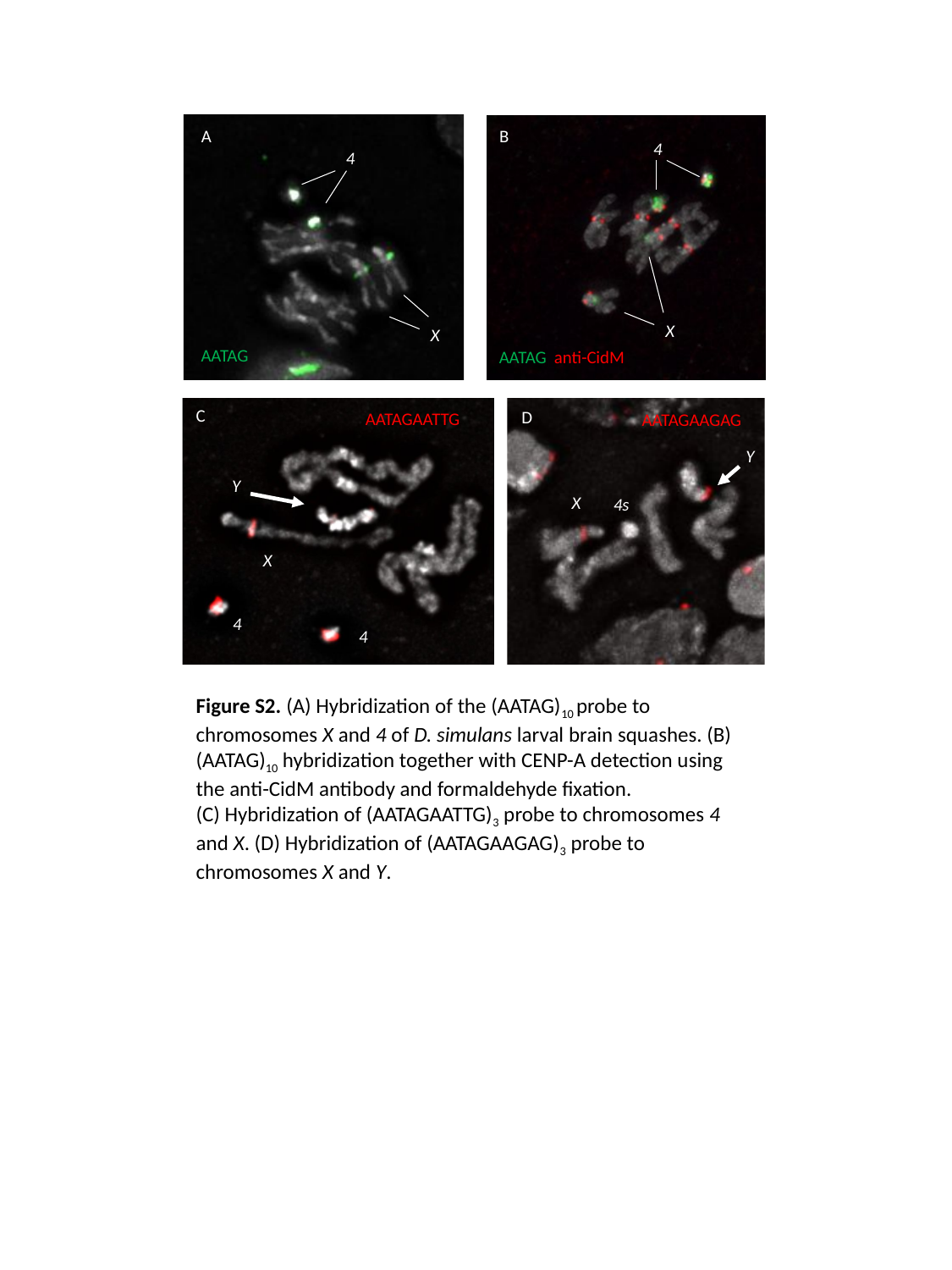

A
4
X
AATAG
B
4
X
AATAG anti-CidM
C
AATAGAATTG
Y
X
4
4
D
AATAGAAGAG
Y
X
4s
Figure S2. (A) Hybridization of the (AATAG)10 probe to chromosomes X and 4 of D. simulans larval brain squashes. (B) (AATAG)10 hybridization together with CENP-A detection using the anti-CidM antibody and formaldehyde fixation.
(C) Hybridization of (AATAGAATTG)3 probe to chromosomes 4 and X. (D) Hybridization of (AATAGAAGAG)3 probe to chromosomes X and Y.
