# Supplementary material for: Simple and Complex Centromeric Satellites in Drosophila Sibling Species
Source: Genetics. 2018 Jan 5;208(3):977–90. doi: 10.1534/genetics.117.300620 (PMC5844345; doi:10.1534/genetics.117.300620)
Supplement: Supplementary file 4 [file 977TableS2.docx]

**Table S2. *Drosophila* species SRA accessions utilized.**

| Species | SRA entry | Read length | Bases |
| --- | --- | --- | --- |
| melanogaster | SRR497721 | 2 x 101 | 2.3G |
| melanogaster | SRR1516224 | 2 x 101 | 10.1G |
| melanogaster | SRR1516222 | 2 x 101 | 13.8G |
| melanogaster | SRR1515985 | 2 x 101 | 16.4G |
| melanogaster | SRR643249 | 2 x 101 | 5.4G |
| melanogaster | SRR643252 | 2 x 101 | 4.5G |
| melanogaster | SRR643245 | 2 x 101 | 3.4G |
| melanogaster | SRR642763 | 2 x 101 | 4.8G |
| melanogaster | SRR643244 | 2 x 101 | 5.1G |
| simulans | SRR2859358 | 2 x 101 | 12.1G |
| simulans | SRR2859357 | 2 x 101 | 7.1G |
| simulans | SRR869579 | 2 x77 | 3.3G |
| simulans | SRR869580 | 2 x 77 | 1.6G |
| simulans | SRR2036958 | 2 x 102 | 6.6G |
| simulans | SRR2036251 | 2 x 77 | 2.9G |
| simulans | SRR520350 | 2 x 102 | 4.9G |
| simulans | SRR520334 | 2 x 105 | 9.7G |
| simulans | SRR1210633 | 2 x 101 | 40.1G |
| mauritiana | SRR1555246 | 2 x 101 | 18.2G |
| mauritiana | SRR1560267 | 2 x 77 | 6.2G |
| mauritiana | SRR1560268 | 2 x 77 | 6.4G |
| mauritiana | SRR1560269 | 2 x 77 | 6.1G |
| mauritiana | SRR1560100 | 2 x 77 | 4.2G |
| mauritiana | SRR1560102 | 2 x 77 | 5.9G |
| mauritiana | SRR1560103 | 2 x 77 | 6.8G |
| sechellia | SRR869587 | 2 x 77 | 4.2G |
| yakuba | SRR1198288 | 2 x 77 | 19.6G |
| yakuba | SRR1198295 | 2 x 77 | 12.3G |
| yakuba | SRR1198291 | 2 x 77 | 9.9G |
| orena | SRR1977592 | 2 x 102 | 3.7G |
| erecta | SRR1977589 | 2 x 102 | 11G |
| erecta | SRR1977582 | 2 x 102 | 6.7G |
| erecta | SRR1977539 | 2 x 102 | 4.1G |
| erecta | SRR1977503 | 2 x 102 | 2.7G |
| ananassae | SRR491387 | 2 x 102 | 9.1G |
| ananassae | SRR491373 | 2 x 76 | 4.8G |
| ananassae | SRR2126857 | 2 x 151 | 6.3G |
| ananassae | SRR2126916 | 2 x 151 | 6.6G |
| ananassae | SRR2135600 | 2 x 151 | 5G |
| ananassae | SRR2127151 | 2 x 151 | 6G |
| ananassae | SRR2127161 | 2 x 151 | 6.9G |
| ananassae | SRR2127156 | 2 x 151 | 4.7G |
| pseudoobscura | SRR1738164 | 2 x 101 | 5.2G |
