# Supplementary material for: Simple and Complex Centromeric Satellites in Drosophila Sibling Species
Source: Genetics. 2018 Jan 5;208(3):977–90. doi: 10.1534/genetics.117.300620 (PMC5844345; doi:10.1534/genetics.117.300620)
Supplement: Supplementary file 5 [file 977FileS1.docx]

**File S1. Top 100 IP reference sequences that map input reads from S2 cells.** The sequence names have the form: Reference number_number of reads contributing to the cluster_length of sequence.

>10_8059_250

AGAGAAGAGAAGAGAAGAGAAGAGAAGAGAAGAGAAGAGAAGAGAAGAGAAGAGAAGAGAAGAGAAGAGAAGAGAAGAGAAGAGAAGAGAAGAGAAGAGAAGAGAAGAGAAGAGAAGAGAAGAGAAGAGAAGAGAAGAGAAGAGAAGAGAAGAGAAGAGAAGAGAAGAGAAGAGAAGAGAAGAGAAGAGAAGAGAAGAGAAGAGAAGAGAAGAGAAGAGAAGAGAAGAGAAGAGAAGAGAAGAGAAGAGG

>124_1151_250

TAACATAGAATAACATAGAATAACATAGAATAACATAGAATAACATAGAATAACATAGAATAACATAGAATAACATAGAATAACATAGAATAACATAGAATAACATAGAATAACATAGAATAACATAGAATAACATAGAATAACATAGAATAACATAGAATAACATAGAATAACATAGAATAACATAGAATAACATAGAATAACATAGAATAACATAGAATAACATAGAATAACATAGAATAACATAGAA

>416_349_250

AGATAGATAGATAGATAGATAGATAGATAGATAGATAGATAGATAGATAGATAGATAGATAGATAGATAGATAGATAGATAGATAGATAGATAGATAGATAGATAGATAGATAGATAGATAGATAGATAGATAGATAGATAGATAGATAGATAGATAGATAGATAGATAGATAGATAGATAGATAGATAGATAGATAGATAGATAGATAGATAGATAGATAGATAGATAGATAGATAGATAGATAGATAG

>1135_168_250

TAGAATAGAATAGAATAGAATAGAATAGAATAGAATAGAATAGAATAGAATAGAATAGAATAGAATAGAATAGAATAGAATAGAATAGAATAGAATAGAATAGAATAGAATAGAATAGAATAGAATAGAATAGAATAGAATAGAATAGAATAGAATAGAATAGAATAGAATAGAATAGAATAGAATAGAATAGAATAGAATAGAATAGAATAGAATAGAATAGAATAGAATAGAATAGAATAGAATAGAA

>3417_94_250

TGTATATATGTATATATGTATATATGTATATATGTATATATGTATATATGTATATATGTATATATGTATATATGTATATATGTATATATGTATATATGTATATATGTATATATGTATATATGTATATATGTATATATGTATATATGTATATATGTATATATGTATATATGTATATATGTATATATGTATATATGTATATATGTATATATGTATATATGTATATATGTATATATGTATATATGTATATATG

>4574_80_250

AGAGAGAAGAGAGAAGAGAGAAGAGAGAAGAGAGAAGAGAGAAGAGAGAAGAGAGAAGAGAGAAGAGAGAAGAGAGAAGAGAGAAGAGAGAAGAGAGAAGAGAGAAGAGAGAAGAGAGAAGAGAGAAGAGAGAAGAGAGAAGAGAGAAGAGAGAAGAGAGAAGAGAGAAGAGAGAAGAGAGAAGAGAGAAGAGAGAAGAGAGAAGAGAGAAGAGAGAAGAGAGAAGAGAGAAGAGAGAAGAGAGAAGAGG

>15436_42_250

AAGACTGAAAATCAAATGAAAGTGAAAATGAGGAGAAGCCTCTGTGCTTGCTTAAAGAATATGTTAGTGTTTTTGGGAGAGCATTGCTCTTGGATTCTTAAGTGGAGGAGAAGCCTCAGAGAGAGCAGGCAGACTTTAAGATTTGTTTACAAGAATGGCTAAGTGCCGCTGTCAAAGAATTTGTTTATTAGAATGCATACTGCGCAGATGGCATACAGTATGGGGGTGTCAAAATGCAGAGTGGATATTT

>36149_26_250

ATAACATATAATAACATAGCATAACATAGAATAACATAGAATAACATATAATAACATAGAATAACAAGAATAACATAGAATAACATAGAATAACATAGAATAACATAGAATAACATAGAATAACATAGAATAACATAGAATAACATAGAATAACATAGAATAACATAGAATAACATAGAATAACATAGAATAACATAGAATAACATAGAATAACATAGAATAACATAGAATAACATAGAATAACATAGAA

>38664_25_250

ATGTTATATGTTATATGTTATATGTTATATGTTATATGTTATATGTTATATGTTATATGTTATATGTTATATGTTATATGTTATATGTTATATGTTATATGTTATATGTTATATGTTATATGTTATATGTTATATGTTATATGTTATATGTTATATGTTATATGTTATATGTTATATGTTATATGTTATATGTTATATGTTATATGTTATATGTTATATGTTATATGTTATATGTTATATGTTATATGTT

>44129_23_250

AGGTAGGCAGTGGTTGCCGACCTCTCATATTGTTCAAAACGTATGTGTTCATATGATTTTGGCAATTATATGAGTAAATTAAATCATATACATATGAAAATAAATATTTATTATATGTATATGGAAAAATGTTGAAATATTCCCATATTCTCTAAGTATTATAGAGAAAAGCCATTTTAGTGAATGGATATAGTAGTGTAAGCTAGCTGTTTTACGACAGAGGGTTCAAAAACTACTATAGGTAGGCAGG

>45439_23_250

TGATTAACGTTCAGCTTAAGACTGAAAATCAAATGAAAGTGAAAATGAGGAGAAGCCTCTGTGCTTGCTTAAAGAATATGTTAGTGTTTTTGGGAGAGCATTGCTCTTGGATTCTTAAGTGGAGGAGAAGCCTCAGAGAGAGCAGGCAGACTTTAAGATTTGTTTACAAGAATGGCTAAGTGCCGCTGTCAAAGAATTTGTTTATTAGAATGCATACTGCGCAGATGGCATACAGTATGGGGGTGTCAAA

>47735_22_250

ATATTATATTATATTATATTATATTATATTATATTATATTATATTATATTATATTATATTATATTATATTATATTATATTATATTATATTATATTATATTATATTATATTATATTATATTATATTATATTATATTATATTATATTATATTATATTATATTATATTATATTATATTATATTATATTATATTATATTATATTATATTATATTATATTATATTATATTATATTATATTATATTATATTATATT

>57609_20_250

TGTGTTGTTTATTGATTAACGTTCAGCTTAAGACTGAAAATCAAATGAAAGTGAAAATGAGGAGAAGCCTCTGTGCTTGCTTAAAGAATATGTTAGTGTTTTTGGGAGAGCATTGCTCTTGGATTCTTAAGTGGAGGAGAAGCCTCAGAGAGAGCAGGCAGACTTTAAGATTTGTTTACAAGAATGGCTAAGTGCCGCTGTCAAAGAATTTGTTTATTAGAATGCATACTGCGCAGATGGCATACAGTAG

>65385_18_250

AGACAAGACAAGACAAGACAAGACAAGACAAGACAAGACAAGACAAGACAAGACAAGACAAGACAAGACAAGACAAGACAAGACAAGACAAGACAAGACAAGACAAGACAAGACAAGACAAGACAAGACAAGACAAGACAAGACAAGACAAGACAAGACAAGACAAGACAAGACAAGACAAGACAAGACAAGACAAGACAAGACAAGACAAGACAAGACAAGACAAGACAAGACAAGACAAGACAAGACA

>70616_17_250

ACATAGAATAACATAGAACAACATAGAATAACATGGAATAACATAGAGTAACATAGAATAACATAGAATAACGTAGCAGAACACAGAATAACATAGAATAAAATAGCATAGCATAGAATAACAAAGAATAACATAGCATAACATAGAATAACATAGAATAACATAGAATAACATAGAATAACATAGAATAACATAGAATAACATAGAATAACATAGAATAACATAGAATAACATAGAATAACATAGAATA

>75072_17_250

TGGTAACATAGATCTAAGAATAACTGTTTTGAGCAGCTAATTACCAGTGCTAACGATCCCTATTACTTTTTGAAGGATTTAGGGAAATTAATTTTTGAATCAATTTTCGCATTTTTTGTAAGGAGGGGGGTCATCAAAATTTGCAAAATATGGCCAAAAAATTTAATTTCCATTTTTGAACACAGTTTGATTGGAAATTTTATTACAAGCTCAGTGAGGTATGCCATTCCATATTCAGACAATTATTTTT

>119378_12_250

ACACAGCATAACATAGAATAACATAGAATAACAAGAATAACATAGAATAACATAGAATAACATAGAATAACATAGAATAACATAGAATAACATAGAATAACATAGAATAACATAGAATAACATAGAATAACATAGAATAACATAGAATAACATAGAATAACATAGAATAACATAGAATAACATAGAATAACATAGAATAATATAGAATAACATAGAATAACATAGCATAACATAGAAACAAAGAATAACA

>127784_12_250

TATAAAATATAAAATATAAAATATAAAATATAAAATATAAAATATAAAATATAAAATATAAAATATAAAATATAAAATATAAAATATAAAATATAAAATATAAAATATAAAATATAAAATATAAAATATAAAATATAAAATATAAAATATAAAATATAAAATATAAAATATAAAATATAAAATATAAAATATAAAATATAAAATATAAAATATAAAATATAAAATATAAAATATAAAATATAAAATATAA

>147343_11_249

TGAAAATTAACAGTAACACTGGCGGTTTTATTTATAAACAATAGAATAACATAGAATAACATAGAATAACATAGAATAACATAGAATAACATAGAATAACATAGAATAACATAGAATAACATAGAATAACATAGAATAACATAGAATAACATAGAATAACATAGAATAACATAGAATAACATAGAATAACATAGAATAACATAGAATAACATAGAATAACATAGAATAACATAGAATAACATAGAATAA

>174643_10_250

TGTTCAAAACGTATGTGTTCATATGATTTTGGCAATTATATGAGTAAATTAAATCATATACATATGAAAATAAATATTTATTATATGTATATGGAAAAATGTTGAAATATTCCCATATTCTCTAAGTATTATAGAGAAAAGCCATTTTAGTGAATGGATATAGTAGTGTAAGCTAGCTGTTTTACGACAGAGGGTTCAAAAACTACTATAGGTAGGCAGTGGTTGCCGACCTCTCATATTGTTCAAAACG

>193918_9_250

TACCTCACTGAGCTTGTAATAAAATTTCCAATCAAACTGTGTTCAAAAATGGAAATTAAATTTTTTGGCCATATTTTGCAAATTTTGATGACCCCCCTCCTTACAAAAAATGCGAAAATTGATTCAAAAATTAATTTCCCTAAATCCTTCAAAAAGTAATAGGGATCGTTAGCACTGGTAATTAGCTGCTCAAAACAGTTATTCTTAGATCTATGTGACCATTTTTAGCCAAGTTATAACGAAAATTTCG

>218423_8_250

AGTAGTTTTTGAACCCTCTGTCGTAAAACAGCTAGCTTACACTACTATATCCATTCACTAAAATGGCTTTTCTCTATAATACTTAGAGAATATGGGAATATTTCAACATTTTTCCATATACATATAATAAATATTTATTTTCATATGTATATGATTTAATTTACTCATATAATTGCCAAAATCATATGAACACATACGTTTTGAACAATATGAGAGGTCGGCAACCACTGCCTACCTATAGTAGTTTTTG

>259009_7_250

AGAATATCTGTTTTGAGCGGCTAATTACCAGTGCTAACGATCCCTATTACTTTTTGATGGATTTAGGGAAATTAATTTTTGGATCAATTTTCGCATTTTTTGTAAGGAGGGGGGTCATCAAAATTTGCAAAATATGGCCAAAAAATTTAATTTCCATTTTTGAACACAGTTTGATTGGAAACTTTATTACGAGCTCAGTGAAGTATGACATTCTATATTCTGACAAAAAAATTTTAATGTTGCGGCAAAA

>259649_7_250

AGAGAATATGGGAATATTTCAACATTTTTCACTTATACACATAATAAATATTAATTTTCATATGTATATGATTTAATTTACTCATATAATTGCCAAAATCATATGAACACATACGTTTTGAACAATATGAGAGGTCGGCAACCACTGCCTACCTATAGTAGTTTTTGAACCCTCTGTCGTAGAACAGCTAGCTTACACTACTATATCCATTCACTAAAATGGCTTTTCTCTATAATACTTAGAGAATATG

>276143_7_250

TAACTCAGAATAACATAGAATAAGATAGAATAACATAGAATAACATAGAATAACATAGAATAACATAGAATAACATAGAATAACATAGAATAACATAGAATAACATAGAATAACATAGAATAACATAGAATAACATAGAATAACATAGAATAACATAGAATAACATAGAATAACATAGAATAACATAGAATAACATAGAATAGCAGAATAACAGAATAATAGAATAACAGAATAACTCAGAATAACATAG

>276301_7_250

TAATATAATAATATAATAATATAATAATATAATAATATAATAATATAATAATATAATAATATAATAATATAATAATATAATAATATAATAATATAATAATATAATAATATAATAATATAATAATATAATAATATAATAATATAATAATATAATAATATAATAATATAATAATATAATAATATAATAATATAATAATATAATAATATAATAATATAATAATATAATAATATAATAATATAATAATATAATA

>279215_7_250

TAGATTTACATAAAATTACATAGAAAAACATAGAATAACATAGAATAACATAGAATAACGTAGAATAACATAGAATAACATAGAATAACATATAATAACATAGATTTACATAGAATAACATAGAATAACATAGAATAACATAGAATAACATAGAATAACATAGAATAACATAGAATAACATAGAATAACATAGAATAACATAGAATAACATAGAATAACATAGAATAACATAGAATAACATAGAATAACA

>286516_7_250

TGGAAAAATGTTGAAATATTCCCATATTCTCTAAGTATTATAGAGAAAAGCCATTTTAGTGAATGGATATAGTAGTGTAAGCTAGCTGTTTTACGACAGAGGGTTCAAAAACTACTATAGGTAGGCAGTGGTTGCCGACCTCTCATATTGTTCAAAACGTATGTGTTCATATGATTTTGGCAATTATATGAGTAAATTAAATCATATACATATGAAAATAAATATTTATTATATGTATATGGAAAAATGT

>288319_7_250(

TGTGTATAAGTGAAAAATGTTGAAATATTCCCATATTCTCTAAGTATTATAGAGAAAAGCCATTTTAGTGAATGGATATAGTAGTGTAAGCTAGCTGTTCTACGACAGAGGGTTCAAAAACTACTATAGGTAGGCAGTGGTTGCCGACCTCTCATATTGTTCAAAACGTATGTGTTCATATGATTTTGGCAATTATATGAGTAAATTAAATCATATACATATGAAAATTAATATTTATTATGTGTATAAG

>325506_6_249

ATCCATTCACTAAAATGGCTTTTCTCTATAATACTTAGAGAATATGGGAATATTTCAACATTTTTCACTTATACACATAATAAATATTAATTTTCATATGTATATGATTTAATTTACTCATATAATTGCCAAAATCATATGAACACATACGTTTTGAACAATATGAGAGGTCGGCAACCACTGCCTACCTATAGTAGTTTTTGAACCCTCTGTCGTAGAACAGCTAGCTTACACTACTATATCCATTCA

>341282_6_249

TAATATAATATAATATAATATAATATAATATAATATAATATAATATAATATAATATAATATAATATAATATAATATAATATAATATAATATAATATAATATAATATAATATAATATAATATAATATAATATAATATAATATAATATATTATATTATATTATATTATATTATATTATATTATATTATATTATATTATATTATATTATATTATATTATATTATATTATATTATATTATATTATATTATATT

>347201_6_249

TATTATATTATATTATATTATATTATATTATATTATATTATATTATATTATATTATATTATATTATATTATATTATATTATATTATATTATATTATATTATATTATATTATATTATATTATATTATATTATATTATATTATATAATATAATATAATATAATATAATATAATATAATATAATATAATATAATATAATATAATATAATATAATATAATATAATATAATATAATATAATATAATATAATATA

>353894_6_250

TGAGTAAATTAAATCATATACATATGAAAATAAATATTTATTATATGTATATGGAAAAATGTTGAAATATTCCCATATTCTCTAAGTATTATAGAGAAAAGCCATTTTAGTGAATGGATATAGTAGTGTAAGCTAGCTGTTTTACGACAGAGGGTTCAAAAACTACTATAGGTAGGCAGTGGTTGCCGACCTCTCATATTGTTCAAAACGTATGTGTTCATATGATTTTGGCAATTATATGAGTAAATTA

>405117_5_250

AGGGATAAAGCACAAATAAATTAAAAAAAAAATACATAGAATAACATATAATAACATAGAATAACAAGAATAACATAGAATAACATAGAATAACATAGAATAACATAGAATAACATAGAATAACATAGAATAACATAGAATAACATAGAATAACATAGAATAACATAGAATAACATAGAATAACATAGAATAACATAGAATAACATAGAATAACATAGAATAACATAGAATAACATAGAATAACATAGAA

>444655_5_250

TATGATTTTGGCAATTATATGAGTAAATTAAATCATATACATATGAAAATAAATATTTATTATATGTATATGGAAAAATGTTGAAATATTCCCATATTCTCTAAGTATTATAGAGAAAAGCCATTTTAGTGAATGGATATAGTAGTGTAAGCTAGCTGTTTTACGACAGAGGGTTCAAAAACTACTATAGGTAGGCAGTGGTTGCCGACCTCTCATATTGTTCAAAACGTATGTGTTCATATGATTTTGG

>462754_5_250

TGTGTTGTGTTGTGTTGTGTTGTGTTGTGTTGTGTTGTGTTGTGTTGTGTTGTGTTGTGTTGTGTTGTGTTGTGTTGTGTTGTGTTGTGTTGTGTTGTGTTGTGTTGTGTTGTGTTGTGTTGTGTTGTGTTGTGTTGTGTTGTGTTGTGTTGTGTTGTGTTGTGTTGTGTTGTGTTGTGTTGTGTTGTGTTGTGTTGTGTTGTGTTGTGTTGTGTTGTGTTGTGTTGTGTTGTGTTGTGTTGTGTTGTGG

>474310_5_250

TGTTCAAAACGTATGTATTCATATGATTTTGGCAATTATATGAGTAAATTAAATCATATACATATGAAAAAGGCAGTGGTTGCCGACCTCTCATATTGTTCAAAACGTATGTGTTCATATGATTTTGGCAATTATATGAGTAAATTAAATCATATACATATGAAAATTAATATTTATTATATGTATAAGTGAAAAATATTGAAATATTCCCATATTCTCTAAGTATTATAGAGAATATAATTAATATATA

>512675_4_250

ACATATGAAAATGAATATTTATTATATGTATATGGAAAAATGTTGAAATATTCCCATATTCTCTAAGTATTATAGAGAAAAGCCATTTTAGTGAATGGATATAGTAGTGTAAGCTAGCTGTTTTACGACAGAGGGTTCAAAAACTACTATAGGTAGGCAGTGGTTGCCGACCTCTCATATTGTTCAAAACGTATGTGTTCATATGATTTTGGCAATTATATGAGTAAATTAAATCATATACATATGAAAA

>530032_4_249

AGACTAACATAGAATAAAATAGAATAACATTGACTAATATCGAATAACATAGAATAACATAGAATAACATAGAATAACATAGAATAACATAGAATAACATAGAATAACATAGAATAACATAGAATAACATAGAATAACATGGAATAACATAGAATAACATAGAATAACATAGAATAACATAGAATAACATAGAATAACATAGAATAACATAGAATAACATCGAATAACATAGAATATCACAGAATAACA

>533956_4_250

AGCCAAACACCTCGTCATTAACTACTAAAATAGGTAGGCAGTGGTTGCCGACCTCTCATATTGTTCAAAACGTATGTATTCATATGATTTTGGCAATTATATGAGTAAATTAAATCATATACATATGAAAAAGGCAGTGGTTGCCGACCTCTCATATTGTTCAAAACGTATGTGTTCATATGATTTTGGCAATTATATGAGTAAATTAAATCATATACATATGAAAATTAATATTTATTATATGTATAAG

>535973_4_250

AGGGATCGCTAGCACTGGTAATTAGCAGCTGCTCAAAACAGTTATTCCTACATCTATGTGACCATTTTTAGCCAAGTTATAACGAAAATTTGGTTTGTAAATATCAACATTTTTGCAGAGTCTGTTTTTCCAAATTTCGGTCATCAAATAATCATTTATTTTGCCACAACATAAAAAATAATTGTCTGAATATGGAATGTCATACCTCACTGAGCTCGTAATAAAATTTCCAATCAAACTGTGTTCAAAA

>535981_4_249

AGGGATCGTTAGCACTGGTAATAAGCTGCTCAAAACAGTTATTCTTACATCTATGTGACCATTTTTAGCCAAGTTATAACGAAAATTTCGTTTGTAAATATCAACATTTTTGCAGAGTCTGTTTTTCCAAATTTCGGTCATCAAATAATCATTTATTTTGCCACAACATAAAAAATAATTGTCTGAATATGGAATGTCATATCAAAACAGTTATTCTTACATCTATGTGACCATTTTTAGCGAAGTTAT

>537506_4_250

AGTAGTTTTTGAACCCTCTGTCGTAGAACAGCTAGCTTACACTACTATATCCATTCACTAAAATGGCTTTTCTCTATAATACTTAGAGAATATGGGAATATTTCAACATTTTTCACTTATACACATAATAAATATTAATTTTCATATGTATATGATTTAATTTACTCATATAATTGCCAAAATCATATGAACACATACGTTTTTAACAATATGAGAGGTCGGCAACCACTGCCTTTTTCATATGTATATG

>587489_4_250

TAACTTGGCTAAAAATGGTAACATAGATCTAAGAATAACTGTTTTGAGCAGCTAATTACCAGTGCTAACGATCCCTATTACTTTTTGAAGGATTTAGGGAAATTAATTTTTGAATCAATTTTCGCATTTTTTGTAAGGAGGGGGGTCATCAAAATTTGCAAAATATGGCCAAAAAATTTAATTTCCATTTTTGAACACGGTTTGATTGGAAATTTTATTACAAGCTCAGTGAGGTATGCCATTCCATATT

>597335_4_250

TAGAATATCACAGAATAACATAGACTCACATAGAATAAAATAGAATAACATAAAATAACATAGAATAACATAGAATAACATAGAATAACATAGAATAACATAGAATAACATAGAATAACATAGAATAACATAGAATAACATAGAATAACATAGAATAACATAGAATAACATAGAATAACATAGAATAACATAGAATATCACAGAATAACATAGACTAACATAGAATAACATAGAATAACATAAAATAACA

>599950_4_250

TAGGTCTTCAAATAATCATTTATTTTGCCGCAACATTAAAATTTTTTTGTCAGAATATAGAATGTCATACTTCACTGAGCTCGTAATAAAGTTTCCAATCAAACTGTGTTCAAAAATGGAAATTAAATTTTTTGGCCATATTTTGCAAATTTTGATGACCCCCCTCCTTACAAAAAATGCGAAAATTGATCCAAAAATTAATTTCCCTAAATCCATCAAAAAGTAATAGGGATCGTTAGCACTGGTAATT

>619858_4_250

TGACATTCCATATTCAGACAATTATTTCGTATGTTGTGGCAAAATAAATGATTATTTGATGACCGAAATTTGGAAAAACAGACTCTGCAAAAATGTTGATATTTACAAACGAAATTTTCGTTATAACTTGGCTAAAAATGGTCACATAGATGTAAGAATAACTGTTTTGAGCAGCTAATTACCAGTGCTAACGATCCCTATTACTTTTTGAAGGATTTTGGGAAATTAATTTTTGGATCAATTTTCGCAT

>627529_4_250

TGGGAATATTTCAACATTTTTCCATATACATATAATAAATATTCATTTTCATATGTATATGATTTAATTTACTCATATAATTGCCAAAATCATATGAACACATACGTTTTGAACAATATGAGAGGTCGGCAACCACTGCCTACCTATAGTAGTTTTTGAACCCTCTGTCGTAAAACAGCTAGCTTACACTACTATATCCATTCACTAAAATGGCTTTTCTCTATAATACTTAGAGAATATGGGAATATTT

>630109_4_249

TGTCCCTATCATATAATTAATATATAAAGAATTTAAAGAATTTTATCAAGAGTAGCCAAACACCTCGTCATTAACTACTATAATAGGTAGGCAGTGGTTGCCGACCTCTCATATTGTTCAAAACGTATGTATTCATATGATTTTGGCAATTATATGAGTAAATTAAATCATATACATATGAAAAAGGCAGTGGTTGCCGACCTCTCATATTGTTCAAAACGTATGTGTTCATATGATTTTGGCAATTAT

>657787_3_249

AAAGAATTTTATCAAGAGTAGCCAAACACCTCGTCATTAACTACTATAATAGGTAGGCAGTGGTTGCCGACCTCTCATATTGTTCAAAACGTATGTATTCATATGATTTTGGCAATTATATGAGTAAATTAAATCATATACATATGAAAAAGGCAGTGGTTGCCGACCTCTCATATTGTTCAAAACGTATGTGTTCATATGATTTTGGCAATTATATGAGTAAATTAAATCATATACATATGAAAATTA

>661463_3_250

AACACTGGCGGTTTTATTTATAAACAATAGAATAACATAGAATAACATAGAATAACATAGAATAACATAGAATAACATAGAATAACATAGAATAACATAGAATAACATAGAATAACATAGAATAACATAGAATAACATAGAATAACATAGAATAACATAGAATAACATAGAATAACATAGAATAACATAGAATAACATAGAATAACATAGAATAACATAGAATAACATAGAATAACATAGCATAACATAG

>668883_3_250

AACATAGAATAACATAGAATAACATAGAATAACATAGAATAACATAGAATAACATAGAATAACATAGAATAACATAGAATAACATAGAATAACATAGAATAACATAGAATAACATAGAATAACATAGAATAACATAGAATAACACAGAATAACATGGAATAGAATAGAATAGAAAGAATAGAATAGAGAAGAAAGAATAGAATAGAATAGAATAACACAGAATAACAAAGAATAACATAGAATAACATAG

>717811_3_250

ACATAGAATATCACAGAATAACATAGACTAACATAGAATAACATAAAATAACAAAGAATAACAAAGAATAACAAAGAATAACATAGAATAACATAGAATAACATAAAATAACAAAGAATAACAAAGAATAACAAAGAATAACATAGAATAACATAGAATAACATAGAATAACATAGAATAACATAGAATAACATAGAATAACATAGAATAACATAGAATAACATAGAATAACATAGAATAACATAGAATA

>726601_3_250

AGAAAGAATAGAATAGAAAGAATAGAATAGAATATAATAACATAGAATAACATAGAATAACATAGAATAACATAGAATAACATAGAATAACATAGAATAACATAGAATAACATAGAATAACATAGAATAACATAGAATAACATAGAATAACATAGAATAACATAGAATAACATAGAATAACATAGAATAACATAGAATAACATAGAATAACATAGAATAACATAGAATAACATAGAATAACATAGAATAA

>755592_3_250

AGAGGGTTCAAAAACTACTATAGGTAGGCAGTGGTTGCCGACCTCTCATATTGTTCAAAACGTATGTGTTCATATGATTTTGGCAATTATATGAGTAAATTAAATCATATACATATGAAAATTAATATTTATTATGTGTATAAGTGAAAAATGTTGAAATATTCCCATATTCTCTAAGTATTATAGAGAAAAGCCATTTTAGTGAATGGATATAGTACTGTAAGCTAGCTGTTCTACGACAGAGGGTTCA

>765423_3_250

AGGTAGGCAGTGGTTGCCGACCTCTCATATTGTTCAAAACGTATGTATTCATATGATTTTGGCAATTATATGAGTAAATTAAATCATATACATATGAAAAAGGCAGTGGTTGCCGACCTCTCATATTGTTCAAAACGTATGTGTTCATATGATTTTGGCAATTATATGAGTAAATTAAATCATATACATATGAAAATTAATATTTATTATATGTATAAGTGAAAAATATTGAAATATTCCCATATTCTCT

>780019_3_249

ATAACATAGATTAACATAGAATAACATGGAATAGAAAGAATAGAATAGAAAGAATAGAATAGAATATAATAACATAGAATAACATAGAATAACATAGAATAACATAGAATAACATAGAATAACATAGAATAACATAGAATAACATAGAATAACATAGAATAACATAGAATAACATAGAATAACATAGAATAACATAGAATAACATAGAATAACATAGAATAACATAGAATAACATAGAATAACATAGAA

>791507_3_250

ATCACAGAATAACATAGAATATCACAGAATAACATAGAATAACGTAGAATAACATAGAATAACATAGAATAACATAGAATAACATAGAATAACATAGAATAACATAGAATAACATAGAATAACATAGAATAACATAGAATAACATAGAATAACATAGAATAACATAGAATAACATAGAATAACATAGAATAACATAGAATAACATTGTTCACACATGAACACGAATATATTTAAAGACTTACAATTTTGG

>918498_3_250

TCTCTAAGTATTATAGAGAAAAGCCATTTTAGTGAATGGATATAGTAGTGTAAGCTAGCTGTTTTACGACAGAGGGTTCAAAAACTACTATAGGTAGGCAGTGGTTGCCGACCTCTCATATTGTTCAAAACGTATGTGTTCATATGATTTTGGCAATTATATGAGTAAATTAAATCATATACATATGAAAATGAATATTTATTATATGTATATAGGGGAAAAAATAATCATATAATATATATGAATAATG

>925949_3_250

TGACGAGGTGTTTGGCTACTCTTGATAAAATTCTTTATATATTAATTATATTCTCTATAATACTTAGAGAATATGGGAATATTTCAATATTTTTCACTTATACATATAATAAATATTAATTTTCATATGTATATGATTTAATTTACTCATATAATTGCCAAAATCATATGAACACATACGTTTTGAACAATATGAGAGGTCGGCAACCACTGCCTTTTTCATATGTATATGATTTAATTTACTCATATAA

>927008_3_250

TGAGTAAATTAAATCATATACATATGAAAAAGGCAGTGGTTGCCGACCTCTCATATTGTTAAAAACGTATGTGTTCATATGATTTTGGCAATTATATGAGTAAATTAAATCATATACATATGAAAATTAATATTTATTATGTGTATAAGTGAAAAATGTTGAAATATTCCCATATTCTCTAAGTATTATAGAGAAAAGCCATTTTAGTGAATGGATATAGTAGTGTAAGCTAGCTGTTCTACGACAGAGG

>929137_3_250

TGCAAAAATGTTGATATTTACAAACGAAATTTTCGTTATAACTTTGCTAAAAATGGTCACATAGATGTAAGATGTTTTGAGCTGCTAATTACCAGTGCTAACGATCCCTATTACTTTTTGAAGGATTTAGGAAATTAATTTTTGGATCAATTTTCGCATTTTTTGTAAGGAGGGGGGTCATCAAAATTTGCAAAATATGGCCAAAAAATTTCATTTCCATTTTTGAACACAGTTTGATTGGAAATTTTAT

>930038_3_250

TGCCAAAATCATATGAACACATACGTTTTGAACAATATGAGAGGTCGGCAACCACTGCCTTTTTCATATGTATATGATTTAATTTACTCATATAATTGCCAAAATCATATGAATACATACGTTTTGAACAATATGAGAGGTCGGCAACCACTGCCTACCTATTTTAGTAGTTAATGACGAGGTGTTTGGCTACTCTTGATAAAATTCTTTATATATTAATTATATTCTCTATAATACTTAGAGAATATGG

>941803_3_250

TGTAAGAATATCTGTTTTGAGCGGCTAATTACCAGTGGTAACGATCCCTATTACTTTTTGATGGATTTAGGGAAATTAATTTTTGGATCAATTTTCGCATTTTTTGTAAGGAGGAGGGTCATCAAAATTTGCAAAATATGGCCAAAAAATTTAATTTCCATTTTTGAACACAGTTTGATTGGAAACTTTATTACGAGCTCAGTGAGGTATGACATTCCATATTCAGACAATTATTTTTTATGTTGTGGCA

>942830_3_250

TGTCACATAGATGTAAGAATATCTGTTTTGAGCGGCTAATTACCAGTGCTAACGATCCCTATTACTTTTTGATGGATTTAGGGAAATTAATTTTTGGATCAATTTTCGCATTTTTTGTAAGGAGGGGGGTCATCAAAATTTGCAAAATATGGCCAAAAAATTTAATTTCCATTTTTGAACACAGTTTGATTGGAAACTTTATTACGAGCTCAGTGAAGTATGACATTCTATATTCTGACAAAAAAATTTT

>943952_3_249

TGTCGTAAAACAGCTAGCTTACACTACTATATCCATTCACTAAAATGGCTTTTCTCTATAATACTTAGAGAATATGGGAATATTTCAACATTTTTCCATATACATATAATAAATATTTATTTTCATATGTATATGATTTAATTTACTCATATAATTGCCAAAATCATATGAACACATACGTTTTGAACAATATGAGAGGTCGGCAACCACTGCCTACCTATAGTAGTTTTTGAACCCTCTGTCGTAAAA

>944074_3_249

TGTCTGAATATGGAATGTCATACCTCACTGAGCTCGTAATAAAATTTCCAATCAAACTGTGTTCAAAAATGGAAATGAAATTTTTTGGCCATATTTTGCAAATTTTGATGACCCCCCTCCTTACAAAAAATGCGAAAATTGATCCAAAAATTAATTTCCTAAATCCTTCAAAAAGTAATAGGGATCGTTAGCACTGGTAATTAGCAGCTCAAAACATCTTACATCTATGTGACCATTTTTAGCAAAGTT

>990156_3_249

TTTAATTTACTCATATAATTGCCAAAATCATATGAACACATACGTTTTGAACAATATGAGAGGTCGGCAACCACTGCCTTTTTCATATGTATATGATTTAATTTACTCATATAATTGCCAAAATCATATGAATACATACGTTTTGAACAATATGAGAGGTCGGCAACCACTGCCTACCTATTTTAGTAGTTAATGACGAGGTGTTTGGCTACTCTTGATAAAATTCTTTATATATTAATTATATTCTCT

>993259_2_250

AAAATGGCTTTTCTCTATAATACTTAGAGAATATGGGAATATTTCAACATTTTTCCATATACATATAATAAATATTTATTTTCATATGTATATGATTTAATTTACTCATATAATTGCCAAAATCATATGAACACATACGTTTTGAACAATATGAGAGGTCGGCAACCACTGCCTACCTATAGTAGTTTTTGAACCCTCTGTCGTAAAACAGCTAGCTTACACTACTATATCCATTCACTAAAATGGCTTT

>1025133_2_250

AACATAGAATAACATAGAATAACATAGAATAACATAGAATAACATAGAATAACATAGAATAACATAGAATAACATAGAATAACATAGAATAACATAGAATAACATAGAATAACATAGAATAACATAGAATAACATAGAATAACATAGAATAACATAGAATAACATAGAATAACATGGAATGGAATAGAATAGAAAGAATAGAATAGAAAAGAAAGAATAGAATAGAATAGAATAACACAGAATAACAGAG

>1040191_2_250

AACATAGAATAACATAGAATAACATAGAATAACATGGAATAGAAAGAATAGAATAGAATATAATAACATAGAATAACATAGAATAAAATAGAATAACATAGAATAACATAGAAAAACATAGAATAACATAGAATAACATAGAATAACATAGAATAACATAGAATAACATAGAATAACATAGAATAACATAGAATAACATAGAATAACATAGAATAACATAGAATAACATAGAATAACATAGAATAACATA

>1074683_2_250

AAGAAAAGAAAAGAAAAGAAAAGAAAAGAAAAGAAAAGAAAAGAAAAGAAAAGAAAAGAAAAGAAAAGAAAAGAAAAGAAAAGAAAAGAAAAGAAAAGAAAAGAAAAGAGAAGAGAAGAGAAGAGAAGAGAAGAGAAGAGAAGAGAAGAGAAGAGAAGAGAAGAGAAGAGAAGAGAAGAGAAGAGAAGAGAAGAGAAGAGAAGAGAAGAGAAGAGAAGAGAAGAGAAGAGAAGAGAAGAGAAGAGAAGAG

>1081078_2_250

AAGCTAGCTGTTCTACGACAGAGGGTTCAAAAACTACTATAGGTAGGCAGTGGTTGCCGACCTCTCATATTGTTCAAAACGTATGTGTTCATATGATTTTGGCAATTATATGAGTAAATTAAATCATATACATATGAAAATTAATATTTATTATGTGTATAAGTGAAAAATGTTGAAATATTCCCATATTCTCTAAGTATTATAGAGAAAAGCCATTTTAGTGAATGGATATAGTACTGTAAGCTAGCTG

>1108785_2_250

ACATAAAATAACAAAGAATAACATAGAATAACATAGAATAACATAGAATAACATAGAATAACATAGAATAACATAGAATATCACAGAATAACATAGAATAACATAGAATAACATAGAATAACATAGAATAACATAGAATAACATAGAATAACATAGAATAACATAGAATAACATAGAATAACATAGAATAACATAGAATAACAATATCACAGAATAACATAGAATAACATAGAATAAAATAGAATAACAA

>1178088_2_250

AGAAGAGAAGAGAAGAGAAGAGAAGAGAAGAGAAGAGAAGAGAAGAGAAGAGAAGAGAAGAGAAGAGAAGAGAAGAGAAGAGAAGAGAAGAGAAGAGAAGAGAAGAGAAGAGAAGAGAAGAGAAGAGAAGAGAAGAGAAGAGAAGAGAAGATAAGATAAGATAAGATAAGATAAGATAAGATAAGATAAGATAAGATAAGATAAGATAAGATAAGATAAGATAAGATAAGATAAGATAAGATAAGATAAG

>1181370_2_250

AGAATAACAAAGAATAACATAGAATAACATATAATAACATAGCATAACATAGAATAACATAGAATAACATAGAATAACATAGAATAACATAGAATAACATAGAATAACATAGAATAACATAGAATAACATAGAATAACATAGAATAACATAGAATAACATAGAATAACATAGAATAACATAGAATAACATAGAATAACATAGAATAACATAGAATAACAATAGCAAGTGTTGACAGCCGCGATCGACCAG

>1192268_2_250

AGAATAACATAGAATAACATAGAATAACATAGAATAACATAGAATAACATAGAATAACATAGAATAACATAGAATAACATAGAATAACATAGAATAACATAGAATTACATAAAATAACATACAATAACATAGAATAACAGAGAATAACATAGAATAACATAGAATAACAGAGAATAACAAAGAATAACATAGAATAACATAGAATAACATAGAATTACATAAAATAACATACAATAACATAGAATAACAG

>1211317_2_250

AGAATAACATGGAATAGAAAGAATAGAATAGAAAGAATAGAATAGAATATAATAACATAGAATAACATAGAATAACATAGAATAACATAGAATAACATAGAATAACATAGAATAACATAGAATAACATAGAATAACATAGAATAACATAGAATAACATAGAATAACATAGAATAACATAGAATAACATAGAATAACATAGAATAACATAGAATAACATAGAATAACATAGAATAACATAGAATAACATAG

>1241586_2_250

AGAGAAGAGAAGAGAAGAGAAGAGAAGAGAAGAGAAGAGAAGAGAAGAGAAGAGAAGAGAAGAGAAGAGAAGAGAAGAGAAGAGAAGAGAAGAGAAGAGAAGAGAAGAGAAGAGAAGAGAAGAGAAGAGAAGAGAAGAGAAGAGAAGAGAAGAGAAGACAAGACAAGACAAGACAAGACAAGACAAGACAAGACAAGACAAGACAAGACAAGACAAGACAAGACAAGACAAGACAAGACAAGACAAGACA

>1242108_2_250

AGAGAAGAGAAGAGAAGAGAAGAGAAGAGAAGAGAAGAGAAGAGAAGAGAAGAGAAGAGAAGAGAAGAGAAGAGAAGAGAAGAGAAGAGAAGAGAAGAGAAGAGAAGAGAAGAGAAGAGAAGAGAAGAGAAGAGAAGAGAAGAGAAGAGAAGAGAAGAGAGAAGAGAGAAGAGAGAAGAGAGAAGAGAGAAGAGAGAAGAGAGAAGAGAGAAGAGAGAAGAGAGAAGAGAGAAGAGAGAAGAGAGAAGAG

>1242395_2_248

AGAGAAGAGAAGAGAAGAGAAGAGAAGAGAAGAGAAGAGAAGAGAAGAGAAGAGAAGAGAAGAGAGAGAAGAGAAGGGGAAAGGTACTGTGTTGTGTTGTGTTGTGTTGTGTTGTGTTGTGTTGTGTTGTGTTGTGTTGTGTTGTGTTGTGTTGTGTTGTGTTGTGTTGTGTTGTGTTGTGTTGTGTTGTGTTGTGTTGTGTTGTGTTGTGTTGTGTTGTGTTGTGTTGTGTTGTGTTGTGTTGTGTT

>1263584_2_250

AGGAGAGGAGAGGAGAGGAGAGGAGAGGAGAGGAGAGGAGAGGAGAGGAGAGGAGAGAAGAGAAGAGAAGAGAAGAGAAGAGAAGAGAAGAGAAGAGAAGAGAAGAGAAGAGAAGAGAAGAGAAGAGAAGAGAAGAGAAGAGAAGAGAAGAGAAGAGAAGAGAAGAGAAGAGAAGAGAAGAGAAGAGAAGAGAAGAGAAGAGAAGAGAAGAGAAGAGAAGAGAAGAGAAGAGAAGAGAAGAGAAGAGAAG

>1265531_2_250

AGGATTGGTCCAAGTACCGATCCTTGGGGAAGAGAAGAGAAGAGAAGAGAAGAGAAGAGAAGAGAAGAGAAGAGAAGAGAAGAGAAGAGAAGAGAAGAGAAGAGAAGAGAAGAGAAGAGAAGAGAAGAGAAGAGAAGAGAAGAGAAGAGAAGAGAAGAGAAGAGAAGAGAAGAGAAGAGAAGAGAAGAGAAGAGAAGAGAAGAGAAGAGAAGAGAAGAGAAGAGAAGAGAAGAGAAGAGAAGAGAAGAGG

>1266055_2_235

AGGCAAGGCAAGGCAAGGCAAGGCAAGGCAAGGCAAGGCAAGGCAAGGCAAGGCAAGGCAAGACAAGACAAGACAAGACAAGACAAGACAAGACAAGACAAGACAAGACAAGACAAGACAAGACAAGACAAGACAAGACAAGACAAGACAAGACAAGACAAGACAAGACAAGACAAGACAAGACAAGACAAGACAAGACAAGACAAGACAAGACAAGACAAGACAAGACAAGACA

>1281269_2_250

AGTGTCTGAACTTAACCAATAAATGAAAATTAACAGTAACACTGGCGGTTTTATTTATAAACAATAGAATAACATAGAATAACATAGAATAACATAGAATAACATAGAATAACATAGAATAACATAGAATAACATAGAATAACATAGAATAACATAGAATAACATAGAATAACATAGAATAACATAGAATAACATAGAATAACATAGAATAACATAGAATAACATAGAATAACATAGAATAACATAGAAA

>1295025_2_249

ATAACATAGAATAACATAGAATAACATAGAATAACATAGAATAACATAGAATAACATAGAATAACATAGAATAACATAGAATAACATAGAATAACATAGAATAACATAGAATAACATAGAATAACATAGAATAACATGGAATAGAAAGAATAGAATAGAATATAATAACATAGAATAACATAGAATAACATAGAATAAAATAGAATAACATAGAATAACATAGAAAAACATAGAATAACATAGAATAAC

>1316798_2_215

ATAATATAATATAATATAATATAATATAATATAATATAATATAATATAATATAATATAATATAATATAATATAATATAATATAATATAATATAATATAATATTATATTATATTATATTATATTATATTATATTATATTATATTATATTATATTATATTATATTATATTATATTATATTATATTATATTATATTATATTATATTATATTATATTAT

>1450162_2_250

TAACAAAGAATAACAAAGAACATAGAATAACGTAGAATAACATAGAATAACATAGAATAACATATAATAACATAGATTTACATAGAATAACATAGAATAACATAGAATAACATAGAATAACATAGAATAACATAGAATAACATAGAATAACATAGAATAACATAGAATAACATAGAATAACATAGAATAACATAGAATAACATAGAATAACATAGAATAACATAGAATAACATAGAATAACATAGAATAA

>1544275_2_250

TAGAATAACATAGAATAACATAGAATAACATAGAATAACATAGAATAACATAGAATAACATAGAATAACATAGAATAACATAGAATTACATAAAATAACATACAATAACATAGAATAACAGAGAATAACATAGAATAACATAGAATAACAGAGAATAACAAAGAATAACATAGAATAACATAGAATAACATAGAATTACATAAAATAACATACAATAACATAGAATAACAGAGAATAACATAGAATAACA

>1547393_2_250

TAGAATAACATAGAATAACATAGAATAACATAGAATAACATAGAATAGCATAGAATAGCATAGAATACCATATAATAACATAGAATAACGTAGAATAACATAGAATAACATAGAATAACATAGAATAACAGAGAATAACAAAGAATAACATAGAATAACATAGAATAACATAGAATAACATAGAATAACATAGAATAACATAGAATAACATAGAATAACATAGAATAACATAGAATAACAGAGAATAACA

>1548432_2_249

TAGAATAACATAGAATAACATAGAATAACATAGAATAACATGGAATAACATGGAATAGAAAGAGTAGAATAGAATATAATAACATAGAATAACATAGAATAACATAGAATAACATAGAATAACATAGAATAACATAGAATAACATAGAATAACATAGAATAACATAGAATAACATAGAATAACATAGAATAACATAGAATAACATAGAATAACATAGAATAACATAGAATAACATAGAATAACATAGAA

>1571516_2_250

TAGACTAACATAAAATAACATAAAATAACATAGAATAACATAGAATAACACAGAATAACATAGAATAACATAGAATAACATAGAATAACATAGAATAACATAGAATAACATAGAATAACATAGAATAACATAGAATAACATAGAATAACATAGAATAACATAGAATAACATAGAATAACATAGAATAACATAGAATAACATAGAATATAACAGAATAACATAGAATAACATAGAATGACATAGAAGAACA

>1579022_2_250

TAGCTGCTCAAAACATCTTACATCTATGTGACCATTTTTAGCCAAGTTATAACGAAAATTTCGTTTGTAAATATCAACATTTTTGCAGAGTCTGTTTTTCCAAATTTCGGTCATCAAATAATCATTTATTTTGCCACAACATAAAAAATAATTGTCTGAATATGGAATGTCATACCTCACTGAGCTCGTAATAAAATTTCCAATTAAACTGTGTTCAAAAATGGAAATTAAATTTTTTGGCCATATTTTG

>1683553_2_250

TGAACACATACGTTTTGAACAATATGAGAGGTCGGCAACCACTGCCTTTTTCATATGTATATGATTTAATTTACTCATATAATTGCCAAAATCATATGAATACATACGTTTTGAACAATATGAGAGGTCGGCAACCACTGCCTACCTATTATAGTAGTTAATGACGAGGTGTTTGGCTACTCTTGATAAAATTCTTTAAATTCTTTATATATTAATTATATGATAGGGACAATATCATACGCGTCACTAA

>1694727_2_250

TGAGGTATGACATTCCATATTCAGACAATTATTTTTTATGTTGTGGCAAAATAAATGATTTTTTGATGACCAAAATTTGGAAAAACAGACTCTGCAAAAATGTTGATATTTACAAACGAAATTTTCGTTATAACTTGGCTAAAAATGGTCACATAGATGTACGAATATCTGTTTTGAGCAGCTAATTACCAGTGCTAACGATCCCTATTACTTTTTGAAGGATTAAGGAAATTCATTTTTGGGTCAATTT

>1700210_2_250

TGATTTTGGCAATTATATGAGTAAATTAAATCATATACATATGAAAAAGGCAGTGGTTGCCGACCTCTCATATTGTTAAAAACGTATGTGTTCATATGATTTTGGCAATTATATGAGTAAATTAAATCATATACATATGAAAATTAATATTTATTATGTGTATAAGTGAAAAATGTTGAAATATTCCCATATTCTCTAAGTATTATAGAGAAAAGCCATTTTAGTGAATGGATATAGTAGTGTAAGCTAG

>1703862_2_250

TGCCACAACATAAAAAATAATTGTCTGAATATGGAATGTCATACCTCACTAAGCTCGTAATAAAATTTCCAATCAAACTGTGTTCAAAAATGGAAATGAAATTTTTTGGCCATATTTTGCAAATTTTGATGACCCCCCTCCTTACAAAAAATGCGAAAATTGATCCAAAAATTAATTTCCTAAATCCTTCAAAAAGTAATAGGGATCGTTAGCACTGGTAATTAGCAGCTCAAAACATCTTACATCTATG

>1724311_2_249

TGGCCATAAAATTTAATTTCCATTTTTGAATACAGTTTGATTGGAAATTTTCTTACGAGCTTAGTGAGGTATGACATTCCATATTCAGACAATTATTTCGTATGTTGTGGCAAAATAAATGATTATTTGATGACCGAAATTTGGAAAAACAGACTCTGCAAAAATGTTGATATTTACAAACGAAATTTTCGTTATAACTTGGCTAAAAATGGTCACATAGATGTAAGAATAACTGTTTTGAGCAGCTAA

>1725327_2_250

TGGCTAAAAATTGTCACAGACTCTGCAAAAATGTTGATATTTACAAACGAAATTTTCGTTATAACTTGGCTAAAAATGGTCACATAGATGTAAGAATAACTGTTTTGAGCAGCTAATTACCAGTGCTAACGATCCCTATTACTTTTTGAAGGATTTAGGGAAATTAATTTTTGGATCAATTTTCGCATTTTTTGTAAGGAGGGGGGTCATCAAAATTTGCCAAATATGGCCAAAAAATTTAATTTCCATT

>1844183_2_250

TTCTCTATAATACTTAGAGAATATGGGAATATTTCAACATTTTTCACTTATACACATAATAAATATTAATTTTCATATGTATATGATTTAATTTACTCATATAATTGCCAAAATCATATGAACACATACGTTTTGAACAATATGAGAGGTCGGCAACCACTGCCTACCTATAGTAGTTTTTGAACCCTCTGTCGTAGAACAGCTAGCTTACAGTACTATATCCATTCACTAAAATGGCTTTTCTCTATAA
