# Supplementary material for: Simple and Complex Centromeric Satellites in Drosophila Sibling Species
Source: Genetics. 2018 Jan 5;208(3):977–90. doi: 10.1534/genetics.117.300620 (PMC5844345; doi:10.1534/genetics.117.300620)
Supplement: Supplementary file 6 [file 977FileS2.docx]

**File S2. Top 100 IP reference sequences that map IP reads from S2 cells.** The sequence names have the form: Reference number_number of reads contributing to the cluster_length of sequence.

>10_8059_250

AGAGAAGAGAAGAGAAGAGAAGAGAAGAGAAGAGAAGAGAAGAGAAGAGAAGAGAAGAGAAGAGAAGAGAAGAGAAGAGAAGAGAAGAGAAGAGAAGAGAAGAGAAGAGAAGAGAAGAGAAGAGAAGAGAAGAGAAGAGAAGAGAAGAGAAGAGAAGAGAAGAGAAGAGAAGAGAAGAGAAGAGAAGAGAAGAGAAGAGAAGAGAAGAGAAGAGAAGAGAAGAGAAGAGAAGAGAAGAGAAGAGAAGAGG

>124_1151_250

TAACATAGAATAACATAGAATAACATAGAATAACATAGAATAACATAGAATAACATAGAATAACATAGAATAACATAGAATAACATAGAATAACATAGAATAACATAGAATAACATAGAATAACATAGAATAACATAGAATAACATAGAATAACATAGAATAACATAGAATAACATAGAATAACATAGAATAACATAGAATAACATAGAATAACATAGAATAACATAGAATAACATAGAATAACATAGAA

>1135_168_250

TAGAATAGAATAGAATAGAATAGAATAGAATAGAATAGAATAGAATAGAATAGAATAGAATAGAATAGAATAGAATAGAATAGAATAGAATAGAATAGAATAGAATAGAATAGAATAGAATAGAATAGAATAGAATAGAATAGAATAGAATAGAATAGAATAGAATAGAATAGAATAGAATAGAATAGAATAGAATAGAATAGAATAGAATAGAATAGAATAGAATAGAATAGAATAGAATAGAATAGAA

>1384_149_250

AGATAAGATAAGATAAGATAAGATAAGATAAGATAAGATAAGATAAGATAAGATAAGATAAGATAAGATAAGATAAGATAAGATAAGATAAGATAAGATAAGATAAGATAAGATAAGATAAGATAAGATAAGATAAGATAAGATAAGATAAGATAAGATAAGATAAGATAAGATAAGATAAGATAAGATAAGATAAGATAAGATAAGATAAGATAAGATAAGATAAGATAAGATAAGATAAGATAAGATA

>4220_83_250

AACATAGAATAACATAGAATAACATAGAATAACATAGAATAACATTGAATAACATTGAATAACATAGCATAACATAGAAAAGCATAGAATAACAAAGAATAACATAGAATAACATATAATAACATAGAATAACATAGAATAACATAGAATAACATATAATAACATAGAATAACATATAATAACATAGAATAGCATAGAATAACATAGAATAACATAGAATAACATTGAATAAGATAGCATAACATAGAAA

>5851_71_250

TGGATAACATAGAATAACATAGCATAACATAGAAAAGCATAGAATAACAAAGAATAACATAGAATAACATATAATAACATAGCATAACATAGAATAACATAGAATAACATATAATAACATAGAATAACAAGAATAACATAGAATAACATAGAATAACATAGAATAACATAGAATAACATAGAATAACATAGAATAACATAGAATAACATAGAATAACATAGAATAACATAGAATAACATAGAATAACATA

>7126_64_250

TAACAAAGAATAACATAGAATAACATATAATAACATAGAATAACATAGAATAACATAGAATAACATATAATAACATAGAATAACATATAATAACATAGAATAGCATAGAATAACATAGAATAACATAGAATAACATTGAATAAGATAGCATAACATAGAAAAGCATAGAATAACAAAGAATAACATAGAATAACATATAATAACATAGCATAACATAGAATAACATAGAATAACATAGAATAACATAGAA

>8328_59_250

TAACATAGAAAAGCATAGAATAACAAAGAATAACATAGAATAACATATAATAACATAGAATAACATAGAATAACATAGAATAACATATAATAACATAGAATAACATATAATAACATAGAATAGCATAGAATAACATAGAATAACATAGAATAACATTGAATAAGATAGCATAACATAGAAAAGCATAGAATAACAAAGAATAACATAGAATAACATATAATAACATAGCATAACATAGAATAACATAGAA

>9150_56_250

AGGAAAGCATAGAATAACAAAGAATAACATAGAATAACATATAATAACATAGAATAACATAGAATAACATAGAATAACATAGAATAACATAGAATAACATAGAATAACATAGAATAACATAGAATAACATAGAATAATATAGAATAACATAGAATAACATAGCATAACATAGAAACAAAGAATAACATTGAATAACATAGAAAAACATAGAATAACATGGAATAACATAGAGTAACATAGAATAACATAG

>10498_52_250

AGAATAACAATATCACAGAATAACATAGAATAACATAGAATAAAATAGAATAACATAGAATATCACAGAATAACATAGAATATCACAGAATAACATAGAATAACATAGAATAACGTAGAATAACATAGAATAACATAGAATAAAATAGAATAACATAGAATAACATAGAATAACATAGAATAACATAGAATAACATAGAATAACATAGAATAACATAGAATAACATAGAATAACATAGAATAACATAGAA

>19499_37_250

AGCATAACATAGAAAAGCATAGAATAACAAAGAATAACATAGAATAACATATAATAACATAGCATAACATAGAATAACATAGAATAACATATAATAACATAGAATAACAAGAATAACATAGAATAACATAGAATAACATAGAATAACATAGAATAACATAGAATAACATAGAATAACATAGAATAACATAGAATAACATAGAATAACATAGAATAACATAGAATAACATAGAATAACATAGAATAACATA

>21951_35_250

TAACATTGAATAACATAGCATAACATAGAAAAGCATAGAATAACAAAGAATAACATAGAATAACATATAATAACATAGCATAACATAGAATAACATAGAATAACATAGAATAACATAGAATAACATATAATAACATAGAATAACATATAATAACATAGAATAACATAGAATAACATAGAATAACATACAATAACATTGGATAACATAGAATAACATAGCATAACATAGAAAAGCATAGAATAACAAAGAA

>21953_35_250

TAACATTGAATAACATTGAATAACATAGCATAACATAGAAAAGCATAGAATAACAAAGAATAACATAGAATAACATATAATAACATAGAATAACATAGAATAACATAGAATAACATATAATAACATAGAATAACATATAATAACATAGAATAGCATAGAATAACATAGAATAACATAGAATAACATTGAATAAGATAGCATAACATAGAAAAGCATAGAATAACAAAGAATAACATAGAATAACATATAA

>21984_35_250

TAGAATAGAATATAATAGAAAAGAATATAATATAATACAATAGAATATAATGGGTTACAAGGAACAGAATAGAATATATAGAATAGAATAGAATATAGTAGAATAGAATAGAATATAATAGAATAGAATAGAATAGAAAAGAATAGAATAGAATGGAATATAGAGCATTATAGAATGGAATATAATAGAATGGAATATAATAGAATATAATATAATAGAATATAATATAATAGAATGGAATATAATAGAA

>23084_34_250

TAACATAGAATAACATAGAATAACATTGAATAACATTGAATAACATAGCATAACATAGAAAAGCATAGAATAACAAAGAATAACATAGAATAACATATAATAACATAGAATAACATAGAATAACATAGAATAACATATAATAACATAGAATAACATATAATAACATAGAATAGCATAGAATAACATAGAATAACATAGAATAACATTGAATAAGATAGCATAACATAGAAAAGCATAGAATAACAAAGAA

>24356_33_250

TAGAATAGAATAGAATAGAATAGAATAGAATAGAATAGAATATAATAGAAAAGAATATAATATAATACAATAGAATATAATGGGTTACAAGGAACAGAATAGAATATATAGAATAGAATAGAATATAGTAGAATAGAATAGAATATAATAGAATAGAATAGAATAGAAAAGAATAGAATAGAATGGAATATAGAGCATTATAGAATGGAATATAATAGAATGGAATATAATAGAATATAATATAATAGAA

>26418_31_250

ACATAGAATAACATAGAATAACATAGAATAACATATAATAACATAGAATAACATATAATAACATAGAATAACATAGAATAACATAGAATAACATAGAATAACATTGAATAACATTGAATAACATAGCATAACATAGAAAAGCATAGAATAACAAAGAATAACATAGAATAACATATAATAACATAGAATAACATAGAATAACATAGAATAACATATAATAACATAGAATAACATATAATAACATAGAATA

>33462_27_250

AGAAAAGAATATAATATAATACAATAGAATATAATGGGTTACAAGGAACAGAATAGAATATATAGAATAGAATAGAATATAGTAGAATAGAATAGAATATAATAGAATAGAATAGAATAGAAAAGAATAGAATAGAATGGAATATAGAGCATTATAGAATGGAATATAATAGAATGGAATATAATAGAATATAATATAATAGAATATAATATAATAGAATGGAATATAATAGAATAAATAGAATAGAATA

>36149_26_250

ATAACATATAATAACATAGCATAACATAGAATAACATAGAATAACATATAATAACATAGAATAACAAGAATAACATAGAATAACATAGAATAACATAGAATAACATAGAATAACATAGAATAACATAGAATAACATAGAATAACATAGAATAACATAGAATAACATAGAATAACATAGAATAACATAGAATAACATAGAATAACATAGAATAACATAGAATAACATAGAATAACATAGAATAACATAGAA

>38042_25_250

ACATAGAATAACATATAATAACATAGAATAACATATAATAACATAGAATAACATAGAATAACATAGAATAACATAGAATAACATTGAATAACATTGAATAACATAGCATAACATAGAAAAGCATAGAATAACAAAGAATAACATAGAATAACATATAATAACATAGAATAACATAGAATAACATAGAATAACATATAATAACATAGAATAACATATAATAACATAGAATAGCATAGAATAACATAGAATA

>47735_22_250

ATATTATATTATATTATATTATATTATATTATATTATATTATATTATATTATATTATATTATATTATATTATATTATATTATATTATATTATATTATATTATATTATATTATATTATATTATATTATATTATATTATATTATATTATATTATATTATATTATATTATATTATATTATATTATATTATATTATATTATATTATATTATATTATATTATATTATATTATATTATATTATATTATATTATATT

>52405_21_250 359bp?

TACAAGCTCAGTGAGGTATGACATTCCATATTCAGACAATTATTTTTTATGTTGTGGCAAAATAAATCATTATTTGATGACCGAAATTTGGAAAAACAGATTCTGCCAAAATGTTGATATTTACAAACGAAATTTTCGTTATAACTTGGCTAAAAATGGTCACATAGATCTAAGAATAACTGTTTTGAGCAGCTAATTACCAGTGCTAACGATCCCTATTACTTTTTGAAGGATTAAGGGAAATTAATTT

>54137_20_250

AACATAGAATAACATAGAATAACATAGAATAACATAGAATAACATAGAATAACATAGAATAACATAGAATAACATAGCATAACATAGAAACAAAGAATAACATTGAATAACATAGAACAACATAGAATAACATGGAATAACATAGAGTAACATAGAATAACATAGCATAACACAGAATAACATAGAATAACATAGAATAACATAGAATAACATTGGATAACATAAAAAAACATAGAATAACATAGAATAA

>55034_20_250

AGAATAACATAGAATAACATAGAATAACATAGAATAACATAGAATAACATAGAATAACATAGAATAACATAGAATAACATAGAATAACATAGCATAACATAGAAACAAAGAATAACATTGAATAACATAGAACAACATAGAATAACATGGAATAACATAGAGTAACATAGAATAACATAGCATAACACAGAATAACATAGAATAACATAGAATAACATAGAATAACATTGGATAACATAAAAAAACATAG

>61801_19_250

TAGAATAGAATAGAATAGAATAGAATATAATAGAAAAGAATATAATATAATACAATAGAATATAATGGGTTACAAAGAACAGAATAGAATATATAGAATAGAATAGAATATAGTAGAATAGAATAGAATATAATAGAATAGAATAGAATAGAAAAGAATAGAATAGAATGGAATATAGAGCATTATAGAATGGAATATAATAGAATGGAATATAATAGAATATAATATAATAGAATATAATATAATAGAA

>63568_18_250

AAAGCATAGAATAACAAAGAATAACATAGAATAACATATAATAACATAGCATAACATAGAATAACATAGAATAACATATAATAACATAGAATAACAAGAATAACATAGAATAACATAGAATAACATAGAATAACATAGAATAACATAGAATAACATAGAATAACATAGAATAACATAGAATAACATAGAATAACATAGAATAACATAGAATAACATAGAATAACATAGAATAACATAGAATAACATAGAA

>64691_18_250

ACATAGAATAACATATAATAACATAGAATAACATAGAATAACATAGAATAACATAGAATAACATTGAATAACATTGAATAACATAGCATAACATAGAAAAGCATAGAATAACAAAGAATAACATAGAATAACATATAATAACATAGAATAACATAGAATAACATAGAATAACATATAATAACATAGAATAACATATAATAACATAGAATAGCATAGAATAACATAGAATAACATAGAATAACATTGAATA

>65385_18_250

AGACAAGACAAGACAAGACAAGACAAGACAAGACAAGACAAGACAAGACAAGACAAGACAAGACAAGACAAGACAAGACAAGACAAGACAAGACAAGACAAGACAAGACAAGACAAGACAAGACAAGACAAGACAAGACAAGACAAGACAAGACAAGACAAGACAAGACAAGACAAGACAAGACAAGACAAGACAAGACAAGACAAGACAAGACAAGACAAGACAAGACAAGACAAGACAAGACAAGACA

>69824_17_250

AACATAGAATAACATAGAATAACATAGAATAACATAGAATAACATAAAATAACATAGAATAACATAGAATAACATAGAATAACATAGAATAACATAGAATAACATAGAATAACATAGAATAATATAGAATAACATAGAATAACATAGCATAACATAGAAACAAAGAATAACATTGAATAACATAGAAAAACATAGAATAACATGGAATAACATAGAGTAACATAGAATAACATAGCATAACACAGAATAA

>70616_17_250

ACATAGAATAACATAGAACAACATAGAATAACATGGAATAACATAGAGTAACATAGAATAACATAGAATAACGTAGCAGAACACAGAATAACATAGAATAAAATAGCATAGCATAGAATAACAAAGAATAACATAGCATAACATAGAATAACATAGAATAACATAGAATAACATAGAATAACATAGAATAACATAGAATAACATAGAATAACATAGAATAACATAGAATAACATAGAATAACATAGAATA

>71135_17_250

AGAATAAAATAGAATAACATAGAATAACATAGAATAACATAGAATAACATAGAATAACATAGAATAACATAGAATAACATAGAATAACATAGAATAACATAGAATAACATAGAATAACATAGAATGACATAGAATAACATAGAATAACATAGAATAACATAGAATAACATAGAATATCACAGAATAACATAGAATAACATAGAATAACAAAGAATAACATAAAATAACAAAGAATAACAAAGAATAACAA

>71263_17_250

AGAATAACATAGAATAACATAGAATAACATAGAATAACATAGAATAACATAGAATAACATAGAATAACATAGAATAACATCGAATAACATAGAATAACATAGAATAACATAGAATAACATAGAATAAAATAGAATAACATAGAATGACATAGAATAACATAGAATAACATAAAATAACATAGAATAACATAGAATATCGCAGAATAACATAGAATAACATAGAATAACAAAGAATAACATAAAATAACAA

>81395_16_250

TAGAATAACATAGAATAACATAGAATAACATAGAATAACATAGAATAACATAGAATAACATAGAATAACATAGAATAACATAGAATAACATAGAATAACATAGAATAACATAGAGTAACATAGAATAACATAGAATAACGTAGCAGAACACAGAATAACATAGAATAACATAGAATAAAATAGCATAACATAGAATAACAAAGAATAACATAGCATAACATAGAATAACATAGAATAACATAGAATAACA

>87342_15_250

AGCATAACATAGGAAAGCATAGAATAACAAAGAATAACATAGAATAACATATAATAACATAGAATAACATAGAATAACATAGAATAACATAGAATAACATAGAATAACATAGAATAACATAGAATAACATAGAATAACATAGAATAACATAGAATAACATAGAATAACATAGAATAATATAGAATAACATAGAATAACATAGCATAACATAGAAACAAAGAATAACATTGAATAACATAGAAAAACATAG

>95104_14_250

ACATAGAATAACATACAATAACATTGGATAACATAGAATAACATAGCATAACATAGAAAAGCATAGAATAACAAAGAATAACATAGAATAACATATAATAACATAGCATAACATAGAATAACATAGAATAACATATAATAACATAGAATAACAAGAATAACATAGAATAACATAGAATAACATAGAATAACATAGAATAACATAGAATAACATAGAATAACATAGAATAACATAGAATAACATAGAATAA

>99233_14_250

TAACATAAAAAAACATAGAATAATATAGAATAGCATAGAATAATATAGAATAATATAGAATAACATAGAATAACATAGAACAACATAGAATAACATGGAATAACATAGAGTAACATAGAATAACATAGAATAACGTAGCAGAACACAGAATAACATAGAATAACATAGAATAAAATAGCATAACATAGAATAACAAAGAATAACATAGCATAACATAGAATAACATAGAATAACATAGAATAACATAGAA

>107464_13_250

AGAATAACATAGAATAACATAGAATAACATACAATAACATTGGATAACATAGAATAACATAGCATAACATAGAAAAGCATAGAATAACAAAGAATAACATAGAATAACATATAATAACATAGCATAACATAGAATAACATAGAATAACATATAATAACATAGAATAACAAGAATAACATAGAATAACATAGAATAACATAGAATAACATAGAATAACATAGAATAACATAGAATAACATAGAATAACATA

>108468_13_250

AGAGTAACATAGAATAACATAGCATAACACAGAATAACATAGAATAACATAGAATAAAATAGAATAACATAGAGTAACATAGAATAACATAGAATAACATAGAATAACATAGAATAACATAGAATAACATAGAATAACATAGAATAAGATAGAATAACATAGAATAACATAGCATAACATAGAAACAAAGAATAACATTGAATAACATAGAACAACATAGAATAACATGGAATAACATAGAGTAACATAG

>112293_13_250

TAACATCGAATAACATAGAATAACATAGAATAACATAGAATAACATAGAATAAAATAGAATAACATAGAATGACATAGAATAACATAGAATAACATAAAATAACATAGAATAACATAGAATATCGCAGAATAACATAGAATAACATAGAATAACAAAGAATAACATAAAATAACAAAGAATAACAAAGAATAACAAAGAATAACATAGAATAACATAGAATAACATAGAATAACATAAAATAACATAGAA

>119378_12_250

ACACAGCATAACATAGAATAACATAGAATAACAAGAATAACATAGAATAACATAGAATAACATAGAATAACATAGAATAACATAGAATAACATAGAATAACATAGAATAACATAGAATAACATAGAATAACATAGAATAACATAGAATAACATAGAATAACATAGAATAACATAGAATAACATAGAATAACATAGAATAATATAGAATAACATAGAATAACATAGCATAACATAGAAACAAAGAATAACA

>145741_11_250

TAGCATAACATAGAATAACATAGAATAACATAGAATAACATAGAATAACATAGAATAACATAGAATAACATAGAATAACATAGAATAACATAGAATAACATAGAATAACATAGAATAACATAGAATAACATAGAGTAACATAGAATAACATAGAATAACGTAGCAGAACACAGAATAACATAGAATAACATAGAATAAAATAGCATAACATAGAATAACAAAGAATAACATAGCATAACATAGAATAACA

>147343_11_249

TGAAAATTAACAGTAACACTGGCGGTTTTATTTATAAACAATAGAATAACATAGAATAACATAGAATAACATAGAATAACATAGAATAACATAGAATAACATAGAATAACATAGAATAACATAGAATAACATAGAATAACATAGAATAACATAGAATAACATAGAATAACATAGAATAACATAGAATAACATAGAATAACATAGAATAACATAGAATAACATAGAATAACATAGAATAACATAGAATAA

>167079_10_250

TAGAATAACATAGAATAACATAGAATATCACAGAATAACATAGACTAACATAGAATAACATAGAATAACATAAAATAACAAAGAATAACAAAGAATAACAAAGAATAACATATAATAACATAGATTTACATAGAATAACATAGAATAACATAGAATAACATAGAATAACATAGAATAACATAGAATAACATAGAGAAACATAGAATAACATAGAATAACATAGAATAACATAGAATAACATAGAATAACA

>180798_9_250

ACATAGAATAACATATAATAAAGTAGAATAAAGTAGAATGACATAGAATAACATAGAATAACATAGCATAACACAGAATAACATAGAATAACATAGAATAAAATAGCATAACATAGAATAACAAAGAATAACATAGCATAACATAGAATAACATAGAATAACATAGAATAACATAGAATAACATAGAATAACATAGAATAACATAGAATAACATAGAATAACATAGAATAACATAGAATAACATAGAATA

>180996_9_250

ACATAGAATAGCATAGAATAACATAGAATAACATAGCATAACATAGAATAACATAGAATAACATAGAATAACATAGAATAACATAGAATAACATAGAATAACATAGAATAACATAGAATAACATAGAATAACATAGAATAACATAGAGTAACATAGAATAACATAGAATAACGTAGCAGAACACAGAATAACATAGAATAACATAGAATAAAATAGCATAACATAGAATAACAAAGAATAACATAGCATA

>194353_9_250

TAGAATAACATAGAATAACATAGAATAACATAGAATAACATAGAATAACATAGAATAACATAGAATAACATAGAATAACATAGAATAACATAGAATAACATAGAATAACATATAATAACATAGAATAACATAGAATAACATATAATAACATAGAATAACAAAGAATAACATAGAATAACATTGAATAACATTGAATAACATAGGATAACATAGAAAAACATATAATAACATATAATAACATAGAATAACA

>208851_8_250

AACATTGAATAACATAGCATAACATAGGAAAGCATAGAATAACAAAGAATAACATAGAATAACATATAATAACATAGAATAACATAGAATAACATAGAATAACATAGAATAACATAGAATAACATAGAATAACATAGAATAACATAGAATAACATAGAATAACATAGAATAACATAGAATAACATAGAATAATATAGAATAACATAGAATAACATAGCATAACATAGAAACAAAGAATAACATTGAATAA

>210374_8_250

ACATAAAATATCACAGAATAACATAGAATATCACAGAATAACATCGAATAACATCGAATAACATAGAATATCACAGAATAACATAGACTAACATGGAATAACATAGAATAACATAAAATAACATAGAATAACATAGAATAACATAGAATAACATAGAATAACATAGAATAACATAGAATAACATAGAATAACATAGAATAACATAGAATAACATAGAATAACATAGAATAACATAGAATAACATAGAATA

>210851_8_250

ACATAGAATAACATAGAATAACATAGAATAACATAGAATAACATAGAATAACATAGAATAACATAGAATAAAATAGAATAACATAGAATGACATAGAATAACATAGAATAACATAAAATAACATAGAATAACATAGAATATCGCAGAATAACATAGAATAACATAGAATAACAAAGAATAACATAAAATAACAAAGAATAACAAAGAATAACAAAGAATAACATAGAATAACATAGAATAACATAGAATA

>227558_8_250

TAACATAGAATAACATAGAATAACATTGAATAAGATAGCATAACATAGAAAAGCATAGAATAACAAAGAATAACATAGAATAACATAGAATAACATGGAATAACATAGAGTAACATAGAATAACATAGAATAACGTAGAAGAACACAGAATAACATAGAATAACATAGAATAAAATAGCATAACATAGAATAACAAAGAATAACATAGCATAACATAGAATAACATAGAATAACATAGAATAACATAGAA

>253132_7_250

ACATAGAATAACATAGAGAAACATAGAATAACATAGAATAACATAGAATAACATAGAATAACATAGAATAACATAGAATAACATAGAATGACATAGAATAACATAGAATAACATAGAATAACATAGAATATCACAGAATAACATAGAATAACATAGAATAACAAAGAATAACATAAAATAACAAAGAATAACAAAGAATAACAAAGAATAACATAGAATAACATAGAATAACATAGAATAACATAAAATA

>272278_7_250

TAACATAGAATAACATAGAATAACATAGAATAACATAGAATAACATAGAAAAGCATAGAATAACAAAGAATAACATAGAATAACATATAATAACATAGAATAACATAGAATAACATAGAATAACATATAATAACATAGAATAACATATAATAACATAGAATAGCATAGAATAACATAGAATAACATAGAATAACATTGAATAAGATAGCATAACATAGAAAAGCATAGAATAACAAAGAATAACATAGAA

>279215_7_250

TAGATTTACATAAAATTACATAGAAAAACATAGAATAACATAGAATAACATAGAATAACGTAGAATAACATAGAATAACATAGAATAACATATAATAACATAGATTTACATAGAATAACATAGAATAACATAGAATAACATAGAATAACATAGAATAACATAGAATAACATAGAATAACATAGAATAACATAGAATAACATAGAATAACATAGAATAACATAGAATAACATAGAATAACATAGAATAACA

>309497_6_250

ACATAGAATATCACAGAATAACATAGAAGAACATAGAATAACATAGAATAACATATAATAACATCGATTTACATAGAATAACATAGAATAACATAGAATAACATAGAATAACATAGAGAAACATAGAATAACATAGCATAACATAGAATAACATAGAATAACATAGAATAACATAGAATAACATAGAATAACATAGAATAAAATAGAATAACATAGAATAACATAGAATAACATAGAATAACATAGAATA

>390507_5_250

ACATCGAATAACATAGAATATCACAGAATAACATAGACTCACATAGAATAAAATAGAATAACATAAAATAACATAGAATAACATAGAATAACATAGAATAACATAGAATAACATAGAATAACATAGAATAACATAGAATAACATAGAATAACATAGAATAACATAGAATAACATAGAATAACATAGAATAACATAGAATATCACAGAATAACATAGACTAACATAGAATAACATAGAATAACATAAAATA

>394085_5_249

AGAATAACATAGAATAACAAAGAATAACATAAAATAACAAAGAATAACAAAGAATAACATAGAATAACATAGAATAACATAGAATAACATAAAATAACATAGAATAACATAGAATAACATAGAATAACATAGAATAACATAGAATAACATAGAATAACATAGAATAACATAGAATAACATAGAATAACATAGAATAACATAGAATAACATCGAATAACATCGAATAACATAGAATATCACAGAATAACA

>438359_5_250

TAGAATAACATAGAATAACATAGAATAAAATAGAATAACATAGAATAACATAGAATAACATAGAATAACATAGAATAACATAGAATAACATAGAATATCACAGAATAACATAGAATATCACAGAATAACATAGAATAACATAGAATAACATAGAATAACGTAGAATAACATAGAATAACAAAGAATAACATAGAATAACATAGAATAACATAGAATAACATAGAATAACGTAGAATAACATAGAATAACA

>506398_4_249

ACATAGAATAACATAGAATAACATAGAATAACATAGAATAACATAGAATAACATAGAATAACATGGAATAACATAGAATAACATAGAATAACATAGAATAACATAGAATAACATAGAATAACATAGAATAACATAGAATAACATAGAATAACATAGAATAACAATATCACAGAATAACATAGAATAACATAGAATAAAATAGAATAACATAGAATATCACAGAATAACATAGAATATCACAGAATAACA

>518165_4_250

AGAATAACATAAAATAACAAAGAATAACAAAGAATAACAAAGAATAACATATAATAACATAGATTTACATAGAATAACATAGAATAACATAGAATAACATAGAATAACATAGAATAACATAGAATAACATAGAGAAACATAGAATAACATAGAATAACATAGAATAACATAGAATAACATAGAATAACATAGAATGACATAGAATAACATAGAATAACATAGAATAACATAGAATAACATAGAATAACAA

>518762_4_250

AGAATAACATAGAATAACATAAAATATCACAGAATAACATAGAATATCACAGAATAACATAGAATAACATAGAATAACATAGAATAACATAGAATAACATAGAATAACATAGAATGACATAGAATAACATAGAATAACATAGAATAACATAGAATAACATAGAATAACATAGAATAACATAGAATAACATAGAATAAAATAGAATAACATAGAATAACATAGAATAACATAGAATAAAATAGAATAACAA

>530032_4_249

AGACTAACATAGAATAAAATAGAATAACATTGACTAATATCGAATAACATAGAATAACATAGAATAACATAGAATAACATAGAATAACATAGAATAACATAGAATAACATAGAATAACATAGAATAACATAGAATAACATGGAATAACATAGAATAACATAGAATAACATAGAATAACATAGAATAACATAGAATAACATAGAATAACATAGAATAACATCGAATAACATAGAATATCACAGAATAACA

>530524_4_249

AGAGAAACATAGAATAACATAGCATAACATAGAATAACATAGAATAACATAGAATAACATAGAATAACATAGAATAACATAGAATAACATAGAATAACATAGAATAACATAGAATAACATAGAATAACATAGAATAACATAGAATAAAATAGAATAACATAGAATATCACAGAATAACATAGACTAACATAGAATAACATAGAATAACATTGACTAACATAGAATAACATAGAATAACATCGAATAACA

>593927_4_250

TAGAATAACATAGAATAACATCGAATAACATCGAATAACATAGAATATCACAGAATAACATAGACTAACATGGAATAACATAGAATAACATAAAATAACATAGAATAACATGGAATAACATAGAATAACATAGAATAACATAGAATAACATAGAATAACATAGAATAACATAGAATAACATAGAATAACATAGAATAACATAGAATAACATAGAATAACATAGAATAACATAGAATAACATAGAATAACA

>595026_4_250

TAGAATAACTCAGAATAACATAGAATAACATAGAATAACATAAAATAACTTAGAATAACATGGAATAACATAGAATAACATAGAATAACATATAATAACATATAATAACATAGAATAACATAGAATAACATAGAATAACATAGAATAACATAGAATAACATAGAATAACATAGAATAACATAGAATAACATAGAATAACATTGAATAACTCAGAATAACATAGAATAACATAGAATAACATAGAATAACA

>597335_4_250

TAGAATATCACAGAATAACATAGACTCACATAGAATAAAATAGAATAACATAAAATAACATAGAATAACATAGAATAACATAGAATAACATAGAATAACATAGAATAACATAGAATAACATAGAATAACATAGAATAACATAGAATAACATAGAATAACATAGAATAACATAGAATAACATAGAATAACATAGAATATCACAGAATAACATAGACTAACATAGAATAACATAGAATAACATAAAATAACA

>654853_4_250

TTGGGATTTTACAAACATAGAATAACATAGAATAACATAGAATAACATATAATAACATAGATTTACATAGAATAACATAGAATAACATAGAATAACATAGAATAACATAGAATAACATAGAATAACATAGAATAACATAGAATAACATAGAATAACATAGAATAACATAGAATAACATAGAATAACATAGAATAACATAGAATATCACAGAATAACATAGAATATCACAGAATAACATAGAATAACGTAG

>661463_3_250

AACACTGGCGGTTTTATTTATAAACAATAGAATAACATAGAATAACATAGAATAACATAGAATAACATAGAATAACATAGAATAACATAGAATAACATAGAATAACATAGAATAACATAGAATAACATAGAATAACATAGAATAACATAGAATAACATAGAATAACATAGAATAACATAGAATAACATAGAATAACATAGAATAACATAGAATAACATAGAATAACATAGAATAACATAGCATAACATAG

>701559_3_250

ACATAAGATAACAAAGAATAACAAAGAATAACAAAGAATAACATAGAATAACATAGAATAACATAGAATAACATAAAATAACATAGAATAACATAGAATAACATAGAATAACATAGAATAACATAGAATAACATAGAATAACATAGAATAACATAGAATAACATAGAATAACATAGAATAACATAGAATAACATCGAATAACATCGAATAACATAGAATATCACAGAATAACATAGACTAACATGGAATA

>707542_3_250

ACATAGAATAACATAGAATAACATAGAATAACATAGAATAACATAGAATAACATAGAATAACATAGAATAACATAGAATAACATAGAATAACATATAACATAATAACATATAACAGAATAACATATCATAACATAGAATAACATAGAATAACATAGAATAACATAGAATAACATAGAATAACATAGAATAACATAGAATAACATAGAATAACATAGAATAACATAGAATAACATTGAATAACATAGAATA

>711602_3_250

ACATAGAATAACATAGAATAACATAGAATAACATATAATAACATAGAATAACATATAATAACATAGAATAACAAAGAATAACATTGAATAACATAGCATAACATAGAAAAGCATAGAATAACAAAGAATAACATAGAATAACATATAATAACATAGAATAACATAGAATAACATAGAATAACATATAATAACATAGAATAACATATAATAACATAGAATAGCATAGAATAACATAGAATAACATAGAATA

>717811_3_250

ACATAGAATATCACAGAATAACATAGACTAACATAGAATAACATAAAATAACAAAGAATAACAAAGAATAACAAAGAATAACATAGAATAACATAGAATAACATAAAATAACAAAGAATAACAAAGAATAACAAAGAATAACATAGAATAACATAGAATAACATAGAATAACATAGAATAACATAGAATAACATAGAATAACATAGAATAACATAGAATAACATAGAATAACATAGAATAACATAGAATA

>726601_3_250

AGAAAGAATAGAATAGAAAGAATAGAATAGAATATAATAACATAGAATAACATAGAATAACATAGAATAACATAGAATAACATAGAATAACATAGAATAACATAGAATAACATAGAATAACATAGAATAACATAGAATAACATAGAATAACATAGAATAACATAGAATAACATAGAATAACATAGAATAACATAGAATAACATAGAATAACATAGAATAACATAGAATAACATAGAATAACATAGAATAA

>740342_3_249

AGAATAACATAGAATATCACAGAATAACATAGAATATCACAGAATAACATAGAATAACATAGAATAACGTAGAATAACATAGAATAACATAGAATAAAATAGAATAACATAGAATAACATAGAATAACATAGAATAACATAGAATAACATAGAATAACATAGAATAACATAGAATAACATAGAATAACATAGAATAACATAGAATAACATAGAATATCACAGAATAACATAGAATAACATGGAATAACA

>750789_3_250

AGAATATCACAGAATAACATAGAATATCACAGAATAACATAGAATAACATAGAATAACGTAGAATAACATAGAATAACATAGAATAAAATAGAATAACATAGAATAACATAGAATAACATAGAATAACATAGAATAACATAGAATAACATAGAATAACATAGAATAACATAGAATAACATAGAATAACATAGAATATCACAGAATAACATAGAATAACATGGAATAACATAGAATAACATAGCATAACAA

>847753_3_250

TAACATAGAATAACATAGAATAACATAGAATAACATATAATAACATAGAATAACATATAATAACATAGAATAACATAGAATAACATAGAATAACATAGAATAACATTGAATAAGATAGCATAACATAGAAAAGCATAGAATAACAAAGAATAACATAGAATAACATATAATAACATAGCATAACATAGAATAACATAGAATAACATAGAATAACATAGAATAACATAGAATAACATTGAATAAGATAGCA

>866415_3_250

TAGAAGAACATAGAATGACATAGAAGAACATAGAATAACATAGAATAACATAGAATAACATATAATAACATAGATTTACATAGAATAACATAGAATAACATAGAATAACATAGAATAACATAGAGAAACATAGAATAACATAGCATAACATAGAATAACATAGAATAACATAGAATAACATAGAATAACATAGAATAACATAGAATAACATAGAATAACATAGAATAACATAGAATAACATAGAATAACA

>874694_3_250

TAGAATAACATATAATAAAGTAGAATGACATAGAATAACATTGAATAACATAGAATAACATCGAATAACATTAAATAACATAGAACAACATAGAATAACATAGAATAGCATAGAATAACAAAGAATAAAGTAGAATAACATAGAATAACATTGAATAACATTGAATAACATAGAATAACATAGAATAACATAGAATAACATAGAATAACATAGAATAACATAGAATAACATAGAATAACATAGAATAACA

>875318_3_250

TAGAATAACATTGAATAACATAGAATAACATAGAATAACATCGAATAACATAGAATAACATAGAATAACATAGAATAACATAGAATAAAATAGAATAACATAGAATAACATAGAATAACATAGAATAACATAGAATAACATAGAATAACATAGAATAACATAGAATAACATAGAATAACATAGAATAACATAGAATATCACAGAATAACATAGAATATCACAGATAAACATAGAATAACATAGAATAACG

>998603_2_249

AAAGTCTTCTTATTTGGGATTTTACAAACATAGAATAACATAGAATAACATAGAATAACATATAATAACATAGATTTACATAGAATAACATAGAATAACATAGAATAACATAGAATAACATAGAATAACATAGAATAACATAGAATAACATAGAATAACATAGAATAACATAGAATAACATAGAATAACATAGAATAACATAGAATAACATAGAATATCACAGAATAACATAGAATATCACAGAATAAC

>1104756_2_250

ACAGAATAACATAGACTAACATAGAATAATATAGAATAACATAAAATAACAAAGAATAACATAGAATAACATAGAATAACATAGAATAACATAGAATAACATAGAATAACATAGAATATCACAGAATAACATAGAATAACATAGAATAACATAGAATAACATAGAATAACATAGAATAACATAGAATAACATAGAATAACATAGAATAACATAGAATAACATAGAATAACATAGAATAACAATATCACAG

>1108785_2_250

ACATAAAATAACAAAGAATAACATAGAATAACATAGAATAACATAGAATAACATAGAATAACATAGAATAACATAGAATATCACAGAATAACATAGAATAACATAGAATAACATAGAATAACATAGAATAACATAGAATAACATAGAATAACATAGAATAACATAGAATAACATAGAATAACATAGAATAACATAGAATAACAATATCACAGAATAACATAGAATAACATAGAATAAAATAGAATAACAA

>1133580_2_250

ACATAGAATAACATAGAATAACATAGAATAACATAGAATATCACAGAATAACATAGAATATCACAGATAAACATAGAATAACATAGAATAACGTAGAATAACATAGAATAACATAGTATAACATATAATAACATAGAATAACATAGAAGAACATAGAATAACATAGAATAACATAGAATAACATTGAATAACATAGAATAACATAGAATAACATCGAATAACATAGAATAACATAGAATAACATAGAATA

>1154315_2_250

ACATATAATAACACAGCATAACATAGAATAACATAGAATAACAAGAATAACATAGAATAACATAGAATAACATAGAATAACATAGAATAACATAGAATAACATAGAATAACATAGAATAACATAGAATAACATAGAATAACATAGAATAACATAGAATAACATAGAATAATATAGAATAACATAGAATAACATAGCATAACATAGAAACAAAGAATAACATTGAATAACATAGAAAAACATAGAATAACA

>1184514_2_250

AGAATAACATAGAATAAAATAGCATAACATAGAATAACAAAGAATAACATAGCATAACATAGAATAACATAGAATAACATAGAATAACATAGAATAACATAGAATAACATAGAATAACATAGAATAACATAGAATAACATAGAATAACATAGAATAACATAGAATAACATAGAATAACATAGAATAACATAGAATAACATAGAATAACATAGAATAACATTGGATAACATAAAAAAAACATAGAATAACA

>1187726_2_249

AGAATAACATAGAATAACATAGAATAACATAGAATAACACAGAATAACATAGAATAACATAGAATAACATAGAATAACATAGAATAACATAGAATATCACAGAATAACATAGAATAACATGGAATAACATAGAATAACATAGAATAACATAGAATAACATAGAATAACATAGAATAACATAGAATATCACAGAATAACATAGAATAACATAGAATAACGTAGAATAACATAAAATAACATAGAATAAAA

>1187872_2_250

AGAATAACATAGAATAACATAGAATAACATAGAATAACATAAAATAACAAAGAATAACAACGAATAACAAAGAGTAACATAGAATAACATAGAATAACATAGAATAACATAGAATAACATAGAATAACATAGAATAACATAGAATAACATAGAATAACATAGAATAACATAGAATAACATAGAATAACATAGAATAACATAGAATAACATAGAATAACATAGAAGAACATAGAAGAACATAGAATAACAA

>1211317_2_250

AGAATAACATGGAATAGAAAGAATAGAATAGAAAGAATAGAATAGAATATAATAACATAGAATAACATAGAATAACATAGAATAACATAGAATAACATAGAATAACATAGAATAACATAGAATAACATAGAATAACATAGAATAACATAGAATAACATAGAATAACATAGAATAACATAGAATAACATAGAATAACATAGAATAACATAGAATAACATAGAATAACATAGAATAACATAGAATAACATAG

>1212767_2_250

AGAATAACGTAGAATAACATAGAATAACATAGAATAAAATAGAATAACATAGAATAACAATATCACAGAATAACATAGAATAACATAGAATAACGTAGAATAACATAGAATAACATAGAATAAAATAGAATAACATAGAATAACATAGAATAACATAGAATAACATAGAATAACATAGAATAACATAGAATAACATAGAATAACATAGAATAACATAGAATAACATAGAATAACATAGAATAACATAGAA

>1240103_2_250

AGACTCACATAGAATAAAATAGAATAACATAAAATAACATAGAATAACATAGAATAACATAGAATAACATAGAATAACATAGAATAACATAGAATAACATAGAATAACATAGAATAACATAGAATAACATAGAATAACATAGAATAACATAGAATAACATAGAATATCACAGAATAACATAGACTAACATAGAATAACATAGAATAACATAAAATAACAAAGAATAACAAAGAATAAAAAAGAATAACAA

>1265531_2_250

AGGATTGGTCCAAGTACCGATCCTTGGGGAAGAGAAGAGAAGAGAAGAGAAGAGAAGAGAAGAGAAGAGAAGAGAAGAGAAGAGAAGAGAAGAGAAGAGAAGAGAAGAGAAGAGAAGAGAAGAGAAGAGAAGAGAAGAGAAGAGAAGAGAAGAGAAGAGAAGAGAAGAGAAGAGAAGAGAAGAGAAGAGAAGAGAAGAGAAGAGAAGAGAAGAGAAGAGAAGAGAAGAGAAGAGAAGAGAAGAGAAGAGG

>1285725_2_250

ATAACAAAGAATAACAACGAATAACAAAGAGTAACATAGAATAACATAGAATAACATAGAATAACATAGAATAACATAGAATAACATAGAATAACATAGAATAACATAGAATAACATAGAATAACATAGAATAACATAGAATAACATAGAATAACATAGAATAACATAGAATAACATAGAAGAACATAGAAGAACATAGAATAACATAGAATAACATAGAATATCATATAATAACATAGATTTACATAAA

>1446553_2_78

GATCGGAAAAGCACACGTCTGAACTCCAGTCACTGACCAATCTCGTATGCCGTCTTCTGCTTGAAAAAAAAAAAATAA

>1446615_2_102

GATCGGAAGAGCACACGTCTGAACTCCAGTCACTGACCAATCTCGTATGCCGTCTTCTGCTTGAAAAAAAAAACTCTTTCCCTACACGACGCTCTTCCGATC

>1450162_2_250

TAACAAAGAATAACAAAGAACATAGAATAACGTAGAATAACATAGAATAACATAGAATAACATATAATAACATAGATTTACATAGAATAACATAGAATAACATAGAATAACATAGAATAACATAGAATAACATAGAATAACATAGAATAACATAGAATAACATAGAATAACATAGAATAACATAGAATAACATAGAATAACATAGAATAACATAGAATAACATAGAATAACATAGAATAACATAGAATAA

>1537844_2_250

TAGAATAACATAGAATAAAATAGAATAACATAGAATATCACAGAATAACATAGAATATCACAGAATAACATAGAATAACATAGAATAACGTAGAATAACATAGAATAACATAGAATAAAATAGAATAACATAGAATAACATAGAATAACATAGAATAACATAGAATAACATAGAATAACATAGAATAACATAGAATAACATAGAATATCACAGAATAACATAGAATAACATGGAATAACATAGAATAACA

>1542039_2_250

TAGAATAACATAGAATAACATAGAATAACATAGAATAACATAGAATAACATAGAATAACATAGAATAACATAGAATAACATAGAATAACATAGAATAACATAGAATAACATAGAAAAACATAGAATAACATAGAATGACATAGAATAACATAGAATAACATAGAATAACATAGAATATCACAGAATAACATAGAAGAACATAGAATAACATAGAATAACATATAATAACATCGATTTACATAGAATAACA

>1546944_2_250

TAGAATAACATAGAATAACATAGAATAACATAGAATAACATAGAATAACATCGAATAACATAGAATATCACAGAATAACATAGACTAACATAGAATAACATAAAATAACATAGAATAACATAGAATAACACAGAATAACATAGAATAACATAGAAAAACATAGAATAACATAGAATAACATAGAATAACATAGAATAACATAGAATAACATGGAATAACATAGAATAACATAGAATAACATAGAATAACA

>1571516_2_250

TAGACTAACATAAAATAACATAAAATAACATAGAATAACATAGAATAACACAGAATAACATAGAATAACATAGAATAACATAGAATAACATAGAATAACATAGAATAACATAGAATAACATAGAATAACATAGAATAACATAGAATAACATAGAATAACATAGAATAACATAGAATAACATAGAATAACATAGAATAACATAGAATATAACAGAATAACATAGAATAACATAGAATGACATAGAAGAACA

>1589432_2_79

TATCGGAAGAGCACACGTCTGAACTCCAGTCACTGACCAATCTCGTATGCCGTCTTCTGCTTGAAAAAAAAAAACAAAA

>1695043_2_250

TGAGTCTATGTTATTCTGTGATATTCTATGTTATTCGATGTTATTCTATGTTATTCTATGTTATTCTTTGTTATTCTTTGTTATTTTATGTTATTCTTTGTTATTCTATGTTATTCTATGTTATTCTGTGATATTCTATGTTATTCTATGTTATTCTATGTTATTCTATGTTATTCTATGTCATTCTATGTTATTCTATGTTATTCTATGTTATTCTATGTTATTCTATGTTATTCTATGTTATTCTATG
