# Supplementary material for: Simple and Complex Centromeric Satellites in Drosophila Sibling Species
Source: Genetics. 2018 Jan 5;208(3):977–90. doi: 10.1534/genetics.117.300620 (PMC5844345; doi:10.1534/genetics.117.300620)
Supplement: Supplementary file 7 [file 977FileS3.docx]

**File S3. Top 100 reference sequences that map input DNA from ML82-19a cells.**

>1058

AGAAGAGAATAGAAGAGAATAGAAGAGAATAGAAGAGAATAGAAGAGAATAGAAGAGAATAGAAGAGAATAGAAGAGAATAGAAGAGAATAGAAGAGAATAGAAGAGAATAGAAGAGAATAGAAGAGAATAGAAGAGAATAGAAGAGAATAGAAGAGAATAGAAGAGAATAGAAGAGAATAGAAGAGAATAGAAGAGAATAGAAGAGAATAGAAGAGAATAGAAGAGAATAGAAGAGAATAGAAGAGAAG

>15069

ACACTGCACTCTATTTATGAGCTGATTTAATGCTATTAGAGCATTTATAAGGCACTGTTTTCAGGCACTTTTTATTTAATTTATGCTCTTATGGCATATACACTGCACTCTATTTATGAGCTGATTTAATGCTATTAGAGCATTTATAAGGCACTGTTTTCAGGCACTTTTTAATTTCACAATTGCTGATGTATGGCCTCAAGCACGCCTTACCACAATTTATAATGGTACACAAAGCAACCTTTAGCT

>118340

AAACACACACGCACTTGAGAGCAAGGCAACAAATTTTCTCAAATGCACATTGATTCGGTTTTCATGGAATTTTGAAACGGAATAGAAGAGAATAGAAGAGAATAGAAGAGAATAGAAGAGAATAGAAGAGAATAGAAGAGAATAGAAGAGAATAGAAGAGAATAGAAGAGAATAGAAGAGAATAGAAGAGAATAGAAGAGAATAGAAGAGAATAGAAGAGAATAGAAGAGAATAGAAGAGAATAGAAGAG

>22245397

AAAGAGAACAAAAGAGAAGAGAACAAAAGAGAAGAGAACAAAAGAGAAGAGAAGAGAAGAGAAGAGAAGAGAAGAGAAGAGAAGAGAACAAAAGAGAAAAGAAGAGAAGAGAACAAAAGAGAAGAGAACAAAAGAGATGCACAAGTGTGCAGAAGACAGTCTTCTATCTCCACTTTATCGATGGGGCTTCGGAAAGT

>402421

AACAGAACATGTTCGAACAGAACATGTACGAAGAGAACATGTTCGAACAGAATATCTTCGAACAGAATATCTTCGAACATCGGCGGATTGTGCCTTTATTTATTTATCTTTGAAACGCACCGTGCAATCACATCCTGGAGCAACTCCACCAGCCCCAAAAAGGGGAGCAAGCCGGCCGGAATGAAGCGAATTTCAATTGTCTCAATGCCAGACAGCGATATCCAGGAAGGCAAAGCGGCGATGCTGGACG

>403100

AACAGAACATGTTCGAACAGAACATGTTCGAACAGAACATGTTCGAACAGAACATGTTCGAACAGAACATGTTTGAACAGAACATAATCGAACAGAACACGTTCGAACAGAACATAATCGAACAGAACATGTTCGAACAGAACATGTTCGAACAGAACATAATCGACCAGAACATTTTCGAACATCCATTCGCAGGCCACTTCCAGTACTGGAGAGGAAAATGTGCTCTTCGCCAAGATTTGCTTGGCCC

>624266

AACTGAACGGGGAAAGCGGGCGCAATCGACTATTTGCATGACAGAGCCAATAGAATAGAATAGAAGAGAATAGAATAGAAGAGAATAGAAGAGAATAGAATAGAATAGAAGAGAATAGAAGAGAATAGAAGAGAATAGAATAGAAGAGAAGAGAATAGAAGAGAATAGAAGAGAATAGAAGAGAATAGAAGAGAATAGAAGAGAAT

>694833

AAGACTTTAATGAATTGTGTCAAGTTGCCAAACACCTCGTCATCAATTTAGTGACGCATATGATATTGTCCCTATCATATAATTAATATAAAGAATTTTAATGAATTGTGTCAAGTTGCCAAACACCTCGTCATCAATTTAGTGACGCATATGATATTGTCCCTATCATGTTCTCCTTCGACTTTTCTGGAACCTAGTGGCTGCAATGAGATCGATGAGAACCTAACATCGCTGGACGGCAACCACTGCG

>696654

AAGAGAAGAGAACAAAAGAGAAGAGAAGAGAAGAGAAGAGAACAAAAGAGAAGAGAACAAAAGAGAAGAGAACAAAAGAGAAGAGAACAAAAGAGAAGAGAACAAAAGAGAAGAGAAGAGAAGAGAAGAGAAGAATGCCGGTTGCACTGATTTGTTTGGAATTCACTTAGGCGCCAAAGTTCTGCAATGGAAATAATTGAAAGAAACTTTTTAGTGCGATATGACTAGTAATGTAGAGGTAACTAATCAA

>696728

AAGAGAAGAGAAGAGAAGAGAAGAGAAGAGAAGAGAAGAGAAGAGAATAGAAGAGAATAGAAGAGAATAGAAGAGAATAGAAGAGAATAGAATAGAATAGAAGAGAATAGAAGAGAATAGAAGAGAATAGAAGAGAATAGAATAGAATAGAATAGAATAGAATAGAATAGAATAGAATAGAAGAGAATAGAATAGAATAGAAGAGAATAGAATAGAAGAGAATAGAAGAGAATAGAAGAGAAGAGAAGAG

>696976

AAGAGAATAGAAGAGAATAGAAGAGAATAGAAGAGAATAGAAGAGAATAGAAGAGAATAGAAGAGAATAGAAGAGAATAGAAGAGAATAGAAGAGAATAGAAGAGAATAGAAGAGAATAGAAGAGAATAGAAGAGAATAGAAGAGAATAGAAGAGAATAGAAGAGAATAGAAGAGAATAGAAGAGAATAGAAGAGAATAGAATCCGCTTGGCAATTTTCCGACTGGCATGTGGCATTCCAAGGAAGAAAG

>768969

AAGCATCTTACAGCTAAAGGTAGCAAAAATAAGTGACTCTTGTTTCCCCCTACCAAAGGTAAGGAACAGAGTATAAATATAAAAAGCAAAAAGATACAAAAGAATCTTTTATGTTTTAAAACAAGCACCTTATAGTCTATAGCTAAAGGTTGCTTTGTGTACCATTATAAATTGTGGTAAGGCGTGCTTGAGGCCATACATCAGCAATTGTGAAATTAAAAAGTGCCTGAAAACAGTGCCTTATAAATGC

>932821

AAGGGAAACCCCGAAAGGAAACGGAAATGGAAATTTTTACTTGGCAGGCGAAATTATCATGGGAACCAACGCCAACCAACTGAAAAGGAAGTTTGTTCTCCTGCAGCGAGAAAGAGAAAAAGGGAAAGAGAGAGAGGGAGAGATGCGGATGGCATTGAACAGAACACGTTCGATCAGAACATGTTCGAACAGAACATGTTCGAACAGAACAGAACAGTACAATTACGAATAGAACACGTTCGAACAGAAA

>981054

AAGGTTGCTTTGTGTACCATTATAAATTGTGGTAAGGCGTGCTTGAGGCCATACATCAGCAATTGTGAAATTAAAAAGTGCCTGAAAACAGTGCCTTATAAATGCTCTAATAGCATTAAATCAGCTCATAAATAGAGTGCAGTGTATATGCCATAAGAGCATAAATTAAATAAAAAGTGCCTGAAAACAGTGCCTTATAAATGCTCTAATAGCATTAAATCAGCTCATATCCGATCCCCAGGCCAC

>1035803

AAGTGCATCCATCGGTCGTCACACAGAAGGTACCTCCTAGCAAAAAGCTCTTCACAAGCTGGAAGAAGAGAATAGAAGAGAATAGAAGAGAATAGAAGAGAATAGAAGAGAATAGAAGAGAATAGAAGAGAATAGAAGAGAATAGAAGAGAATAGAAGAGAATAGAAGAGAATAGAAGAGAATAGAAGAGAATAGAAGAGAATAGAAGAGAATAGAAGAGAATAGAAGAGAATAGAAGAGAATAGAAGAG

>1095660

AATAGAATAGAAGAGAATAGAATAGAAGAGAATAGAATAGAAGAGAAGAGAATAGAAGAGAAGAGAAGAGAAGAGAAGAGAATAGAATAGAAGAGAATAGAAGAGAATAGAAGAGAAGAGAAGAGAAGAGAATAGAAGAGAATAGAAGAGAATAGAAGAGAATAGAAGAGAATAGAAGAGAATAGAAGAGAATAGAAGAGAATAGAAGAGAAGAGAAGAGAAGAGAAGAGAATAGAAGAGAAGAGAAGAG

>1099666

AATATAAAGACTTTAATGAATTGTGTCAAGTTGCCAAACACCTCGTCATCAATTTAGTGACGCATATGATATTGTCCCTATCATATAATTTTTGATATAAAGACTTTAAAGAATTGTATCAAGTTGCCAAACACCTCGTCATCAATTTAGTGACGCATATGATATTGTCCATATCATATAATTAATATAAAGACTTTAATGAATTGTGTCAAGTTGCCAAACACCTCGTCATCAATTTAGTGACGCATAG

>1152468

AATCGAAAAGAACATGTTCGAACAGAACATGTTCGAACAGAACATGTTCGAACAGAACATAATCGAACAGAACATGTTCGAACAGAACATAATCGAACAGAACATTTTCGAACAGAACATAATCGAACAGAACATTTTCGAACAGAACATAATCGAACAGAACATGTTCGAACAGAACATGTTCGAACAGAACATGTTCGAACAGAACATGTTCGAACAGAACATGTTCGAACAGAACATGTTTGAACAG

>1157082

AATCGAACAGAAGATGTTCGAACAAAACATGTTCAAACAAAAATGTTCGAACAGAACATGTACGAACAGAACATGTTTGAACAGAACATGTTCGAACAGAACACGTTCGAACAGAAGATGTTCGAACAGAACATGTTCGAACAGAACATGTTCGAACAGAACATAATCGAACAGAACATGTTCGAACAGGACATGGTCGAACAGATGTAACCATAGGCATCGTAACGCCTTCCAACGCCATCAAATCCGG

>1159579

AATCGACCCCGCCCCCTCCGCCGCCCTTTCCCCTGAGAGAAGAGAACAAAAGAGAAGAGAACAAAAGAGAACAAAAGAGAACAAAAGAGAAGAGAACAAAAGAGAAGAGAACAAAAGAGAAGAGAAGAGAAGAGAAGAGAAGAGAAGAGAACAAAAGAGAAGAGAAGAGAAGAGAACAAAAGAGAAGAGAACAAAAGAGATGGCGACCTCAAACCAGGGAGCTCCTCCAACTCCATCCCAACCAGCTTGG

>1302134

ACAAAACCAACAGCGACGACGTTAAATCGTGTGCCTTGTTGAGAGAATAGAAGAGAATAGAAGAGAATAGAAGAGAATAGAAGAGAATAGAATAGAATAGAAGAGAATAGAAGAGAATAGAAGAGAATAGAAGAGAATAGAAGAGAATAGAAGAGAATAGAAGAGAATAGAAGAGAATAGAAGAGAATAGAAGAGAATAGAAGAGAAGAGAAGAGAAGAGAAGAGAATAGAAGAGAATAGAAGAGAATAG

>1303832

ACAAAAGAGAAGAGAACAAAAGAGAAGAGAACAAAAGAGAAGAGAACAAAAGAGAAGAGAACAAAAGAGAAGAGAAGAGAAGAGAAGAGAAGAAAAGAGAAGAGAAGAGAAGAGAAGAGAAGAGAAGAAAAGAGAAGAGAAGAGAAGAGAAGAGAAGAGAAGAGAAGAGAAGAGAAGAGAAGAGAAGAGAACAAAAGAGAAGAGAAGAGAAGAGAAGAGAAGAGAACAAAAGAGAAGAGAAGAGAAGAG

>1535198

ACACTTTCTGCAACGGAAACTGGTCTGGAAGCTTCCTTGGAGGGCAAAGGAGATTTTTCGGCCACTGATTCTCTCCTAGATTCTTCCTTAGATTTCTCACCTTCGTCCTTAGCACTTTCTGCAACGGAAGCTGGTCTGGAAGCTTCCTTGGAGGGCAAAGGAGATTTTTCGTCCACCGATTCTCTCCTTGATTCTTCCTTAGATTTCTCACCTTCGTCCTTAGCACTTTCTGCAACGGA

>1544856

ACAGAACATGTTAAAACAGAACATGTTCGAACAGAACAAGTAATTTCGAAATGAACATGTTCGAACAGAACAAGTAATTTCGAAATGAACATGTTCGAACAAAACATGTTCGAACAGAACATAATCAAACAGAACATGTTGGAACAGAACATGTTCGAACAGAACATGTTCGAACAGAACATGTTCGAACAGAACATGTTCGAACAGAACATGTTCGAACAGAACATGTTCGAACAGAACATAATCGAA

>1551532

ACAGAACATTTTCGAACAGAACATTTTCGAACAGAACATAATCGAACAGAACATTTTCGAACAGAACATAATCGAACAGAACATGTTCGAACAGAACATGTTCGAACATGAGCCTTTTCAAGCTGTGCGGCCATTTTGCATGAGTTTTGTTTCTGTTTTTCTTGGCTGCCGTTTGTTTTTGCACGAGTGTTTATCAATAGTTGCCCCGTTTTTGTTGTGCGTTCTCGTTGTCGTTGTTGTTGATTGATT

>1555205

ACAGAATAGAACAGAATAGAACAGAATAGAACAGAATAGAACAGAATAGAACAGAATAGAACAGAATAGAACAGAATAGAACAGAATAGAACAGAATAGAACAGAATAGAACAGAATAGAACAGAATAGAACAGAATAGAACAGAATAGAACAGAATAGAACAGAATAGAACAGAATAGAAAAGCAGGGATCTCCTCCGAAGCAAAGGACACGAAAAGAAGCGAAACTAAATCAAAGCAAAA

>1735736

ACATGTTCGAACAGAACAAGTTCGAACAGAACATGTTCGAACAGAATATCTTCTAACAGAACATGTTCGAACAGAACATGTACGAAGAGAACATGTTCGAACAGAACATAATCGAACAGAACATTTTCGAACAGAACATGTTCGAACAGAAAATGTTCGAACAGAACATGTTTGAACAGAACATAATCGAACAGAACATGTTCGAACAGAACATGTTCGAACAGAACATGTTCGAACAGAACA

>1737189

ACATGTTCGAACAGAACATGTACGAAGAGAACATGTTCGAACAGAACATGTACGAAGAGAACATGTTCGAACAGAACAAGTTCGAACAGATCATGTTCGAACAGAATATCTTCTAACAGAACATGTTCAAACAGAACATGTACGAAGAGAACATGTTCGAACAGAACATGTACGAAGAGAACATGTTCGAACAGAACAAGTTCGA

>1738705

ACATGTTCGAACAGAACATGTTCGAACAGAACATGTTCGAACAGAACATAATCGAACAGAACATGTTCGAACAGAACATGTTCGAACAGAATATGTTCGAACAGAACATGTTCGAACAGAACATGTTCGAACAGAACATGTTCGAACAAAACATGTTCGAACAGAACGTGTTCGAACAGAACATGTTCGAACAGAACATGTTTGAACAGGACATGTTCGAACATCTTTCGCGACTTTGGTGTGGTGCATG

>1739438

ACATGTTCGAACAGAACATGTTCGAACAGAACATGTTCGAACAGAACATGTTCGAACAGAACATGTTCAAACAGAACATGTTCGAACCGAACATGTTCGAACAGAACATGTTCGAACAGAACAGAACATGTTCGAACAAAACATGTTCGAACAGAACAGAACATGTTCGAACAAAACATGTTCGAACAGAACATGTTCGAACAGAATATGTTTGAACAGAACATGTTTGAACAAAACATGATTGAACA

>1743238

ACATGTTCGAACAGAACGTGTGCGAACAGAACATGTTCGAACAGAACATGTTCGAACAGAACATGTTTGAACAGAACATGTTCCAACAGAACGTGTTCGAACAGAACATGTTTGAACAGAACATGTTCGAACAGAACATGTTCGAACAGAACACGTTCGAACAGAACATGTTCGAACAGAACACGTTCGAACAGAACATGTTCGAACAAAACATGTTCGAACATAACATGTTCGAACAGAACATGTACG

>2096877

ACGGCTGAACTTCATATCCGGATTCACTGAATAAAACCTTTCGACATGGACATGGAAAAGAAGAGAATAGAAGAGAATAGAAGAGAATAGAAGAGAATAGAAGAGAATAGAAGAGAATAGAAGAGAATAGAAGAGAATAGAAGAGAATAGAAGAGAATAGAAGAGAATAGAAGAGAATAGAAGAGAATAGAAGAGAATAGAAGAGAATAGAAGAGAATAGAAGAGAATAGAAGAGAATAGAAGAGAATAG

>2102239

ACGGGACCGAGTACGGGACCGAGTAAGGGACCGAGAGCGGGACCGAGTACAGGACCGAGTACGGGACCGAGTACGAGACCGTGTCGTGAAGGAGTACGAACAGAACATATTCGAACAGAACATAATCGAACAGAACAAGTTCGAAATGAACATGTTCGAACAGAACATATTCGAACAGAACATAATCGAACAGAACATGTTCGAAATGAACATGTTCGAACAGAATATCTTCGAACAGAATATCTTCGAA

>2140400

ACGTCCCGCTGCTTCTCCTGGTCAAGCCACTCACTACCCAAGAGCCCCATGCCGTTGGGAATCCCTCACATGTTCTGTTCGAACATTTTCTGTTCAAACATTTTCTGTTCGAACGTGTTCTGTTCAAACATATTCTGTTCGAACGTGTTCTGTTCGAACATGTTCTGTTCGATCATGTTCATGTTCTGGACCGAGTACAGGACCGAGTACAGGACCGAGTACAGGACCGAGTACGGCACCGAGTACGGC

>2521595

AGAACAAATTCGAACAGATTATGTTCGAACAGAACATGTTCGAACAGAACATGTTCGAACAAAACGTGTTCGAACTGAACATAATGGAACAGAACAAGTTCGAACAGAACAAGTTCGAACAGAACAAGTTCGAACAGAACATGTACGTACTCGGTCCCGTACTCGGTCCCGTACTCGGTCCAATACTCGGTCCCGTACTCGGTCCCGTACTCGGTCCCGTACTCG

>2529120

AGAACAGAACATGTTCGAACAGAACATATTCGAACAGAACATGTTCGAACAGCATATCTTCTAACAGAACAAGTTCGAACAAAACATGTTCGAACAGAACATGTTCGAACAGAACACGTTCCAACAGAACATGTTCGAACAGAACACGTTCGAACAGAACATGTTCGAACAGACCAAATTCGAAATGAACATGTTCGAACAGAACATTCATCATTTTGCCCGCAGTGTATTTGTGTGACCACCCAGACC

>2529723

AGAACAGAATAGAACAGAATAGAACAGAATAGAACAGAATAGAACAGAATAGAACAGAATAGAACAGAATAGAACAGAATAGAACAGAATAGAACAGAATAGAACAGAATAGAACAGAATAGAACAGAATAGAACAGAATAGAACAGAATAGAACAGAATAGAACAGAATAGAACAGAATAGAACAGAATAGAACAGAATAGAACAGAATAGAACAGAATAGAACAGAATAGAACAGAATAGAACAGAAG

>2529727

AGAACAGAATAGAACAGAATAGAACAGAATAGAACAGAATAGAACAGAATAGAACAGAATAGAACAGAATAGAACAGAATAGAACAGAATAGAACAGAATAGAACAGAATAGAACAGAATAGAACAGAATAGAACAGAATAGAACAGAATAGAACAGAATAGAACAGAATAGAATGGTCCGCCTGGCTCGAGGACTGAAGCCCCAGTCGGCTCGAAGCACACGCGCCAATGCTCCAACGACTCC

>2538267

AGAACATGTTCGAACAGAACATAATCGAACAGAACATGTTCGAACAGGACATGTTCGAACAGGACATGGTCGAACAGATTATGTTCGAACAGAACATGTTCGAACAGAACATGTTCGAACAGAACATTTTCGAAAAGAACATAATCGAACAGAAAATGTTCGAACAAAACATACAGATGACCCGCTTTTCTCGGAGATCGTGGCGAAGGCGGAGGGGGCCATCGAGAATGGTGTGCTGCCCGAGCGCATT

>2538575

AGAACATGTTCGAACAGAACATATTCGAACAGAACATGTTCGAACAGCATATCTTCTAACAGAACAAGTTCGAACAAAACATGTTCGAACAGAACATGTTCGAACAGAACACGTTCCAACAGAACATGTTCGAACAGAACACGTTCGAACAGAACATGTTCGAACAGACCAAATTCGAAATGAACATGTTCGAACAGAACATGTTCGAACAGAACATAATCGAACAGAACATGTTCGAACAGAACATA

>2538893

AGAACATGTTCGAACAGAACATGTACGAAGAGAACATGTTCGAACAGAATATCTTCTAACAGAACAAGTTCAAACAGAACATGTACGAAGAGAACATGTTCGAACAGATGCACACCCTGATTTCTTTAATTTTTCTAAGTTATAAGGATTGTTCTACCGAAACGATAAATATTCTTTGCGTCTAAATCAAACTGCAATAAGTGGCGGCTGGCCCAAAACACTGAGGCGAATAAGTCTTCTCATGGTGCGG

>2545585

AGAACATGTTCGAACAGAACATGTTCGAACAGAATATGTTCGAACAGAACATGTTCGAACAGAACATAATCAAACAGAAAATGTTCGAACAAAACATGTTCGAACTGAACATGTTCGAACAGAACACGTTCCAACAGAACATGTTCAAACAAAACATGTTCGAACAGAACATGTTCGAACAGAACATGTTCAAACAAAACATGTTCGAACAGAACATTAGTCTTATTCAGCCCTGGTAAAGACCCAAAGG

>2585761

AGAAGAGAACAAAAGAGAAGAGAAGAGAACAAAAGAGAAGAGAAGAGAACAAAAGAGAAGAGAAGAGAACAAAAGAGAAGAGAAGAGAACAAAAGAGAAGAGAAGAGAACAAAAGAGAAGAGAAGAGAACAAAAGAGAAGAGAAGAGAACAAAAGAGAAGAGAAGAGAACAAAAGAGAAGAGAAGAGAAGAGAAGAGAAGAGAAGAGAAGAGAAGAGAAGAGAAGAGAAGAGAAGAGAAGAGAAGAGAAG

>2585762

AGAAGAGAACAAAAGAGAAGAGAAGAGAACAAAAGAGAAGAGAAGAGAACAAAAGAGAAGAGAAGAGAACAAAAGAGAAGAGAAGAGAAGAGAAGAGAACAAAAGAGAAGAGAAGAGAACAAAAGAGAAGAGAAGAGAACAAAAGAGAAGAGAAGAGAACAAAAGAGAAGAGAAGAGAACAAAAGAGAAGAGAAGAGAAGAGAAGAGAAGAAAAGAGAAGAAAAGAAAAGAGAAGAGAAGAGAAGAGAAG

>2585854

AGAAGAGAAGAGAAGAGAAGAGAAGAGAAGAGAACAAAAGAGAAGAGAAGAGAAGAGAAGAGAAGAGAAGAGAAGAGAAGAGAAGAGAAGAGAAGAGAACAAAAGAGAAGAGAACAAAAGAGAACAAAAGAGAAGAGAACAAAAGAGAACAAAAGAGAACAAAAGAGAAGAGAACAAAAGAGAAGAGAACAAAAGAGAAGAGAAGAGAAGAGAAGAGAAGAGAACAAAAGAGAAGAGAACA

>2585877

AGAAGAGAAGAGAAGAGAAGAGAAGAGAAGAGAAGAGAAGAGAAGAGAAGAGAAGAGAAGAGAACAAAAGAGAAGAGAAGAGAAGAGAAGAGAAGAGAAGAGAAGAGAAGAGAAGAGAAGAGAAGAGAAGAGAAGAGAACAAAAGAGAAGAGAACAAAAGAGAACAAAAGAGAAGAGAACAAAAGAGAACAAAAGAGAACAAAAGAGAAGAGAACAAAAGAGAAGAGAACAAA

>2585989

AGAAGAGAAGAGAAGAGAAGAGAAGAGAAGAGAAGAGAAGAGAAGAGAAGAGAAGAGAAGAGAAGAGAAGAGAATAGAAGAGAATAGAAGAGAATAGAAGAGAATAGAAGAGAATAGAAGAGAATAGAAGAGAATAGAAGAGAATAGAAGAGAATAGAAGAGAATAGAATAGAATAGAATATGGTGCCGGGGAAGTGGTCAAGCTTCTCGACATTGGGCTTCTTGTAGGCCACGGCCACCTTGATCTC

>2585998

AGAAGAGAAGAGAAGAGAAGAGAAGAGAAGAGAAGAGAAGAGAAGAGAAGAGATGAGAAGAGATGAGAAGAGATGAGAAGAGATGAGAAGAGATGAGAAGAGATGAGAAGAGATGAGAAGAGATGAGAAGAGATGAGAAGAGAATAGAAGAGAATAGAAGAGAATAGAAGAGAATAGAAGAGAATAGAAGAGAATAGAAGAGAATAGAAGAGAATAGAAGAGAATAGAAGAGAATAGAAGAGAATAGAAG

>2586018

AGAAGAGAAGAGAAGAGAAGAGAATAGAATAGAAGAGAATAGAAGAGAATAGAAGAGAAGAGAATAGAAGAGAATAGAAGAGAATAGAAGAGAAGAGAATAGAAGAGAAGAGAATAGAAGAGAAGAGAAGAGAATAGAAGAGAATAGAAGAGAATAGAAGAGAATAGAAGAGAATAGAAGAGAATAGAAGAGAATAGAAGAGAATAGAAGAGAATAGAAGAGAATAGAATAGAATAGAATAGAATAGAA

>2586156

AGAAGAGAATAGAAGAGAATAGAAGAGAATAGAAGAGAATAAGAGAATAGAAGAGAATAGAAGAGAATAGAAGAGAATAGAAGAGAATAGAAGAGAATAGAAGAGAATAGAAGAGAATAGAAGAGAATAGAAGAGAATAGAAGAGAATAGAAGAGAATAGAAGAGAATAGAAGAGAATAGAAGAGAATAGAAGAGAATAGAAGAGAATAGAAGAGAATAGAAGAGAATAGAAGAGAATAGAAGAGAA

>2586172

AGAAGAGAATAGAAGAGAATAGAAGAGAATAGAAGAGAATAGAAGAGAATAGAAGAGAATAGAAGAGAAGAGAAGAGAATAGAATAGAATAGAAGAGAATAGAAGAGAAGAGAATAGAAGAGAATAGAAGAGAATAGAAGAGAAGAGAATAGAAGAGAATAGAAGAGAAGAGAAGAGAAGAGAATAGAATAGAAGAGAAGAGAAGAGAATAGAAGAGAATAGATCGATTTCAGTCATCAGAGGTCGCATG

>2586174

AGAAGAGAATAGAAGAGAATAGAAGAGAATAGAAGAGAATAGAAGAGAATAGAAGAGAATAGAAGAGAATAGAAGAGAAGAGAAGAGAAGAGAAGAGAAGAGAATAGAATAGAAGAGAATAGAAGAGAAGAGAATAGAAGAGAATAGAAGAGAATAGAAGAGAAGAGAATAGAAGAGAAGAGAATAGAAGAGAAGAGAAGAGAATAGAAGAGAATAGAAGAGAATAGAAGAGAAGAGAAGAGAATAGAAG

>2586199

AGAAGAGAATAGAAGAGAATAGAAGAGAATAGAAGAGAATAGAAGAGAATAGAAGAGAATAGAAGAGAATAGAAGAGAATAGAAGAGAATAGAAGAGAATAGAAGAGAATAGAAGAGAATAGAAGAGAATAGAAGAGAATAGAAGAGAATAGAAGAGAATAGAAGAGAATAGAAGAGAATAGAAGAGAATAGAAGAGAATAGAAGAGAATAGAAGAGAATACGCCCCACAAAATAGCAGCCACGCCCCAC

>2586211

AGAAGAGAATAGAAGAGAATAGAAGAGAATAGAAGAGAATAGAAGAGAATAGAAGAGAATAGAAGAGAATAGAAGAGAATAGAAGAGAATAGAAGAGAATAGAAGAGAATAGAAGAGAATAGAAGAGAATAGAAGAGAATAGAAGAGAATAGAAGAGAATAGAAGAGAATAGAAGAGAATTGTGCAAATTGCCAGTTTACATTCAAGCCACACACACTCACATAACCATCCGACAAGTTGCCGTGCC

>2586275

AGAAGAGAATAGAAGAGAATAGAAGAGAATAGAAGAGAATAGAATAGAATAGAAGAGAATAGAAGAGAATAGAATAGAATAGAATAGAATAGAATAGAATAGAATAGAAGAGAAGAGAAGAGAAGAGAAGAGAATAGAAGAGAATAGAAGAGATGGGCCATTAGTCACCCGGTCCAAATCTATGTGTCTGCGGTGAAATGGCTTTCGATTGACCTGGTCCCGGTATCAAACGTCTGGATACATCATTAG

>2586333

AGAAGAGAATAGATGAGAATAGAAGAGAATAGAAGAGAATAGAAGAGAATAGAAGAGAATAGAATAGAATAGAAGAGAATAGAATAGAATAGAAGAGAATAGAAGAGAAGAGAAGAGAAAAGAAGAGAATAGAAGAGAATAGAAGAGAATAGAAGAGACAGCGGCGTCAGTCCCTGCTCTGCCAGGCCGCTGAGTGTCTCTCGATCTTCGCGCAAATCGATTTTGGTGCCTGGAATACGCCCCAAGATGG

>2618771

AGAATAGAAGAGAAGAGAAGAGAAGAGAATAGAATAGAAGAGAAGAGAATAGAAGAGAATAGAAGAGAATAGAATAGAATAGAATAGAATAGAAGAGAATAGAAGAGAATAGAATAGAATAGAATAGAATAGAATAGAATAGAAAGGAGTGTTTGGCCGCAATAGTAGCTCTCAAAAACTTCAGAGCATACGTGGAAGGACTCCCTTTTAAAATAATAACCGACCATGCTTCGCTCAAGTGGCTAATGTC

>2619247

AGAATAGAATAGAATAGAATAGAATAGAATAGAATAGAATAGAATAGAATAGAATAGAAGAGAATAGAAGAGAAGAGAATAGAATAGAAGAGAAGAGAAGAGAAGAGAATAGAAGAGAATAGAAGAGAAGAGAAGAGAAGAGAAGAGAAGAGAAGAGAAGAGAAGAGAATAGAAGAGAAGAGAAGAGAAGAGAAGAGAATAGAAGAGAATAGAATAGAATAGAACAGAATAGAAGAGAATAGAATAGAAA

>2635974

AGAATTCTGGATGGCAACGGGCATTCAATTCTTACAGTGTGAGTATCTCACATAGAAGAGAATAGAAGAGAATAGAAGAGAATAGAAGAGAATAGAAGAGAATAGAAGAGAATAGAAGAGAATAGAAGAGAATAGAAGAGAATAGAAGAGAATAGAAGAGAATAGAAGAGAATAGAAGAGAATAGAAGAGAATAGAAGAGAATAGAAGAGAATAGAAGAGAATAGAAGAGAATAGAAGAGAATAGA

>2651080

AGACAAGAATAGACAAGAATAGAATAGACAAGAATAGACAAGAATAGAATAGACAAGAATAGACAAGAATAGAATAGACAAGAATAGACAAGAATAGAATAGACAAGAATAGACAAGAATAGAATAGACAAGAATAGACAAGAATAGAATAGACAAGAATAGACAAGAATAGAATAGACAAGAATAGACAAGAATAGAATAGACAAGAAT

>2715512

AGACCTCCAATGAGCCGCAACGCGAGAACTACGATGATGTATGTTCGAACAGAACATGTTCGAACAGAAGATGTTCGAACAGAACATGTTCGAACAGAAGATGTTCCGAACAGAACATGTTCGAACAGAACATGTTCGAACAGAACATAATCGAACAGAACATGTTCGAACAGACCATGTTCGAACACAACATGTTCGAACAGAACATGTTCGAACAGAACATCCGATCATTTCCGTTTCCGATTTGGC

>2790226

AGAGAACAAAAGAGAAGAGAACAAAAGAGAAGAGAAGAGAAGAGAAGAGAAGAGAAGAGAAGAGAAGAGAAGAGAACAAAAGAGAAGAGAAGAGAAGAGAACAAAAGAGAAGAGAACAAAAGAGAACAAAAGAGAAGAGAACAAAAGAGAAGAGAAGAGAAGAGAAGAGAAGAGAAGAGAAGAGAAGAGAAGAGAAGAGAAGAGAAGAGAAGAGAAGAGAAGAGAAGAGAAGAGAAGAGAAGAGAAG

>2790243

AGAGAACAAAAGAGAAGAGAAGAGAAGAGAACAAAAGAGAAGAGAAGAGAACAAAAGAGAAGAGAAGAGAAGAGAACAAAAGAGAAGAGAAGAGAACAAAAGAGAAGAGAAGAGAAGAGAACAAAAGAGAAGAGAAGAGAACAAAAGAGAAGAGAAGAGAAGAGAACAAAAGAGAAGAGAAGAGAACAAAAGAGAAGAGAAGAGAACA

>2794247

AGAGAAGAGAACAAAAGAGAAGAGAAGAGAAGAGAAGAGAACAAAAGAGAAGAGAAGAGAAGAGAAGAGAACAAAAGAGAAGAGAAGAGAAGAGAAGAGAACAAAAGAGAAGAGAACAAAAGAGAAGAGAACAAAAGAGAAGAGAACAAAAGAGAAGAGAACAAAAGAGAAGAGAAGAGAAGAGAAGAAAAGAGAAGAGAAGAGAAGAGAAGAGAAGAAAAGAAAAGAGAAGAGAAGAGAAGAGAAG

>2794280

AGAGAAGAGAAGAGAACAAAAGAGAAGAGAAGAGAACAAAAGAGAAGAGAAGAGAACAAAAGAGAAGAGAAGAGAACAAAAGAGAAGAGAAGAGAACAAAAGAGAAGAGAAGAGAAGAGAAGAGAAGAGAAGAGAAGAGAAGAGAAGAGAAGAGAAGAGAAGAGAAGAGAAGAGAAGAGAAGAGAAGAGAAGAGAACAAAAGAGAAGAGAAGAGAAGAGAAGAGAAGAGAAGAGAAGAGAAGAGAACAA

>2794323

AGAGAAGAGAAGAGAAGAGAACAAAAGAGAAGAGAAGAGAAGAGAAGAGAACAAAAGAGAACAAAAGAGAAGAGAACAAAAGAGAACAAAAGAGAACAAAAGAGAACAAAAGAGAAGAGAACAAAAGAGAACAAAAGAGAAGAGAACAAAAGAGAAGAGAACAAAAGAGAACAAAAGACTCCGTTTCTCATTTCGAACAACGTATGTTCATTTTTTTCCAGCAGTTTCTTGATATCTTGAACTTTTTGAT

>2794341

AGAGAAGAGAAGAGAAGAGAAGAGAACAAAAGAGAAGAGAAGAGAAGAGAAGAGAACAAAAGAGAACAAAAGAGAAGAGAACAAAAGAGAACAAAAGAGAACAAAAGAGAAGAGAACAAAAGAGAACAAAAGAGAAGAGAACAAAAGAGAAGAGAACACGAATATAAACAATATTATATCTTTATATCCTTGATAACTACTCGTCGAACTCGTTTTGTTTGTAAAAATGTTAAAAAAACCAAACTTAAA

>2794422

AGAGAAGAGAAGAGAAGAGAAGAGAAGAGAAGAGAAGAGAAGAGAAGAGAAGAGAACAAAAGAGAAGAGAAGAGAAGAGAACAAAAGAGAAGAGAAGAGAAGAGAAGAGAAGAGAAGAGAAGAGAAGAGAAGAGAAGAGAAGAGAAGAGAAGAGAAGAGAAGAGAAGAGAAGAGAAGAGAAGAGAAGAGAAGAGAACAAAAGAGTGAGAAAGTCAGATACAGCTACAGTTGCAGATACGTATACACAGG

>2794424

AGAGAAGAGAAGAGAAGAGAAGAGAAGAGAAGAGAAGAGAAGAGAAGAGAAGAGAACAAAAGAGAAGAGAAGAGAAGAGAAGAGAACAAAAGAGAAGAGAAGAGAAGAGAAGAGAACAAAAGAGAAGAGAAGAGAAGAGAAGAGAACAAAAGAGAAGAGAAGAGAAGAGAAGAGAACAAAAGAGAAGAGAACAAAAGAGAAGAGAACAAAAGAGAAGAGA

>2794435

AGAGAAGAGAAGAGAAGAGAAGAGAAGAGAAGAGAAGAGAAGAGAAGAGAAGAGAAGAGAACAAAAGAGAAGAGAAGAGAAGAGAAGAGAAGAGAAGAGAAGAGAAGAGAAGAGAAGAGAAGAGAAGAGAAGAGAAGAGAAGAGAACAAAAGAGAAGAGAACAAAAGAGAACAATGCAGGCCAGTAGCGATTCTCCCGCCTTGGTGGCAAGTTGCAATTCCAACTGCGGCTGCAGTCGCACCAACTACGG

>2797742

AGAGAATAGAAGAGAAGAGAAGAGAAGAGAAGAGAAGAGAATAGAAGAGAATAGAAGAGAATAGAAGAGAATAGAAGAGAATAGAATAGAATAGAAGAGAAGAGAAGAGAATAGAAGAGAATAGAAGAGAATAGAAGAGAATAGAAGAGAATAGAAGAGAATAGAAGAGAAGAGAAGAGAAGAGAATAGAAGAGAATAGAAGAGAAGAGAAGAGAAGAGAATAGAAGAGAATAGAAGAGAATAGAAGAG

>2797762

AGAGAATAGAAGAGAATAGAAGAGAATAGAAGAGAATAGAAGAGAATAGAAAAGAATAGAAGAGAATAGAAGAGAATAGAAGAGAATAGAAGAGAATAGAAGAGAATAGAAGAGAATAGAATAGAAGATAGATGAAATAAAAAATAATAAGTAAGCCTAATTAATAGCAATTTTGTTAAAGAATGATTTCCTAAGCTTTTACTACGAATACTTCAAAGATGAAAATTGTTTTGCTCAGTGTAAGGAAAC

>2797829

AGAGAATAGAAGAGAATAGAAGAGAATAGAAGAGAATAGAAGAGAATAGAAGAGAATAGAAGAGAATAGNAGAGAATAGAAGAGAATAGAAGAGAATAGAAGAGAATAGAAGAGAATAGAAGGGAATAGAAGAGAATAGAAGAGAATAGAAGGGAATAGAAGAGAATAGAAGAGAATATTTCCGAGGGAGGCTACTATGCCAGGTCGCACGACT

>2797848

AGAGAATAGAAGAGAATAGAAGAGAATAGAATAGAATAGAATAGAAGAGAAGAGAAGAGAAGAGAAGAGAAGAGAATAGAAGAGAATAGAAGAGAATAGAAGAGAATAGAAGAGAAGAGAATAGAAGAGAATAGAAGAGAATAGAAGAGAAGAGAAGAGAAGAGAAGAGAATAGAAGAGAATAGAATAGAATAGAATAGAATAGAATAGAATAGAATAGAATAGAATAGAATAGAATAGAATAGAAT

>2799054

AGAGAATGGAAGAGAATGGAAGAGAATGGAAGAGAATGGAAGAGAATGGAAGAGAATGGAAGAGAATGGAAGAGAATGGAAGAGAATAGAAGAGAATAGAAGAGAATAGAAGAGAATGGAAGAGAATGGAAGAGAATAGAAGAGAATGGAAGAGAATGGAAGAGAATGGAAGAGATGCTCCGGGGGCAGCGACTGCCGTCGGATGCCCAGCAGGCGGTTGCGCACATCGACGAGACTGGCGGAGGGCGGG

>2853477

AGAGCATTTATAAGGCACTGTTTTCAGGCACTTTTTATTTAATTTATGCTCTTATGGCATATACACTGCACTCTATTTATGAGCTGATTTAATGCTATTAGAGCATTTATAAGGCACTGTTTTCAGGCACTTTTTAATTTCACAATTGCTGATGTATGGCCTCAAGCACGCCTTACCACAATTTATAATGGTACACAAAGCAACCTTTAGCTATAGACTATAAGGTGCTTGTTTTAAAACATAAA

>2908351

AGAGGCAACCACCGCCTCTTCGCAAGAGTCCCACGCCACCGGAACCTAAAGTGCCTCAGTCGGAGGACTTCGCACATGACGATGTTCGAACAGAACATGTTTGAACAGAATATCTTCGAACAGAATATCTTCGAACAGAACATTTTCGAACAGAACAAGTTTGAAATGAACATGTTCGAACAGAACAAGTTCGAACAGAACAAGTTCGAACAGAACAAGTTCGATCAGCTGTTAGCAGGCTAAAACAAAG

>3079325

AGATTCTGTAGAGGAAGATTCATTAGCAGGTTCTGTCGATGATTCAGTAGTCAAAGGACCTTCAGTGCTAGAGCTACTTTCTTCTGTAGTACTATCTTGTGAAGATTCTGTAGAGGAAGATTCATTAGCAGGTTCTGTAGATGATTCAGTAGACAAAAGACCTTCAGTGCTTGAGCTACTTTCTTGTGTCGTACTATCTTGTGAAGATTCTGTAGAGGAAGACTCATTAGATGGTTCTGTAGATGATTCA

>3675004

AGGACATGGTCGAACAGATTATGTTCGAACAGAACATGTTCGAACAGAACATGTTCGAACAGAACATTTTCGAACAGAACATAATCGAACAGAACATAATCAAACAGAACATGTTCGAACAGAACATGTTCGAACAGAACATAATCGAACAGAACATGTTCGAACAGAACATAATCGAACAGAACATGTTCGAAAAGAACACGTTCCAACAGAACATGTTCGAACAGAACATAA

>3851324

AGGCATTCGAGTGCACGGTCTGCGGCAAGGGACTCGCCCGCAAGGACAAGCTGACCATCCACATGCGCATCCACACCGGCGAGAAGCCCTAAGAACAGAATAGAACAGAATAGAACAGAATAGAACAGAATAGAACAGAATAGAACAGAATAGAACAGAATAGAACAGAATAGAACAGAATAGAACAGAATAGAACAGAATAGAACAGAATAGAACAAAATAAAACAAAATAGAAAAGAATAGAAC

>4179964

AGGTGACCAACGATAAGTTGGTTTTCAGTAGACACCCGCTAAGGCCCGAAACACAAATCACAACCAGACCAGAGTTGGTGAGCATCGAAAATGATCTGTTCGATTATGTTCTGTTCGAAAATATTCTGTTCGAAAATGTTCTGTTCGATTATGTTCTGTTCGAACATGTTCTGTTCGAACATGTTCTGTTGGAACGTGTTCTGTTCGAACATGTTCTGTTCGAACATGTTCTGTTCGAACATGTTCTAG

>4246391

AGGTTTAGCTGGCAAGTGGCTACCCATTCTGGGCAAACACCCCGCAAATTACTTTGCAATTTGTTTGTTTTGTTCGAACATTTTCTGTTCGATTATGTTCTTTTCGAAAATGTTCTGTTCGAACATGTTCTGTTCGAACATGTTCTGTTCGAACATAATCTGTTCGACCATGTCCTGTTCGAACATGTCCTGTTCGAACATGTTCTGTTCGATTATGTTCTGTTCGAACATGTTCTGTTCGATTGGATC

>4266408

AGTAACGGCTGGTTCGCTCATCTGTTTCGATAACGAAAGAATGAATCCCTCTGCCCGCGAGTCCGACTTGGGGTCCTCGCAGCGGAGGAAGGAGGCACAGCTGGGCGACTTCGACATAGAAGAGAATAGAAGAGAATAGAAGAGAATAGAAGAGAATAGAAGAGAATAGAAGAGAATAGAAGAGAAGAGAAGAGAAGAGAAGAGAATAGAAGAGAAGAGAAGAGAAGAGAAGAGAAGAGAAGAGAAGAG

>4645306

AGTGGCTGCGACTCAGGGAATCTTGGTGGCGCTGGCGGCAGCAATTCCACTAAGACCCAACTGGAGTCCCAAGAGTTGGATGTACAACCGGCAGAGCTGGAAATGAGCGCTAGTGGCTGCGACTCAGGGAATCTTGGTGGCGCTGGCGGCAGCAATCCCACTAAGACCCAACTGGAGTCCCAAGAGGTGGATGTACAACAGGCAGAGC

>4773414

AGTTCGAAATGAACATGTTCGAACAGAACATATTCGAACAGAACATAATCGAACAGAACATGTTCGAACAGAACATGTTGGAACAGAACATGTTCGAACAGAACATGTTCGAACAGAACATGTTCGAACAGAACATGATCGAACAGAACATAATCGAACAGAACATGTTCGAACAGAACATGTACGAAGAGAACATGTTCGAACAGAATATCTTCTAACAGAACAAGTTCAAACAGAACATGTACGAA

>5178379*

ATCATCTACAGAACCTGCTAATGAATCTTCCTCTACAGAATCTTCACAAGATAGTACGACACAAGAAAGTAGCTCTAGCACTGAAGGTCCTTTGACTACTGAATCATCTACAGACCCTTCTAATGAGTCGTCCTCTACAGAATCTTCACAAGATAGTACGACTCAAGAAAGTAGCTCAAGCACTGAAGGTCCTTTGACTACTGAATCATCTACAGAACCTTCTAATGAGTCGTCCTCTACAGAATCTTC

>5211611*

ATCCATCCAGACATCCGTGGCGAAGCATAGCTTGCCATTGAACGGAAGTGAGCGGAAGGTCAGCTGTAGCGTCAGGATCTCTGCCCACGACACCTGGAGCAGCTTCATCTGGTCGTTAAGTGGCAGATCTATAAAGCCAGGTATCTGCTTGGCCCATGTGTACCATTATAAATTGTGGTAAGGCGTGCTTGAGGCCATACATCAGCAATTGTGAAATTAAAAAGTGCCTGAAAACAGTGCCTTATAAATG

>5278712

ATCGAACAGAAAATGTTCGAACAAAACATGTTCAAACAAAACATGTTCGAAAAGAACACGTTCCAACAGAACATGTTCGAACAGAACATGTTCGAACAGAACATGTTCGAACAGAACATGTTCGAACAGAACATGTTCGAACATCGGAGTTTGACTGGCTACTTTTCAGAGCATCTGCGTTTTGACCTAATCTTACCCCATTACCCCCACCTAGTGTGTAATTTCTAGTGTATGGATTTAGGCAAT

>5507111*

ATGAGCTGATTTAATGCTATTAGAGCATTTATAAGGCACTGTTTTCAGGCACTTTTTATTTAATTTATGCTCTTATGGCATATACACTGCACTCTATTTATGAGCTGATTTAATGCTATTAGAGCATTTATAAGGCACTGTTTTCAGGCACTTTTTAATTTCACAATTGCTGATGTATGGCCTCAAGCACGCCTTACCACAATTTATAATGGTACACAAAGCAACCTTTAGCTATAGACTATAAGGTGCG

>5942132

ATGTACGAAGAGAACATGTTCGAACAGAACAAGTTCGAACAGGACATAATCGAACAGAACATGTTCGAACAGAATATCTTCTAACAGAACATGTTCGAACAGAACATGTACGAAGAGAACATGTTCGAACAGAATATCTTCGAACAGAATATCTTCGAACAGAACATAATCGAACAGAACATGTTCGAACAGAATATCTTCTAACAGAACATGTTCGAACAGAACATGTACGAAGAGAACATGTTCGAA

>6063834

ATGTTCTGTTCGAACATAATCTGTTCGACCATGTCCTGTTCGAACATGTCCTGTTCGAACATGTTCTGTTCGATTATGTTCTGTTCGAACATGTTCTGTTCGATTATGTTCTGTTCGAACATGTTCTGTTCTATTATGTTCTGTTCGAACATCTTCTGTTCGAACGTGTTCTGTTCGAACATGTTCTGTTCGAAATTGTTCTGTTCGAACATGTTCTGTTCGACAATGTTCTGTTCGAACATGTTCTGT

>6381105

CGAACAGAAAATGTTTGAACAAAACATGTTCAAACAAAACATGTTCGAACAGAACATGTTCGAACAAAACATGTTCGAACAGAACATGTTCGAACAGAACATTTCCCCCTCTAGTAGGAAAACACACGCCAACCAGCCAGCCAGAACCCAAAACGAGAATAATAAAGCTGAAAATGGAATGGAAGGATGGGTGGGATGGGTGGAAAAGTGTGCTAAAGAGTCCATAAGAAAAACTCTCGTAATAGACAAA

>6872086

TAGAATAGAAGAGAAGAGAAGAGAAGAGAATAGAAGAGAAGAGAAGAGAAGAGAAGAGAATAGAAGAGAATAGAAGAGAAGAGAAGAGAATAGAAGAGAATAGAATAGAATAGAAGAGAATAGAAGAGAATAGAATAGAATAGAAGAGAATAGAAGAGAATAGAATAGAATAGAAGAGAATAGAATAGAATAGAATAGAATAGAATAGAATAGAATAG

>7199238

TCGAACATGTTCTGTTCCAACATGTTCTGTTCGAACATGTTCTGTTCGATTATGTTCTGTTCGAATATGTTCTGTTCGAACATGTTCTCTTCGTACATGTTCTGTTCGAACATGTTCTCTTCGTACATGTTCTGTTCGAACATGTTCTGTTCGATTATGTTCTGTTCGATCATGTTCTGTTCGAACATGTTCTGTTCGAACATGTTCATTTCGAACTTGTTCTGTTCGAACATGTTCTGTTCG

>7318914

TCTGTTCGAACATGTTCTGTTCGAACATGTTCTGTTCGAACATGTTCTGTTCGATTATGTTCTGTTCGAACATGTTCTGTTCGAACATGTTCTCTTCGTAGATGTTCTGTTCGAACATGTTCTGTTAGAAGATATTCTGTTCGAACATGTTCTGTTCGATTATGTTCTGTTCGAACATGTTCTGTTAGAAGATATTCTGTTCGAACATGTTCTGTTCGAACTTGTTCTGTTCGAAGATATGT

>7319428

TCTGTTCGAACATGTTCTGTTCGAACATGTTCTGTTCTATTATGTTCTTTTCGAACATATTCTGTTCGATTATGTTCTGTTCGAACATGTTCTGTTCGAACTTGTTCTGTTCGAACATGTTCTCTTCGTACATGTTCTGTTCGAACATGTTCTGTTAGAAGATATTCTGTTCGAACATGTTCTGTTCGAAGATATTCTGTTCGAACATGTTCTGTTCGAACATGTTCTGGTCGAACATGTTCTGTTCGAA

>7321893

TCTGTTCGATTATGTTCTGTTCGAAAATGTTCTGTTCGATTATGTTCTGTTCGAACATGTTCTGTTCGATTATGTTCTGTTCGAACATGTTCTGTTGGAACGTGTTCTTTTCGAACATGTTCTGTTCGAACATGTTCTGTTCGATTATGTTCTGTTCGAACATGTTTTGTTCGAAAATGTTCTGTTCGAAAATGTTCTGTTCGATTATGTTCTGTTCGATTATGTTCTGTTCAAACATGTTCTGTTCAAA

>7526863

TGATTTTGTTCTGTTCGAACATGTTCTGTTCGATTATGTTCTGTTCGAACATGTTTTGTTTGAACATGTTCTGTTCGAACATGTTCTGTTCTTAAATGTTCTGTTCGAACATGTTCTGTTCTAACATGTTCTGTTCGAAGATATTCTGTTCCAACATGTTCTGTTCGAACATGTTCTGTTCGAACATGTTCTGTTCGATTATGTTCTGTTCGAACATGTTCTGTTCGATTATGTTCTGTTCGAACATG

>8121648

TGTGTGTGTGCCTACTTAATCCAATTGGAGGTGGAGCCGTGTTTCTCAAATGGAAATTAGCAGGATTTTAAAATGGATTTGATAACTACTGACAGGCTCAAAGAACACTTAACGAAGCATTGATTGCATAAAAATGTTTAGTTGCTTAAATGTTGTAATAAAAAGGGCTTATCAGCAATTGTGAAATTAAAAAGTGCCTGAAAACAGTGCCTTATAAATGCTCTAATAGCATTAAATCAGCTCATAAATA

>8156755

TGTTCGAACATGTTCTGTTCGAACATGTTCTGTTCGAACATGTTCTGTTCGAACATGTTCTGTTCGAACATGTTCTGTTCGAACATGTTCTGTTCGAACATGTTCTGTTCGAACATGTGCTGTTCGAACATATTCTGTTCGAATATGTTCTGTTCGATTATGCTCTGTTCGATTATGTTCTGTTCGAACATGTTCTGTTCGATTATGTTCTGTTCGAACATGTTCTCTTCGTAGATGTTCTGTTC
