# Supplementary material for: Simple and Complex Centromeric Satellites in Drosophila Sibling Species
Source: Genetics. 2018 Jan 5;208(3):977–90. doi: 10.1534/genetics.117.300620 (PMC5844345; doi:10.1534/genetics.117.300620)
Supplement: Supplementary file 8 [file 977FileS4.docx]

**File S4. Top 100 reference sequences that map IP reads from ML82-19a cells.**

>1058

AGAAGAGAATAGAAGAGAATAGAAGAGAATAGAAGAGAATAGAAGAGAATAGAAGAGAATAGAAGAGAATAGAAGAGAATAGAAGAGAATAGAAGAGAATAGAAGAGAATAGAAGAGAATAGAAGAGAATAGAAGAGAATAGAAGAGAATAGAAGAGAATAGAAGAGAATAGAAGAGAATAGAAGAGAATAGAAGAGAATAGAAGAGAATAGAAGAGAATAGAAGAGAATAGAAGAGAATAGAAGAGAAG

>12966

AATCGTCGACGCATAAATCTCAAGACCTGCCCGGCGGCTGCAAAGAGAAGAGAAGAGAAGAGAAGAGAAGAGAAGAGAAGAGAAGAGAAGAGAAGAGAAGAGAAGAGAAGAGCAGAGAAGAGCAGAGAAGAGCAGAGAAGAGAATAGAAGAGAAGAGAAGAGAATAGAAGAGAATAGAAGAGAATAGAAGAGAATAGAAGAGAATAGAAGAGAATAGAAGAGAATAGAAGAGAATAGAAGAGAATAGAAG

>66273

AAAACAAATTTAAAACACAATCGTTTAGCATGTACCAATTTGCTTTGAGTGATGCACTGCAACGCTGATAATTATGAAAAACGTAACAAAATATAATGCTTATGGTAAGCAATTGGTTGTTGCTTTATGAAATTGAAGATTTGTTTAGTGCTAGAACTAGAATTCTCTAGGCTTGATGTTTACAATGCTGGAACAAATTTAAATAACAAAACGTTAAGGT

>118340

AAACACACACGCACTTGAGAGCAAGGCAACAAATTTTCTCAAATGCACATTGATTCGGTTTTCATGGAATTTTGAAACGGAATAGAAGAGAATAGAAGAGAATAGAAGAGAATAGAAGAGAATAGAAGAGAATAGAAGAGAATAGAAGAGAATAGAAGAGAATAGAAGAGAATAGAAGAGAATAGAAGAGAATAGAAGAGAATAGAAGAGAATAGAAGAGAATAGAAGAGAATAGAAGAGAATAGAAGAG

>151837

AAACCATTAAAACTTTCACAGTAGCATATTTTGTTATGTATATCTATTAGCAACTGTTTTAAATTTCATGTTGCTAGTTAATTACCGCTAATTAATATTAAATATTTTTTGATTTTGATCGTCGATTATCAAAACAACATAAGTACTTACTTATTTTAAAAATATAACGAATTATTTAGATACAAACCTTTTATTCCTTGGTCCTTGATTAACGACATCATTTGTGCTAATTTTACATCCGCGAAGCTC

>1519781

AAACCATTACAAGTTTAGTGTTATTTGTAAACACAATAAACGATAATTAATATTAATATTTTCTGAGTCTGATTGCCGATTATCAAAACAACATAAGTACTTACTTATTTTAAAAATATAACGATCACCGAAAAAAGAGCAGGTTAACAACAATACAAAGCAACGAAATCTGAGAATCTGAAATCTTTTCCCCACAGCTTTCTCTGCTCAAGGAAAAATATCGAGGTGCCATTGAATGGAAAAGGAACGG

>205311250

AAACTTATGCTTATTGTACATAAAACTAAACTTGTAATGGTTTTGTTTTAATTTCTTTTACCCTGGGAGCCAATAAGATAGATGACCTGCGATGTAGTTTCAGGATCTGCGCGGATGAAAAATTAACACAAAGGATGTCTTAAATTAAGGACCAAAGTATAAGAGGTTCGTATCTAAATAATTCGTTATAATTTTAAAATAAGTAAGTACTTATGTTGTTTTGATAATCGGCAATCAAACTAAAAAAAAA

>311304

AAATCCCTTGTGAGCCGAGTGCCTAAATAAATATTGGCTAAGTCCCAACACAAACAATTCAGAGAGAAGAGAATAGAAGAGAATAGAAGAGAATAGAAGAGAATAGAAGAGAATAGAAGAGAATAGAAGAGAATAGAAGAGAATAGAAGAGAATAGAAGAGAATAGAAGAGAATAGAAGAGAATAGAAGAGAATAGAAGAGAATAGAAGAGAATAGAAGAGAATAGAAGAGAATAGAAGAGAATAGAAGG

>526886 rev

AACGAATTATTTAGATACGAACCTCTTAATCTTTGGTCCTTGATTTACGACATCCATTGTTCTAATTTTACTTCCGCGCAGCTCCTGAAACTACATCGCAGGTCATCTAGCTTATTGGCTCACAGAGGGTACATGAAATTAAAACAAAACCATTACAATTTTACGTTTTTCATAATTATCAGCGTTGCAGTGCACCACTCAAAGCAAATTGGTACATGCTAAACGATTGTGTTTTAAATTTTTTTTAGT

>529887

AACGACATCATTTGTGCTAATTTTACATCCAAGCAGCTCCTGAAACTGTATCGCAGGTCATATAGCTTATTGGCTCCCAGAGGGTACATGTAATTAAAACAAAACTATTACTAGTTTAGTGTTATTTGTAAACACAATAAACGCTAATTAATATTAAATATTTTTTGAGTTTGATTGCCGATTATCAAAACAATAAGTACTTACTTATTTTAAAAATAACGATAGCCCGAATAAATTGAAAGTCTCTAT

>539283

AACGATAATATTAAATATTTTTGGAATTTGATTGTCGATTATCAAAACAACATAAGTACTTACTTATTTTAAAAATATATCGAATTATTTAGATACAAACCTCTTTTTCTATGGTCCTTGAATTACGACATCCTTTGTTCTGATTTTGCATCAGCGCAGCTCCTGAAACTACATCGCAGGTCATCTAGCTTATTGGCTCCCAGAGGGTACATGTAATTAAAACAAAACCATTACAAGTTTAGTGTTATA

>607087

AACTATTTAAATGCAGATTGGTTTAGAGATAGAACATACCATTCCCTAGGTTTTAGTTTCACAATACTAAAACAAATTTAAAACACAATCGTTTAGCATGCACCAATTTGCTTTAAGTGATTAACTGCAACGCTGCTAATTATCCACCAATTTGCTTTAAGTAATGCACTGCAAAGATGATAATTATGAAAAACATAACAAAATTTAATGCTTATAGTAAGCAATTGGTTTTTGCTTTATGCAATTGAAG

>696722

AAGAGAAGAGAAGAGAAGAGAAGAGAAGAGAAGAGAAGAGAAGAGAAGAGAAGAGAAGAGAAGAGAAGAGAAGAGAAGAGAAGAGAAGAGCAGAGAAGAGAAGAGAAGAGAAGAGAAGAGCAGAGAAGAGCAGAGAAGAGCAGAGAAGAGCAGATAGAACGGAAGCGACTTCGTCACTCGCTGGCTCGCCTTGCAGGTAAAAATGGA

>696976

AAGAGAATAGAAGAGAATAGAAGAGAATAGAAGAGAATAGAAGAGAATAGAAGAGAATAGAAGAGAATAGAAGAGAATAGAAGAGAATAGAAGAGAATAGAAGAGAATAGAAGAGAATAGAAGAGAATAGAAGAGAATAGAAGAGAATAGAAGAGAATAGAAGAGAATAGAAGAGAATAGAAGAGAATAGAAGAGAATAGAATCCGCTTGGCAATTTTCCGACTGGCATGTGGCATTCCAAGGAAGAAAG

>988140

AAGTAAGTACTTATGTTGTTTTGATAATCGACATCAAATACAAAAAATATTTAATATTATCATTATATTTTTAAAATAAGTAAGTACTTATGTTGTTTTGATAATCGGCAATCAGACTCAAAAAATATTCTAAACTTGTAATGGTTTTGTTTTAATTACATGTACCCTCTGCGAGCCAATAAGCTAGATGACCTCCGATGTAGTTTCAGGAGCTGCGCTGATGCAAAATCAGAACAAAGGATGTCGTAA

>1035803

AAGTGCATCCATCGGTCGTCACACAGAAGGTACCTCCTAGCAAAAAGCTCTTCACAAGCTGGAAGAAGAGAATAGAAGAGAATAGAAGAGAATAGAAGAGAATAGAAGAGAATAGAAGAGAATAGAAGAGAATAGAAGAGAATAGAAGAGAATAGAAGAGAATAGAAGAGAATAGAAGAGAATAGAAGAGAATAGAAGAGAATAGAAGAGAATAGAAGAGAATAGAAGAGAATAGAAGAGAATAGAAGAG

>1095660

AATAGAATAGAAGAGAATAGAATAGAAGAGAATAGAATAGAAGAGAAGAGAATAGAAGAGAAGAGAAGAGAAGAGAAGAGAATAGAATAGAAGAGAATAGAAGAGAATAGAAGAGAAGAGAAGAGAAGAGAATAGAAGAGAATAGAAGAGAATAGAAGAGAATAGAAGAGAATAGAAGAGAATAGAAGAGAATAGAAGAGAATAGAAGAGAAGAGAAGAGAAGAGAAGAGAATAGAAGAGAAGAGAAGAG

>1108768

AATCAAGGACCAAGGTATAAGAGTTTCGTTTTTAAATAATTCTATATATTATAAAAATAAGTAAGTACTTATGTTGTTTTGATAATCGACAGTCAAATTCAAAAAATATTTAATATTATCGTTATATTTTTAAAATAAGTAAGTACTTATGTTGTTTTGATAATCGGCAATCAGACTCAAAAAATATTTAATATTAATTAGCGTTTATTGTGTTTACAAATAACACTAAACTAGTAATAGTTTTTTTTA

>1265444

AATGTTTTTGTTTTAATTACACGTACCTTTTTGGAGCCAATAAGCTAGATGACCTGCGATGTAGTTTCAGGAGCTGCGCGGATGTAAAATTAGAACAAGGCTGTCGTAAATCAAGGACCAAAGAATAAGAGGTTCGTAACTAAATAATTCGTTATAATTTTAAAATAAGTAAGTACTTATGTTGTTTTGATAATCGGCAATCAAACTAAAAAAATTTAATATTAATTAGCAGTTATTAACTACCACATGA

>1297993

ACAAAAAATTTACTTCATAAAAATATTGACAATATCGAATTTTTAAAATAAAATACCAATTCGTTGAAAATATATGTCCCAGGAATGGCATTCATACATTGATGACGTGGTCCATTTGACATTAAATCAGTTAAAGTGTTGGTCACGTGATCGTTAATGCTAATTAAAACTTATGCTTATTGTACATAAAACTAAACTTGTAATGGTTTTGTTTTGCACACGACTAAATATGCCAATTTAAACAAAAA

>1302134

ACAAAACCAACAGCGACGACGTTAAATCGTGTGCCTTGTTGAGAGAATAGAAGAGAATAGAAGAGAATAGAAGAGAATAGAAGAGAATAGAATAGAATAGAAGAGAATAGAAGAGAATAGAAGAGAATAGAAGAGAATAGAAGAGAATAGAAGAGAATAGAAGAGAATAGAAGAGAATAGAAGAGAATAGAAGAGAATAGAAGAGAAGAGAAGAGAAGAGAAGAGAATAGAAGAGAATAGAAGAGAATAG

>1302316

ACAAAACCATTACAAGTTTAGTGTTATTTGTAAACACAATAAACGCTAATTAATATTAAATATTTGTTGAGTCTGATTGCCGATTATCAAAACAACATAAGTACTTACTATTTATTATAACGATAATATTAAATTTTTTTTGAATTTGATTGTCGATTATCAAAACAACATAAGTACTTACTTATTTTAATAATATATAGAATTATTTAAAAACGAACCTCTTATTCCTTGGTCCTTGATTAACTACAT

>1303832

ACAAAAGAGAAGAGAACAAAAGAGAAGAGAACAAAAGAGAAGAGAACAAAAGAGAAGAGAACAAAAGAGAAGAGAAGAGAAGAGAAGAGAAGAAAAGAGAAGAGAAGAGAAGAGAAGAGAAGAGAAGAAAAGAGAAGAGAAGAGAAGAGAAGAGAAGAGAAGAGAAGAGAAGAGAAGAGAAGAGAAGAGAACAAAAGAGAAGAGAAGAGAAGAGAAGAGAAGAGAACAAAAGAGAAGAGAAGAGAAGAG

>1828661

ACCATAAGCATTCTATTTTGCATCACTCAAAGCAAATTGGTGGATAATTAGCAGCGTTGCAGTTAATCACTTAAAGCAAATTGGTGCATGCTAAACGATTGTGTTTTAAACTTGATTTAGTATTGTGGAACTCACACCTAGAGAATGGTATGTTGTAGCTCTAAACCAATCTGCATTAAAACAACGCAATAGCTTAAACTTTTAATGTGTTCCATAACCAGATATTCATTTTATTTATCAATAATAACA

>1836793

ACCATTACAAGTTTAGTGTTATTTGTAAACACAATAAACGCTAATTAATATTAAATATTTTTTGAGTCTGATTGCCGATTATCAAAACAACATAAGTACTTACTTATTTTAAAATAGAAAGCAAGTGCCGCCAAAAACGCGAAAACAGATCCACAAATCGGACCCTGAACCAGCAAACAAAAGTTGGAGGCGGATCAGTTTAAGAAGCTGCTGGCCACATGAGCTTGAAACATGTGTGGCTCCTAAGCGG

>1920360

ACCTACGATGTAAGTTTAGGAGCTATGCGAATGTACAATCAGCGAATAGGATGTCATAAATCAAGGACCATAGAATAAGAAGTTTGTATCTAAATAATTCGATATATTTTTAAAATAAGTTCTTATGTTGTTTTGATAATCGACAATCAAAATCAAAAAATATTTAATATTAATTAGCGTTAATTAACTAGCAACATGAGGTTAGCAATCCACCATGGCACTTTGGCTTTGTGGATCACTCTTTTCGTTT

>1923314

ACCTATTGTCCATGCGCTGACCAATGAGAGTGACCCTGGCTATCGACCGGCATTTTGTGGACGAAGGTTCCACGATAACAGCGGTAAACGATAATATTAAATATTTTTTGAATTTGATTGTCGATTATCAAAACAACATAAGTACTTACTTATTTTTAAAATATAACGAATTATTTAAATACGAACTTCTTATTCTTTGGTCCTTGATTTACGAAATCATTTGTGCTAATTTTACATCCGAGCATCTCCT

> rev

ACGCTTTTCATAATTATCAGCTTTGCAGTGCATCACTCAAAGCAAATTGGTGGATAATTAGCAGCGTTGCAGTTAATCACTCAAAGCAAATTGTTGCATGCTAAACGATTGTGTTTTAAAGTAGTTTTAGTATTGTGAAACTCATGAGGGACTTTCGCGCTATCCCTGTAATCCTGCGACTCAGTCTGCTCAACAGTTTCTTATTCATGGAGTGCATTTTATGCGAATTTTATGCAAATTAATGGTCCTT

>2066977

ACGGAAATTCGGCTGCTCAGCTGGCAAATGATGATGTTCGGGGTGGCCAAAAAGTCAAGTCAGCAGCACAAATGTGTGTGTGTGAATTGTGACCCAATATCGTTATATTTTTAAAATAAGTAAGTACTTATGTTGTTTTGATAATCGACAATCAAATTCCAAAAATATTTAATATTATCGTTATATATCAAAATCAAAAAATATTTAATATTAATTAGCAGTAAATAAGTAGCATTATGAAATTTAAAA

>2096877

ACGGCTGAACTTCATATCCGGATTCACTGAATAAAACCTTTCGACATGGACATGGAAAAGAAGAGAATAGAAGAGAATAGAAGAGAATAGAAGAGAATAGAAGAGAATAGAAGAGAATAGAAGAGAATAGAAGAGAATAGAAGAGAATAGAAGAGAATAGAAGAGAATAGAAGAGAATAGAAGAGAATAGAAGAGAATAGAAGAGAATAGAAGAGAATAGAAGAGAATAGAAGAGAATAGAAGAGAATAG

>2121588

ACGGTTACAGCGGCAGCATCTGGAGCACGACCTTTGAATAGAATTGAATAGAATTGAATAGAATTGAATAGAATTGAATAGAATTGAATAGACTTGAATAGAATTGAATAGAATTGAATAGAATTGAATAGAATTGAATAGAATTGAATAGAATTGAATCGAATTGAATAGAATTGAATAGAATTGAATAGAATTGAATAGAATTGAATAGAATTGAATAGAATTGAAGACAGGAAGAGCACACGTCTGG

>2244836

ACTCCGCCAGCAAGTCGTACTGATCATCGTCCGCACACCACCACACCATCATTATATTTTTAAAATAAGTAAGTACTTATGTTGTTTTGATAATCGGCAATCAGACTCAAAAAATATTTAATATTAATTAGCGTTTATTGTGTTTACAAATAACACTAAACTTGTAATGGTTTTGTTTTAATTACATGTACCCTCTGCGAGCCAACGAAGCCCAAGCCAAACTTAACTGCGAGAATTAATTCCCTTGTCT

>2400817

ACTTATGTTGTTTTGATAATCGACATCAAATACAAAAAATATTTAATATTATCATTATATTTTTAAAATAAGTAAGTACTTATGTTGTTTTGATAATCGGCAATCAGACTCAAAAAATATTTAATATTAATTAGCGTTTATTGTGTTTACAAATAACACTAAACTTGTAATGGTTTTGTTTTAATTACATGTACCCTCTGCGAGCCAATAAGCTAGATGAGCTCCGATGTAGTTTCAGGAGCTGCG

>2447119

ACTTGTAATGGTTTTGTTTTAATTTCATGTATCCTCTGGGAGCCAATAAGCTAGATGACCTGTGATGTAGATTCAGGATCTGCGCTGATGTAAAATCAGAACAAAGGATGTCGTAAATCAAGGACCAAAGAAAAGGAGGTTCGTATCTAAATAATTCGTTATATTTCTAAAATAAGTTCTTATGTTGTTTTGATAATCGACAATCAAAATCAAAAAATATTTAATATTAATTAGCGTTAATTAACTA

>2452220

ACTTTAAAACACAATCGTTTAGCATGCAACAATTTGCTTTGAGTGATTAACTGCAACGCTGCTAACTATCCACCAATTTGCTTCGTGTGATGCACTGCAACGCTGATAATTATGAAAAACGTAACAAAATATAATGCTTATAGTAAGCAATTGGTTGTTGCTTTATGCAATTTTTTAGAGCTAGAACATTTGGGGCCGTTATGCATGCGCGATGCGTGACTGCCACAATGCCCATTTTCCAGCTTTCCGC

>251637015 rev

AGAAATGGTCCATACCTTAACGTATTAAAGCAAATTGGTGGATAATTAGCAGCGTTGCAGTTAATCACTTAAAGCAAATTGGTGCATGCTAAACGATTGTGTTTTAAATTTGTTT

>2576612

AGAACTTATTTTAAAAATATATCGAATTATTTAAATACAAACGTCTTTTCTATGGTCCTTGAATTACGACATCCTTTGTTCTGATTTTGCATCAGCGCAGCTCCAGAAACTACATCGGAGGTCATCTAGCTTATTGGCTCGCAGAGGGTACATGTAATTAAAACAAAACCATTACAAGTTTAGAATATTTTTTGAGTCTGATTGCCGATTATCAAAACAACATAAGTACTTACTTATTTTAATACT

>2585762

AGAAGAGAACAAAAGAGAAGAGAAGAGAACAAAAGAGAAGAGAAGAGAACAAAAGAGAAGAGAAGAGAACAAAAGAGAAGAGAAGAGAAGAGAAGAGAACAAAAGAGAAGAGAAGAGAACAAAAGAGAAGAGAAGAGAACAAAAGAGAAGAGAAGAGAACAAAAGAGAAGAGAAGAGAACAAAAGAGAAGAGAAGAGAAGAGAAGAGAAGAAAAGAGAAGAAAAGAAAAGAGAAGAGAAGAGAAGAGAAG

>2585998

AGAAGAGAAGAGAAGAGAAGAGAAGAGAAGAGAAGAGAAGAGAAGAGAAGAGATGAGAAGAGATGAGAAGAGATGAGAAGAGATGAGAAGAGATGAGAAGAGATGAGAAGAGATGAGAAGAGATGAGAAGAGATGAGAAGAGAATAGAAGAGAATAGAAGAGAATAGAAGAGAATAGAAGAGAATAGAAGAGAATAGAAGAGAATAGAAGAGAATAGAAGAGAATAGAAGAGAATAGAAGAGAATAGAAG

>2586018

AGAAGAGAAGAGAAGAGAAGAGAATAGAATAGAAGAGAATAGAAGAGAATAGAAGAGAAGAGAATAGAAGAGAATAGAAGAGAATAGAAGAGAAGAGAATAGAAGAGAAGAGAATAGAAGAGAAGAGAAGAGAATAGAAGAGAATAGAAGAGAATAGAAGAGAATAGAAGAGAATAGAAGAGAATAGAAGAGAATAGAAGAGAATAGAAGAGAATAGAAGAGAATAGAATAGAATAGAATAGAATAGAA

>2586126

AGAAGAGAATAGAAATGAATAGAAGAGAATAGAAGAGAATAGAAGAGAATAGAAGAGAATAGAAGAGAATAGAAGAGAATAGAAGAGAATAGAAGAGAATAGAAGAGAATAGAAGAGAATAGAAGAGAATAGAAGAGAATAGAAGAGAATAGAAGAGAATAGAATAGAATAGAATAGAATAGAAGAGAATAGAATAGATGATCTTAGCTCATACAGATTTGTAGGCTCTGGGTATTCCTTAATGGCCTT

>2586156

AGAAGAGAATAGAAGAGAATAGAAGAGAATAGAAGAGAATAAGAGAATAGAAGAGAATAGAAGAGAATAGAAGAGAATAGAAGAGAATAGAAGAGAATAGAAGAGAATAGAAGAGAATAGAAGAGAATAGAAGAGAATAGAAGAGAATAGAAGAGAATAGAAGAGAATAGAAGAGAATAGAAGAGAATAGAAGAGAATAGAAGAGAATAGAAGAGAATAGAAGAGAATAGAAGAGAATAGAAGAGAA

>2586172

AGAAGAGAATAGAAGAGAATAGAAGAGAATAGAAGAGAATAGAAGAGAATAGAAGAGAATAGAAGAGAAGAGAAGAGAATAGAATAGAATAGAAGAGAATAGAAGAGAAGAGAATAGAAGAGAATAGAAGAGAATAGAAGAGAAGAGAATAGAAGAGAATAGAAGAGAAGAGAAGAGAAGAGAATAGAATAGAAGAGAAGAGAAGAGAATAGAAGAGAATAGATCGATTTCAGTCATCAGAGGTCGCATG

>2586174

AGAAGAGAATAGAAGAGAATAGAAGAGAATAGAAGAGAATAGAAGAGAATAGAAGAGAATAGAAGAGAATAGAAGAGAAGAGAAGAGAAGAGAAGAGAAGAGAATAGAATAGAAGAGAATAGAAGAGAAGAGAATAGAAGAGAATAGAAGAGAATAGAAGAGAAGAGAATAGAAGAGAAGAGAATAGAAGAGAAGAGAAGAGAATAGAAGAGAATAGAAGAGAATAGAAGAGAAGAGAAGAGAATAGAAG

>2586199

AGAAGAGAATAGAAGAGAATAGAAGAGAATAGAAGAGAATAGAAGAGAATAGAAGAGAATAGAAGAGAATAGAAGAGAATAGAAGAGAATAGAAGAGAATAGAAGAGAATAGAAGAGAATAGAAGAGAATAGAAGAGAATAGAAGAGAATAGAAGAGAATAGAAGAGAATAGAAGAGAATAGAAGAGAATAGAAGAGAATAGAAGAGAATAGAAGAGAATACGCCCCACAAAATAGCAGCCACGCCCCAC

>2586216

AGAAGAGAATAGAAGAGAATAGAAGAGAATAGAAGAGAATAGAAGAGAATAGAAGAGAATAGAAGAGAATAGAAGAGAATAGAAGAGAATAGAAGAGAATAGAAGAGAATAGAAGAGAATAGAAGAGAATAGAAGAGAATAGAAGAGAATAGAAGGGAATAGAAGAGAATAGAAGAGAATAGAAGAGAATAGAAGAGATTGGTATAACACAAACAGGGAGAAGGAGCTGTGAAGAGGGGCGAAAGAAAAA

>2586325

AGAAGAGAATAGAATAGAATAGAATAGAATAGAAGAGAATAGAATAGAATAGAATAGAATAGAAGAGAAGAGAATAGAAGAGAATAGAAGAGAATAGAAGAGAATAGAAGAGAATAGAAGAGAATAGAAGAGAATAGAAGAGAATAGAAGAGAATAGAAGAGAATAGAAGAGAATAGAAGAGAATAGAAGAGAATAGAAGAGAATAGAAGAGAATATAGATTCCAGGTGTTCTCATAATC

>2618958

AGAATAGAATAGAATAGAAGAGAAGAGAAGAGAATAGAAGAGAATAGAAGAGAATAGAAGAGAATAGAAGAGAATAGAAGAGAATAGAAGAGAATAGAAGAGAATAGAAGAGAATAGAAGAGAATAGAAGAGAAGGAGATCCTAAAATCTATTGATAACTACGAGAGCCAAGAGTCGTCGGCATCAGAGGAGGAGGATGATCTAAAGAACAAGCTTAGTGTTCATGTACCGAATCAGATGGACATACCA

>2619041

AGAATAGAATAGAATAGAATAGAAGAGAATAGAAGAGAATAGAAGAGAATAGAAGAGAATAGAAGAGAATAGAAGAGAATAGAAGAGAATAGAAGAGAATAGAAGAGAATAGAAGAGAATAGAAGAGAATAGAAGAGAAGAGAAGAGATACAAAACTCGCTATATACGAAGAAGAAGCATTTTATGTACTAACTATAGATGAAAGCAACACTTAACGAAACAACAACAGCAACAAGAAAAAAAAAAGAAA

>2620969

AGAATAGAATAGAATAGAATTGAATTGAATAGAATTGAATAGAATTGAATAGAATTGAATAGAATTGAATAGAATTGAATAGAATTGAATAGAATTGAATAGAATTGAATAGAATTGAATAGAATTGAATAGAATTGAATAGAATTGAATAGAATTGAATAGATTGCGTGGGCTTCATTTGGATGAGTGCAGGCAGGACCTGGCTGTTCTCCTTCCGGCTGCCTCATCCCCTGCCTCCGCTCATGAGCTT

>2635974

AGAATTCTGGATGGCAACGGGCATTCAATTCTTACAGTGTGAGTATCTCACATAGAAGAGAATAGAAGAGAATAGAAGAGAATAGAAGAGAATAGAAGAGAATAGAAGAGAATAGAAGAGAATAGAAGAGAATAGAAGAGAATAGAAGAGAATAGAAGAGAATAGAAGAGAATAGAAGAGAATAGAAGAGAATAGAAGAGAATAGAAGAGAATAGAAGAGAATAGAAGAGAATAGAAGAGAATAGA

>2708392

AGACCGAAAAATCTCTCAATTTGGCCTACAAAATGCTCATTTTTGTGCAAAATTTTTGGATAGAAGAGAATAGAAGAGAATAGAAGAGAATAGAAGAGAATAGAAGAGAATAGAAGAGAATAGAAATGAATAGAAGAGAATAGAAGAGAATAGAAGAGAATAGAAGAGAATAGAAGAGAATAGAAGAGAATAGAAGAGAATAGAAGAGAATAGAAGAGATGTGGCCGTGGCTGCTGCCCCTATCCCAGTG

>2785757

AGAGAAAAGGTAGCAAAAAAAAAAGTAGATATAAGAGAGAAAAATATATTTCGCCTGCGAGGCAGAAATTCCGATGCCATCAAGGACCATAGAAAAAGAGGTTTGTATCTAAATAATTCGATATATTTTTAAAATAAGTAAGTACTTATGTTGTTTTGATAATCGACAATCAAATTCCAAAAATATTTAATATTATCGTTATATATATATTGTGTTTACAAATAACACTAAACATGTAATGGTTTTGTTG

>2790226

AGAGAACAAAAGAGAAGAGAACAAAAGAGAAGAGAAGAGAAGAGAAGAGAAGAGAAGAGAAGAGAAGAGAAGAGAACAAAAGAGAAGAGAAGAGAAGAGAACAAAAGAGAAGAGAACAAAAGAGAACAAAAGAGAAGAGAACAAAAGAGAAGAGAAGAGAAGAGAAGAGAAGAGAAGAGAAGAGAAGAGAAGAGAAGAGAAGAGAAGAGAAGAGAAGAGAAGAGAAGAGAAGAGAAGAGAAGAGAAG

>2794247

AGAGAAGAGAACAAAAGAGAAGAGAAGAGAAGAGAAGAGAACAAAAGAGAAGAGAAGAGAAGAGAAGAGAACAAAAGAGAAGAGAAGAGAAGAGAAGAGAACAAAAGAGAAGAGAACAAAAGAGAAGAGAACAAAAGAGAAGAGAACAAAAGAGAAGAGAACAAAAGAGAAGAGAAGAGAAGAGAAGAAAAGAGAAGAGAAGAGAAGAGAAGAGAAGAAAAGAAAAGAGAAGAGAAGAGAAGAGAAG

>2794255

AGAGAAGAGAACAAAAGAGAAGAGAAGAGAAGAGAAGAGAAGAGAAGAGAAGAGAAGAGAAGAAAAGAGAAGAGAAGAGAAGAGAAGAGAAGAGAAGAGAAGAGAAGAGAAGAGAAGAGAAGAGAAGAGAACAAAAGAGAAGAGAAGAGAAGAGAAGAGAAGAGAAGAGAACAAAAGAGAAGAGAAGAGAAGAGAAGAGAAGAGAAGAGAAGAGAAGAGAACATCACGGGCGCAAAGGTGATGAAATG

>2794368

AGAGAAGAGAAGAGAAGAGAAGAGAAGAGAAGAGAACAAAAGAGAAGAGAAGAGAAGAGAACAAAAGAGAAGAGAAGAGAAGAGAAGAGAAGAGAAGAGAAGAGAAGAGAAGAGAAGAGAAGAGAAGAGAAGAGAAGAGAAGACAACAAAAGAGAAGAGAAGAGAACAAAAGAGAAGAGAAGAGAACAAAAGAGAAGAGAAGAGAACAAAAGAGAAGAGAGGTAGTTTTATGGGAAGAAGGAGGAGGAGG

>2797760

AGAGAATAGAAGAGAATAGAAGAGAATAGAAGAGAATAGAAGAGAAGAGAATAGAAGAGAATAGAAGAGAATAGAAGAGAATAGAAGAGAATAGAAGAGAATAGAAGAGAATAGAAGAGAATAGAAGAGAATAGAAGAGAATAGAAGAGAATAGAAGAGAATAGAAGAGAATAGAAGAGAATAGTGCCTGGGAACATCCGTTACAAATGCTTGGCTACTGTATAGGAAACAATTGAACTTAGTAAAC

>2797798

AGAGAATAGAAGAGAATAGAAGAGAATAGAAGAGAATAGAAGAGAATAGAAGAGAATAGAAGAGAATAGAAGAGAATAGAAGAGAATAGAAGAGAATAGAAGAGAATAGAAGAGAATAGAAGAGAATAGAAGAGAATAGAAGAGAATAGAAGAGAATAGAAGAGAATAGAAGAGAATAGAAGAGAATAGAATTATGTTCTGTTCGAACATATTCTGTTCGAACTTGTTCTGATCGAACATGTTCTCTT

>2797848

AGAGAATAGAAGAGAATAGAAGAGAATAGAATAGAATAGAATAGAAGAGAAGAGAAGAGAAGAGAAGAGAAGAGAATAGAAGAGAATAGAAGAGAATAGAAGAGAATAGAAGAGAAGAGAATAGAAGAGAATAGAAGAGAATAGAAGAGAAGAGAAGAGAAGAGAAGAGAATAGAAGAGAATAGAATAGAATAGAATAGAATAGAATAGAATAGAATAGAATAGAATAGAATAGAATAGAATAGAAT

>2799054

AGAGAATGGAAGAGAATGGAAGAGAATGGAAGAGAATGGAAGAGAATGGAAGAGAATGGAAGAGAATGGAAGAGAATGGAAGAGAATAGAAGAGAATAGAAGAGAATAGAAGAGAATGGAAGAGAATGGAAGAGAATAGAAGAGAATGGAAGAGAATGGAAGAGAATGGAAGAGATGCTCCGGGGGCAGCGACTGCCGTCGGATGCCCAGCAGGCGGTTGCGCACATCGACGAGACTGGCGGAGGGCGGG

>3225449

AGCATGTACCAATTTGCATTGAGTAATTAACTGCAACGCTGCTAATTTTCCACCAATTTGCTTTGAGTGATGCACTGCAAGCCTGATAATTATGAAGAAAATAACAAAATAGAATCCTTATAATAAGCAATTGGTTGTTGCTTTATGCAATTTTTTATTGCGCCCGATTCTTCTCGAAGCTGACGGCCATACTTCCACACTGACAACCACCTTGAGCAGCAAGATCTTGGCCAGCTTTCACTGGGTCGCC

>3225452

AGCATGTACCAATTTGCTTTGAGTGATGCACTGCAACGCTGATAATTATGAAAAACGTAACAAAATATAATGCTTATGGTAAGCAATTGGTTGTTGCTTTATGCAATTTTTTAGAGCTAGAACATTTGTATTCTCTAGGCTTGATATTTACAATGATGGAACAAATTTAAATAACAAAACGTTAAGGTATGGACCATTTTATTATAATTGATAACTAAAACGAATATCTGATTATGGAAAACATTAAAAG

>3231237 rev

AGCATTCTATTTTGTTGTGTTTTTCATAATTATCAGCGTTGCAGCGCATCACTCAAAGCAAATTGGTGGATAGTTAGCAGCGTTGCAGTTAATCACTCAAAGCAAATTGTTGCATGCTAAACGATTGTGTTTTAAAGTAGTTTTAGTATTGTGAAAAACGCCGACAGAATCTGGCTGGATGATTAAAATATAATTCATATGCCAGTCCAAAGTTGTGTAACATTTCCGGTGTTGTAAAAATCTCCGCTCA

>3580903

AGCTTATTGGCTCCCAGAGGGTACATGTAATTAAAACAAAACCATTACAAGTTTAGTGTTATTTTTAAACACAATAAACGCTAATTAATATTAAATATTTTTTGAGTCTGATTGCCGATTATCAAAACAACATAAGTACTTACTATTTATTATAACGATAATATTAAATATTTTTTGAATTTGATTGTCGATTATCAAAACAACATAAGTACTTACTTATTTTAAAAATATATAGAATTATTTAAAAA

>4078188

AGGGGTAGGGGGCAGGGGGTTGAGTGCTGGGTATTCAATTCTATTCAATTCTATTCAATTCTATTCAATTCTATTCAATTCTATTCAATTCTATTCAATTCTATTCAATTCTATTCAATTCTATTCAATTCTATTCAATTCTATTCAATTCTATTCAATTCTATTCTATTCAATTCTATTCAATTCTATTCTATTCAATTCTATTCAATTCATTTTAATTCTATTCTATTCTATT

>4085078

AGGGGTTGTTAGTATTGGTTGTAAGGAGTACAAAATGGTACTCCTTTTTGCTCTCTGACCATTTTTAGTCAGTTATAGCCAAAAAGCCAATATTCAATTCTATTCAATTCTATTCAATTCTATTCAATTCTATTCAATTCTATTCAATTCTATTCAATTCTATTCAATTCTATTCAATTCTATTCTATTCTATTCAATTCTATTCAATTCTATTCTATTCTATTCTATTCTATTCTATTCTATTCTATT

>4270451

AGTAAGTACTTATGTTGTTTTGATAATCGACATCAAATACAAGAAATATTTAGTATTATCGTTATATTTTTAAAATAAGTAAGTACTTATGTTGTTTTGATAATCGACAATCAAATTCCAAAAATATTTAATATTATCGTTATATATCAAAATCAAAAAATATTTAATAATAATTAGCAGTAAATAAGTAGCATTATGAAATTTAAAACAATCACTAATAGATATACATGACTTAATAT

>4301688

AGTACTTATGTTGTTTTGATAATCGGCAATCAGACTCAAAAAATATTTAATATTAATTAGCGTTTATTGTGTTTACAAATAACACTAAACTTGTAATGGTTTTGTTTTAATTACATGTACACTCTGGGAGCCAATAAGCTAGATGGCCTGCGATGTAGTTTCAGTAGCTGCTCGGATGTATGCGGCTTCTTTTTTTCGTACATTTATTTTTGTGTGTTTTACTGTTGTTGTTGTCGCGAGCGTTGACAGG

>4714083

AGTGTTGGTCACGTGATCGTTAATGCTAATTAAAACTTATGCTTATTGTACATAAAACTAAACTTGTAATGGTTTTGTTTTAATTTCTTGTACCCTGGGAGCCAATAAGATAGATGACCTGCGATGTAGTTTCAGGATCTGCGCGGATGAAAAATTAACACAAAGGATGTCTTAAATTAAGGACCAAAGTATAAGAGGTTCGTTGCATGGCAACTGCACATTACCGTTTCGTACTTTTTCATGCATTCA

>4980959 rev

ATACCATCTCTGGTACGTAGATTTCCTATGAATTGTAATTTTATTTAAAAAATTCGATATTGTCAATATTTTTATGATCGGAGGTCATCTAGCTTATTGGCTCCCAGAGGGTACATGTAATTAAAACAAAACCATTACAAGTTTAGTGTTATTTGTAAACACAATAAACGCTAATTAATATTAAATATTTTTTGAGTCTGATTGCCGATTATCAAAACAACATAAGTACTTACTATTTATTATAACGATA

>498155276

ATACCATTCTCTAGGTGTGAGTTCCACAATACTAAATCAAGTTTAAAACACAATCGTTTAGCATGCACCAATTTGCTTTAAGTGATTAACTGCAACGCTGCTAATTATCCACCAATTTGCTTTGAGTGATGCAAAATAGAATGCTTATGGTAAGCAATTGGTTGTTGCTTTATGCT

>5035950

ATAGAATAGAATAGAATAGAATAGAATAGAAGAGAATAGAAGAGAATAGAAGAGAATAGAAGAGAATAGAAGAGAATAGGAGAGAATAGAAGAGAATAGAAGAGAATAGAAGAGAATAGAAGAGAATAGAAGAGAATAGAAGAGAATAGAAGAGAATAGAAGAGTGACCTGCGATGTAGCTTCAGGAGCTGCTTGGATGT

>5036739

ATAGAATTGAATAGAATTGAATAGAATTGAATAGAATTGAATAGAATAGAATAGAATAGAATAGAATAGAATAGAATAGAATAGAATAGAATAGAATAGAATAGAATAGAATAGAATAGAATAGAATAGAATAGAATAGAATAGAATTGAATAGATTTGAATAGAATAGAATTGAATAGAATTGAATAGAATTGAATAGAATTGAATAGAATTGAATAGAATTGAATAGAATTGAATAGAATTGAATAG

>5125404

ATCAAGGACCAAGGAATAAGAGCTTCGTTTTTAAATAATTCTATATATTATTAAAATAAGTAAGTACTTATGTTGTTTTGATAATCGACAATCAAATTCAAAAAATATTTAATATTATCGTTATAATAAATAGTAAGTACTTATGTTGTTTTGATATTTGGCAATCAGACTCATAGTCAACCTCCATTGGTTCCGACCTTTGATGTTGTGGATCTTGACTTTGGAACCGTTGTCTTGAGAAATTAGAGTG

>5127241

ATCAAGTTTAAAACACAATCGTTTAGCATGCACCAATTTGCTTTAAGTGATTAACTGCAACGCTGCTAATTATCCACCAATTTGCTTTGAGTGATGCAAAATAGAATGCTTATGGTAAGCAATTGGTTGTTGCTTTATGCTATTTTTTAGAGCTAGAACATTTGTATTCTCTAGGCTTGATGTTTACAATGCTGGAACAATGCAAAAGCAACTCAAACAAAAGTGTACCCATGAAAATATCGAATGGGT

>5287660

ATCGAATTTTTAAAATAAAATACCGATTCGTTGAAAATATATGTCCCAGGGATGGCATTCATACATTGATGACGTGGTCCATTTGACATTAAATCAGTTATAGTGTTGGTCACGTGATCGTTAATGCTAATTAAAACTTATGCTTATTGTACATAAAACTAAACTTGTAATGGTTTTGTTTTAATTTCTTGTACCCTGGGAGCCAATAAGATAGATGACCTGCGATGTAGTTTCAGGATCTGCGCGGATG

>5288992

ATCGACATCAAATACAAAAAATATTTAATATTATCATTATATTTTTAAAATAAGTAAGTACTTATGTTGTTTTGATAATCGGCAATCAGACTCAAAAAATATTTAATATTAATTAGCGTTATGTGCTTCAAGAGAAATGTTTTTTTCTAGGACTGGTTTGTTGGCATTTAATTTCCCTTGAAAAAATAATTTATAACACGTTCTTTACAGGGAACTTTGTTGGGTTGAATTTTCCAATTTGTTGCAGCCA

>5474543

ATGACCAAGGAATAAGGGTTCGTTTTTAAATAATTCTATATATTTTTAAAATAAGTAAGTACTTATGTTGTATTGATAATCGACATCAAATACAAAAAATATTTAATATTATCATTATATTTTTAAAATAAGTAAGTACTTATGTTGTTTTGATAATCGGCAATCAGACTCATGTCTGTCGCAAACTTGGCGCTCTCCCCCATTTCGCTCTTTATCTCGGCTCTCTTTTGAGTTTTATTTGGTAAACAA

>5941215

ATGTACCCTCTGGGAGCCAATATGCTATATGACCTGCGATATAGTTTCAGTAGCTGCTCGGATGTAAAATTGGCACAAGTGATGTAGTTAATCAAGGACCAAGGAATAAGAGGTTCGTTTTTAAATAATTCTATATATTTTTAAAATAAGTAAGTACTTATGTTGTTTTGATAATCGACAATCAAATTCAAAAAATATTTAATATTAATTAGCGTTTATTGTGTTTACAAATAACA

>595264697

ATGTATATTTATTAGCAATTGTTTTAAATTTCATGTTGCTAGTTAATTGACGCTAATTAATATTAAATATTTTTTGATTTTGATTGTCGATTACCGATTGAGAATTGTACAATTGCGTATTTTTGATTACACCCACTGTTGTTGCAAATATTTTGATAGCGAACTAAAACTAATTGGGCGAATCCCAAGGCAAGCGT

>6084262

ATGTTGTTTTGAAAATCGACAATCAAATTCAAAAAATATTTAATATTATCGTTATATTTTTAAAATAAGTAAATACTTCTGTTGTTTTAATAATCGGCAATCAAACTCAAAAAATATTTAATATTAATTAGCGTTTATTGTGTTTACAAATAACACTAAACTTGTAATGGTTTTGTTTCATTTTCATGTACCCTCTGGGAGCCAATAAGCTAGATGACCTGCGATGTAGTTTCAGAAGCTGCGCTGATGC

>6085466

ATGTTTACAATGCTGGAACAAATTTAAAAAACAAAACGTTAAGGTATGAACCATTTTGTTATAATTGATAATTAAAATGAAAAATGCAGATTTGTTTAGAGCTAGAACATACCATTCTCTAGGTTTGAGTGTCACAATACTAAATCAAGTTTAAAACACAATCGTTTAGCATGCACCAATTTGCTTTAAGTGATTAACTGCAACGCTGATAATTATCCACCAATTTGCTTTGAATGATGTACTGCAACG

>6089682

ATGTTTGAGTTTCACAATACTAAAACTACTTTAAAACACAATCGTTTAGCATGCAACAATTTGCTTTGAGTGATTAACTGCAACGCTGCTAACTATCCACCAATTTGCTTCGTGTGATGCACTGCAACGCTGATAATGACGGTTTGATGCCATGGCTGACGGGCAGCCACTTTAAGATTGCGCAAAAGTCCCTGGAGGCGTTCTCGGAGCTAATAAAGCGACTGGGCAGCGACTTCAATGCATACACG

>6109993

ATTATCGTTATAATAAATAGTAAGTACTTATGTTGTTTTGATAATCGGCAATCAGACTCAAAAAATATTTAATATTAATTAGCGTTTATTGTGTTTACAAATAACACTAAACTTGTAATGGTTTTGTTTTAATTACATGTACCCTCTGGGAGCCAATAAGCTAGATGACCTGCGATGTAGTTTCAGTAGCTGCTCGGATGTAAAATCAGCACAAATGATGTAGTTAATCAAGGACCAAGGAAAAGGAGGG

>6156233 rev

ATTCGATATTGTCAATATTTTTATGATCGGAGGTCATCTAGCTTATTGGCTCCCAGAGGGTACATGTAATTAAAACAAAACCATTACAAGTTTAGTGTTATTTGTAAACACAATAAACGCTAATTAATATTAAATATTTGTTGAGTCTGATTGCCAAATATCAAAACAACATAAGTACTTACTATTTATTATAACGATAATATTAAATATTTTTTGAATTTGATTTTCGATTATCAAAACAACATAA

>6270409

ATTTTCTGAGTCTGATTGCCGATTATCAAAACAACATAAGTACTTACTTATTTTAAAAATATAACGATAATATTAAATATTTTTTGAATTTGACAACATAAGTACTAACATATTTTAAAAATATATAGAATTAGTTAAAAACGAACCTCCTTTTCCTTGGTCCTTGATTAACTACATCATTTGTGCTAATTTTACATCCGAGCAGCTACTGAAACTATACGTGGGCGTTGGCGTGAAAATTCTG

>6361867 rev

CCTCCACTCCCGCAGCTCACCTCGGATTCCTGCCAACGACATCCTTTGTTCTGATTTTGCATCAGCGCAGCTCCTGAAACTACATCGCAGGTCATCTAGCTTATTGGCTCCCAGAGGGTACATGTAAATAAAACAAAACCATTACAAGTTTAGTGTTATTTGTAAACACAATAAACGATAATTAATATTAATATTTTCTGAGTCTGATTGCCGATTATCAAAACAACATAAGTACTTACTTATTTTAAAA

>6405472

CGATAAACATTCAAGATTATTTGACTTTGTGATAATGGGTTTCCGAATAGTTGTATGTTGTTTCATAGGCATTTACGAGGGTGCTATCAGCGCCCAATCCTACATACATCACGCTCAAGAACTCTTGGCATCTGGTCACAGTGACGCTGCATAATTCATCGGGGTCAAAGTCAAGAGCTTGGAATTTGATTGTCGATTATCAAAACAACATAAGTACTTACTTATTTTAAAAATATAACGATAATATTA

>6769127

TAAGTAAGTACTTATGTTGTTTTGATAATCGGCAATCAGACTCAGAAAATATTAATATTAATTATCGTTTATTGTGTTTACAAATAACACTAAACTTGTAATGGTTTTGTTTTAATTACATGTACCCTCTGGGAGCCAATAATCTAGATGACCTGCGATGTAGTTTCAGGAGCTGCGCTGATGCAAAATCAGAACAAAGGATGTCGTAATTCAAGGACCATAGAAAAAGAGGTTTGTATAGGACAAAAGC

>6769129

TAAGTAAGTACTTATTGTTTTGATAATCGGCAATCAAATTCAAAAAATATTTAATATTAATTAGCGTTTATTGTGTTTACAAATAACACTAAACTTGTAATGGTTTTGTTTTAATTACATGTACCCTCTGGGAGCCAATAAGCTAGATGGCCTGCGATACAGTTTCAGGAGCTGCTTGGATGCAAAATCAGAACAAAGGATGTCGTAAATCAAGGACCAAAGAATACGATGATCGTATCTAAATAATT

>6787007 rev

TACAAGTTTAGTGTTATTTGTAAACACAATAAACGCTAATTAATATTAAATATTTGTTGAGTCTGATTGCCAAATATCAAAACAACATAAGTACTTACTATTTATTATAACGATAATATTAAATATTTTTTGAATTTGATTGTCGATTATCAAAACAACATAAGTACTTACTTTACCTTTTTTTTCTTGTCCGATGCCTCCAAACAAATAGCATCAACCAAAAAGCGAGCGCCATTGAAGTGTTGACCT

>7001362

TATGCTTTTGTGAAAGTTTTAATGGTTTAGTTTCAATTATAAGTTCCCTCTGGGAGCCAATACGCTAGATGACCCTCGATTTAGTTTCAGGAGCTGCGCGGATGTAAAATTAGCACAAAGGATGTCGTAAATCAAGGACCAAAGAATAAGAGGTTCGTATCCAAATAATTCGTTATATTTTTAAAATAAGTTCTTATGTTGTTTTGATAATCGACAATCAAAATCAAAAAATATTTAATATTAATTAGCG

>7206364

TCGATATATTTTTAAAATAAGTAAGTACTTATGTTGTTTTGATAATCGACAATCAAATTCCAAAAATATTTAATATTATCGTTATATATATATTGTGTTTACAAATAACACTAAACATGTAATGGTTTTGTTTTAATTAAATGTACCCTCTGGGAGCCAATACGAGTATATAGCGGTTCTGCTCCGCCCCCGCCGCGCATAAACACACACTATGGGGGAGCAGAAACAATTTGTAGGTGTGGCCGGAGTG

>7474011

TGAGTCTGATTGCCGATTATCAAAACAACATAAGTACTTACTTATTTTAAAAATATAATGATAATATTAAATATTTTTTGTATTTGATGTCGATTATCAATACAACATAAGTACTTACTTATTTTAAATAGGGTGAGCTCTTCATCGTCTGCGTCTTGATGATTTGATCGTCCAGCAGTATTTGGATGTCATCCACGGCAGCCAGCTTGAATGTGCCGGAATCTCTGCGGGAGGCATTTCAAT

>7548560

TGCACTTCCTCTTGCATGGCAACCCCCTTGCCCTTTGCCCCTTGAACCCCCAACTCCCCCAACAATGGGTTGCCATTGCCAATTTTCCTCATTCTTGGACTCTTGCTGCTGATTGTGGGCATGGAGATGCACGGATAAATAGCCACGGCATTATGTTATTGTATCTCGGTGCTCCATCGTTGTAATCATCACAAGTTGATTTTTTGAGTCTGATTGCCGATTATCAAAACAACATAAGTACTTACTTAT

>8081618

TGTGCTAATTTTACATCCGAGCAGCTCCTTAAACTATATCGCAGGTCATATAGCTTATTGGCTCCCAGAGGGTACATTTAATTAAAACAAAACCATTACATGTTTAGTGTTATTTGTAAACACAATATATATATAACGATAATATTAAATATTTTTGGAATTTGATTGTCGATTATCAAAACAACATAAGTACTTACTTATTTTAAAAATATATCG

>8140913

TGTTATTTGTAAACACAATAAACGCTAATTAATATTAAATATTTTTTGAATTTGATTGCCGATTATCAAAACAATAAGTACTTACTTATTTTAAAAATAACGATAATATTAAATATTTTTTGAATTTGATTGTCGATTTTCAAAACAACATAAGAACTTATTTTAGAAATATAACGAATTATTTAGATACGAACATCTTATTCTTTGGTCCTTGATTTACGACATCCTTTGTTCTGATTTTGCATCAGCG

>8388730 rev

TTGTTCCAGCATTGTAAACATCAAGCCTAGAGAATTCTAGTTCTAGCACTAAACAAATCTTCAATTTCATAAAGCAACAACCAATTGCTTACCATAAGCATTATATTTTGTTACGTTTTTCATAATTATCAGCGTTGCAGTGCATCACTCAAAGCAAATTGGTACATGCTAAACGATTGTGTTTTAAATTTGTTTTAGTATTGTGAAACTCAAACTTAGAGAA
