# Supplementary material for: Simple and Complex Centromeric Satellites in Drosophila Sibling Species
Source: Genetics. 2018 Jan 5;208(3):977–90. doi: 10.1534/genetics.117.300620 (PMC5844345; doi:10.1534/genetics.117.300620)
Supplement: Supplementary file 9 [file 977FileS5.docx]

**File S5. Alignment of *simcent1* reference sequences.** Colored text and shading mark homologous subregions. 10mers used in counting are underlined.

7548560 ------------------------------------------------------------

1836793 ------------------------------------------------------------

151978 ------------------------------------------------------------

7474011 ------------------------------------------------------------

5474543 ------------------------------------------------------------

6787007 ------------------------------------------------------------

5125404 ------------------------------------------------------------

8140913 ------------------------------------------------------------

311304 ------------------------------------------------------------

4980959 ------------------------------------------------------------

6156233 ------------------------------------------------------------

3580903 ------------------------------------------------------------

1108768 ------------------------------------------------------------

1302316 ------------------------------------------------------------

6405472 ------------------------------------------------------------

5288992 ------------------------------------------------------------

2785757 AGAGAAAAGGTAGCAAAAAAAAAAGTAGA--------------------------TATAA

2244836 ------------------------------------------------------------

4301688 ------------------------------------------------------------

988140 ------------------------------------------------------------

2400817 ------------------------------------------------------------

6084262 ------------------------------------------------------------

7206364 ------------------------------------------------------------

2576612 ------------------------------------------------------------

6109993 ------------------------------------------------------------

6769129 ------------------------------------------------------------

6769127 ------------------------------------------------------------

6361867 ------------------------------------------------------------

529887 ------------------------------------------------------------

8081618 ------------------------------------------------------------

4270451 ------------------------------------------------------------

2066977 ------------------------------------------------------------

5952646 ------------------------------------------------------------

6270409 ------------------------------------------------------------

1923314 ------------------------------------------------------------

151837 ------------------------------------------------------------

1920360 ------------------------------------------------------------

5941215 ------------------------------------------------------------

1265444 ------------------------------------------------------------

205311 ------------------------------------------------------------

539283 ------------------------------------------------------------

2447119 ------------------------------------------------------------

7001362 ------------------------------------------------------------

4714083 ------------------------------------------------------------

5287660 ---------------------------------ATCGAATTTTTAAAATAAAATACCGAT

1297993 ACAAAAAATTTACTTCATAAAAATATTGACAATATCGAATTTTTAAAATAAAATACCAAT

7548560 ------------------------------------------------------------

1836793 ------------------------------------------------------------

151978 ------------------------------------------------------------

7474011 ------------------------------------------------------ATTGAA

5474543 ------------------------------------------------------------

6787007 ------------------------------------------------------------

5125404 ------------------------------------------------------------

8140913 ------------------------------------------------------------

311304 ------------------------------------------------------------

4980959 ------------------------------------------------------------

6156233 ------------------------------------------------------------

3580903 ------------------------------------------------------------

1108768 ------------------------------------------------------------

1302316 ------------------------------------------------------------

6405472 ------------------------------------------------------------

5288992 ------------------------------------------------------------

2785757 GAGAGAAAAATATATTTCGCCTGC------------------------------------

2244836 ------------------------------------------------------------

4301688 ------------------------------------------------------------

988140 ------------------------------------------------------------

2400817 ------------------------------------------------------------

6084262 ------------------------------------------------------------

7206364 ------------------------------------------------------------

2576612 ------------------------------------------------------------

6109993 ------------------------------------------------------------

6769129 ------------------------------------------------------------

6769127 ------------------------------------------------------------

6361867 ------------------------------------------------------------

529887 ------------------------------------------------------------

8081618 ------------------------------------------------------------

4270451 ------------------------------------------------------------

2066977 ------------------------------------------------------------

5952646 ------------------------------------------------------------

6270409 ------------------------------------------------------------

1923314 ------------------------------------------------------------

151837 ------------------------------------------------------------

1920360 ------------------------------------------------------------

5941215 ------------------------------------------------------------

1265444 ------------------------------------------------------------

205311 ------------------------------------------------------------

539283 ------------------------------------------------------------

2447119 ------------------------------------------------------------

7001362 ------------------------------------------------------------

4714083 ------------------------------------------------------------

5287660 TCGTTGAAAATATATGTCCCAGGGATGGCATTCATACATTGATGACGTGGTCCATTTGAC

1297993 TCGTTGAAAATATATGTCCCAGGAATGGCATTCATACATTGATGACGTGGTCCATTTGAC

7548560 ------------------------------------------------------------

1836793 ------------------------------------------------------------

151978 ------------------------CCGTTCCTTTTCCATTCAATGGCA------------

7474011 ATG-----------------CCTCCCGCAGAGATTCCGGCACATTCAA------------

5474543 ------------------------------------------------------------

6787007 --------------------------------------------AGGT------------

5125404 ------------------------------------------------------------

8140913 -------------------------------------CGCTGATGCAA------------

311304 ------------------------------------------------------------

4980959 ------------------------------------------------------------

6156233 ------------------------------------------------------------

3580903 ------------------------------------------------------------

1108768 ------------------------------------------------------------

1302316 ------------------------------------------------------------

6405472 ------------------------------------------------------------

5288992 ------------------------------------------------------------

2785757 ------------------------------------------------------------

2244836 ------------------------------------------------------------

4301688 ------------------------------------------------------------

988140 ------------------------------------------------------------

2400817 ------------------------------------------------------------

6084262 ------------------------------------------------------------

7206364 ------------------------------------------------------------

2576612 ------------------------------------------------------------

6109993 ------------------------------------------------------------

6769129 ------------------------------------------------------------

6769127 ------------------------------------------------------------

6361867 ------------------------------------------------------------

529887 ------------------------------------------------------------

8081618 ------------------------------------------------------------

4270451 ------------------------------------------------------------

2066977 ------------------------------------------------------------

5952646 ------------------------------------------------------------

6270409 ------------------------------------------------------------

1923314 ------------------------------------------------------------

151837 ------------------------------------------------------------

1920360 ------------------------------------------------------------

5941215 ------------------------------------------------------------

1265444 ------------------------------------------------------------

205311 ----------------------------------------------AAACTTATGCTTAT

539283 ------------------------------------------------------------

2447119 ------------------------------------------------------------

7001362 ------------------------------------------------------------

4714083 --------------AGTGTTGGTCACGTGATCGTTAATGCTAATTAAAACTTATGCTTAT

5287660 ATTAAATCAGTTATAGTGTTGGTCACGTGATCGTTAATGCTAATTAAAACTTATGCTTAT

1297993 ATTAAATCAGTTAAAGTGTTGGTCACGTGATCGTTAATGCTAATTAAAACTTATGCTTAT

7548560 ------------------------------------------------------------

1836793 ------------------------CCGCTTAGGA--GCCACAC--ATGTTTCAAGCTCA-

151978 ----------------CCTCGA-----TATT----------------TTTCCTTGAGCA-

7474011 ----------------GCTGGCTGCCGTGGATGA--CATCCAA--ATACTGCTGGACGA-

5474543 ----------------------------------------------------ATGACCA-

6787007 ----------------CAACACTTCAATGGCG------------------CTCGCTTTT-

5125404 ------------------------------------------------ATCAAGGACCA-

8140913 ----------------AATCAGAACAAAGGATGT--CGTA-------AATCAAGGACCA-

311304 ------------------------------------------------------------

4980959 ------------------------------------------------------------

6156233 ------------------------------------------------------------

3580903 ------------------------------------------------------------

1108768 -----------------------------------------------AATCAAGGACCA-

1302316 ------------------------------ATGT--AGTT-------AATCAAGGACCA-

6405472 ------------------------------------------------------------

5288992 ------------------------------------------------------------

2785757 ------------------------------------------------------------

2244836 ------------------------------------------------------------

4301688 ------------------------------------------------------------

988140 ------------------------------------------------------------

2400817 ------------------------------------------------------------

6084262 ------------------------------------------------------------

7206364 ------------------------------------------------------------

2576612 ------------------------------------------------------------

6109993 ------------------------------------------------------------

6769129 ------------------------------------------------------------

6769127 ------------------------------------------------------------

6361867 ------------------------------------------------------------

529887 ------------------------------------------------------------

8081618 ------------------------------------------------------------

4270451 ------------------------------------------------------------

2066977 ------------------------------------------------------ACGGAA

5952646 -----------------------------------------ACGCTTGCCTTGGGATTC-

6270409 -------------------------------------------------------CAGAA

1923314 ------------------------------------------------------------

151837 ------------------------------------------------------------

1920360 ------------------------------------------------------------

5941215 -----------------------------------------ATGTACCCTCTGGGAGCCA

1265444 ---------------------AATGTTTTTGTTTTAATTACACGTACCTTTTTGGAGCCA

205311 TGTACATAAAACTAAACTTGTAATGGTTTTGTTTTAATTT--CTTTTACCCTGGGAGCCA

539283 ----TATAACACTAAACTTGTAATGGTTTTGTTTTAATTACATGTACCCTCTGGGAGCCA

2447119 ---------------ACTTGTAATGGTTTTGTTTTAATTTCATGTATCCTCTGGGAGCCA

7001362 -TATGCTTTTGTGAAAGTTTTAATGGTTTAGTTTCAATTATAAGTTCCCTCTGGGAGCCA

4714083 TGTACATAAAACTAAACTTGTAATGGTTTTGTTTTAATTTCTTGTACCCT--GGGAGCCA

5287660 TGTACATAAAACTAAACTTGTAATGGTTTTGTTTTAATTTCTT--GTACCCTGGGAGCCA

1297993 TGTACATAAAACTAAACTTGTAATGGTTTTGTTTTGCACACGA--CTAAATATGCCAATT

7548560 ------------------------------------------------------------

1836793 ---TGTGGCCAGCAGCTTCT----TAAACTGATCCGCCT----CCAACTTTTGTT---TG

151978 ---GAGA---------------------------AAGCTGTGGGGAAAAGATTTCAGATT

7474011 ---TCAAATCATCAAGACGCAGACGATGAAGAGCTCACCCTATTTAAAATAAGTAAGTAC

5474543 ---AGGAAT--AAGGGTTCGTTTTTAAATAATTCTATATATTTTTAAAATAAGTAAGTAC

6787007 ---TGGTTGATGCTATTTGTTTGGAGGCATCGGACAAGAAAAAAAAGGTAAAGTAAGTAC

5125404 ---AGGAATAAGAGCTTCGTTTT-TAAATAATTCTATATATTATTAAAATAAGTAAGTAC

8140913 ---AAGAATAAGATGTTCGTATC-TAAATAATTCGTTATATTTCTAA----AATAAGTTC

311304 ------------------------------------------------------------

4980959 ------------------------------------------------------------

6156233 ------------------------------------------------------------

3580903 -------------------TTTT-TAAATAATTCTATATATTTTTAAAATAAGTAAGTAC

1108768 ---AGGTATAAGAGTTTCGTTTT-TAAATAATTCTATATATTATAAAAATAAGTAAGTAC

1302316 ---AGGAATAAGAGGTTCGTTTT-TAAATAATTCTATATATTATTAAAATAAGTAAGTAC

6405472 ------------------------------------------------------------

5288992 ------------------------------------------------------------

2785757 --------------------------------------------------GAGGCAGAAA

2244836 ----------------------------------------------------------AC

4301688 ------------------------------------------------------------

988140 --------------------------------------------------AAGTAAGTAC

2400817 ----------------------------------------------------------AC

6084262 ------------------------------------------------------------

7206364 ------------------------------------------------------------

2576612 ------------------------------------------------------------

6109993 ------------------------------------------------------------

6769129 ------------------------------------------------------------

6769127 ------------------------------------------------------------

6361867 ------------------------------------------------------------

529887 ------------------------------------------------------------

8081618 ------------------------------------------------------------

4270451 ---------------------------------------------------AGTAAGTAC

2066977 ATTCGGCTGCTCAGCTGGCAA-----ATGATGATGTTCGGGG--------TGGCCAA---

5952646 ---------------GCCCAATTAGTTTTAGTTCG----CTATCAAAATATTTGCAA---

6270409 TTTTCACGCCAACGCCCACGTATAGTTTCAGTAGCTGCTCGGATGTAAAATTAGCACAAA

1923314 -----------------------------AGGAGATGCTCGGATGTAAAATTAGCACAAA

151837 -------------------------------GAGCTTCGCGGATGTAAAATTAGCACAAA

1920360 ------------ACCTACGATGTAAGTTTAGGAGCTATGCGAATGTACAATCAGCGAATA

5941215 ATATGCTATATGACCTGCGATATAGTTTCAGTAGCTGCTCGGATGTAAAATTGGCACAAG

1265444 ATAAGCTAGATGACCTGCGATGTAGTTTCAGGAGCTGCGCGGATGTAAAATTAGAAC-AA

205311 ATAAGATAGATGACCTGCGATGTAGTTTCAGGATCTGCGCGGATGAAAAATTAACACAAA

539283 ATAAGCTAGATGACCTGCGATGTAGTTTCAGGAGCTGCGCTGATGCAAAATCAGAACAAA

2447119 ATAAGCTAGATGACCTGTGATGTAGATTCAGGATCTGCGCTGATGTAAAATCAGAACAAA

7001362 ATACGCTAGATGACCCTCGATTTAGTTTCAGGAGCTGCGCGGATGTAAAATTAGCACAAA

4714083 ATAAGATAGATGACCTGCGATGTAGTTTCAGGATCTGCGCGGATGAAAAATTAACACAAA

5287660 ATAAGATAGATGACCTGCGATGTAGTTTCAGGATCTGCGCGGATG---------------

1297993 TAAACAAAAA--------------------------------------------------

7548560 ------------------------------------------------------------

1836793 CTGGTTCAGGGTCCGATTTGTGGATCTGTTTTCGCGTTTTTGGCGGCACTTGCTTTCTAT

151978 CTCAGATTTCGTTGCTTTGTATTGTTGTTAACCTGCTCTTTTTTCGGTGATCGTTATATT

7474011 TTATGTTGTATTGATAATCGACA-TCAAATACAAAAAATATTTAATATTATCATTATATT

5474543 TTATGTTGTATTGATAATCGACA-TCAAATACAAAAAATATTTAATATTATCATTATATT

6787007 TTATGTTGTTTTGATAATCGACAATCAAATTCAAAAAATATTTAATATTATCGTTATAA-

5125404 TTATGTTGTTTTGATAATCGACAATCAAATTCAAAAAATATTTAATATTATCGTTATAA-

8140913 TTATGTTGTTTTGAAAATCGACAATCAAATTCAAAAAATATTTAATATTATCGTTATTTT

311304 ------------------------------------------------------------

4980959 -----------------------------------------------------TATCGTT

6156233 TTATGTTGTTTTGATAATCGAAAATCAAATTCAAAAAATATTTAATATTATCGTTATA--

3580903 TTATGTTGTTTTGATAATCGACAATCAAATTCAAAAAATATTTAATATTATCGTTATA--

1108768 TTATGTTGTTTTGATAATCGACAGTCAAATTCAAAAAATATTTAATATTATCGTTATATT

1302316 TTATGTTGTTTTGATAATCGACAATCAAATTCAAAAAAAATTTAATATTATCGTTATA--

6405472 ------------------------------------------TAATATTATCGTTATATT

5288992 -----------------ATCGACATCAAATACAAAAAATATTTAATATTATCATTATATT

2785757 TTCCGATGCC-ATCAAGGACCATAGAAAAAGAGGTTTGTATCTAAATAATTCGATATATT

2244836 TCCGCCAGCAAGTCGTACTGATC----ATCGTCCGCACACCACCACACCATCATTATATT

4301688 ------------------------------------------------------------

988140 TTATGTTGTT-TTGATAATCGACATCAAATACAAAAAATATTTAATATTATCATTATATT

2400817 TTATGTTGTTTTG-ATAATCGACATCAAATACAAAAAATATTTAATATTATCATTATATT

6084262 --ATGTTGTTTTGAAAATCGACAATCAAATTCAAAAAATATTTAATATTATCGTTATATT

7206364 --------------------------------------------------TCGATATATT

2576612 ---------------------------------------------------------AGT

6109993 ---------------------------------------------------ATTATCGTT

6769129 ------------------------------------------------------------

6769127 ------------------------------------------------------------

6361867 -----------------------------------------------------------T

529887 -------------------------ATAGAGACTTTCAATTTATTCGGGCTATCGTTATT

8081618 ---------------------------------------------------CGATATATT

4270451 TTATGTTGTTTTGATAATCGA-CATCAAATACAAGAAATATTTAGTATTATCGTTATATT

2066977 -AAAGTCAAGTCAGCAGCACAAATGTGTGTGTGTGAATTGTGACCCAATATCGTTATATT

5952646 --------------------------------------------CAACAGTGGGTGTAAT

6270409 TGATGTAGTTAATCAAGGACCAAGGAAAAGGAGGTTCGTTTTTAACTAATTCTATATATT

1923314 TGATTTCGTAAATCAAGGACCAAAGAATAAGAAGTTCGTATTTAAATAATTCGTTATATT

151837 TGATGTCGTTAATCAAGGACCAAGGAATAAAAGGTTTGTATCTAAATAATTCGTTATATT

1920360 GGATGTCATAAATCAAGGACCATAGAATAAGAAGTTTGTATCTAAATAATTCGATATATT

5941215 TGATGTAGTTAATCAAGGACCAAGGAATAAGAGGTTCGTTTTTAAATAATTCTATATATT

1265444 GGCTGTCGTAAATCAAGGACCAAAGAATAAGAGGTTCGTAACTAAATAATTCGTTATAAT

205311 GGATGTCTTAAATTAAGGACCAAAGTATAAGAGGTTCGTATCTAAATAATTCGTTATAAT

539283 GGATGTCGTAATTCAAGGACCATAGAAAAAGAGGTTTGTATCTAAATAATTCGATATATT

2447119 GGATGTCGTAAATCAAGGACCAAAGAAAAGGAGGTTCGTATCTAAATAATTCGTTATATT

7001362 GGATGTCGTAAATCAAGGACCAAAGAATAAGAGGTTCGTATCCAAATAATTCGTTATATT

4714083 GGATGTCTTAAATTAAGGACCAAAGTATAAGAGGTTCGTTGCATGGCAACTGCACATTAC

5287660 ------------------------------------------------------------

1297993 ------------------------------------------------------------

7548560 ------ATAAGTAAGTACTTATGTTGTTTTGATAATCGGCAATCAGACTCAAAAAATCAA

1836793 TTTAAAATAAGTAAGTACTTATGTTGTTTTGATAATCGGCAATCAGACTCAAAAAATATT

151978 TTTAAAATAAGTAAGTACTTATGTTGTTTTGATAATCGGCAATCAGACTCAGAAAATATT

7474011 TTTAAAATAAGTAAGTACTTATGTTGTTTTGATAATCGGCAATCAGACTCA---------

5474543 TTTAAAATAAGTAAGTACTTATGTTGTTTTGATAATCGGCAATCAGACTCATGTCTGTCG

6787007 ----TAAATAGTAAGTACTTATGTTGTTTTGATATTTGGCAATCAGACTCAACAAATATT

5125404 ----TAAATAGTAAGTACTTATGTTGTTTTGATATTTGGCAATCAGACTCATAGTCAACC

8140913 TAAAATAA----GTAAGTACTTATTGTTTTGATAATCGGCAATCAAATTCAAAAAATATT

311304 -----AAATAGTAAGTACTTATGTTGTTTTGATATTTGGCAATCAGACTCAACAAATATT

4980959 ATAATAAATAGTAAGTACTTATGTTGTTTTGATAATCGGCAATCAGACTCAAAAAATATT

6156233 ---ATAAATAGTAAGTACTTATGTTGTTTTGATATTTGGCAATCAGACTCAACAAATATT

3580903 ---ATAAATAGTAAGTACTTATGTTGTTTTGATAATCGGCAATCAGACTCAAAAAATATT

1108768 TTTAAAATAAGTAAGTACTTATGTTGTTTTGATAATCGGCAATCAGACTCAAAAAATATT

1302316 ---ATAAATAGTAAGTACTTATGTTGTTTTGATAATCGGCAATCAGACTCAACAAATATT

6405472 TTTAAAATAAGTAAGTACTTATGTTGTTTTGATAATCGACAATCAAATTCCAAGCTCTTG

5288992 TTTAAAATAAGTAAGTACTTATGTTGTTTTGATAATCGGCAATCAGACTCAAAAAATATT

2785757 TTTAAAATAAGTAAGTACTTATGTTGTTTTGATAATCGACAATCAAATTCCAAAAATATT

2244836 TTTAAAATAAGTAAGTACTTATGTTGTTTTGATAATCGGCAATCAGACTCAAAAAATATT

4301688 -------------AGTACTTATGTTGTTTTGATAATCGGCAATCAGACTCAAAAAATATT

988140 TTTAAAATAAGTAAGTACTTATGTTGTTTTGATAATCGGCAATCAGACTCAAAAAATAT-

2400817 TTTAAAATAAGTAAGTACTTATGTTGTTTTGATAATCGGCAATCAGACTCAAAAAATATT

6084262 TTTAAAATAAGTAAATACTTCTGTTGTTTTAATAATCGGCAATCAAACTCAAAAAATATT

7206364 TTTAAAATAAGTAAGTACTTATGTTGTTTTGATAATCGACAATCAAATTCCAAAAATATT

2576612 ATTAAAATAAGTAAGTACTTATGTTGTTTTGATA--------------------------

6109993 ATAATAAATAGTAAGTACTTATGTTGTTTTGATAATCGGCAATCAGACTCAAAAAATATT

6769129 -------TAAGTAAGT--ACTTATTGTTTTGATAATCGGCAATCAAATTCAAAAAATATT

6769127 -------TAAGTAAGTACTTATGTTGTTTTGATAATCGGCAATCAGACTCAGAAAATAT-

6361867 TTTAAAATAAGTAAGTACTTATGTTGTTTTGATAATCGGCAATCAGACTCAGAAAATAT-

529887 TTTAAAATAAGTAAGTAC--TTATTGTTTTGATAATCGGCAATCAAACTCAAAAAATATT

8081618 TTTAAAATAAGTAAGTACTTATGTTGTTTTGATAATCGACAATCAAATTCCAAAAATATT

4270451 TTTAAAATAAGTAAGTACTTATGTTGTTTTGATAATCGACAATCAAATTCCAAAAATATT

2066977 TTTAAAATAAGTAAGTACTTATGTTGTTTTGATAATCGACAATCAAATTCCAAAAATATT

5952646 CAAAAATACGCAATTGTACAATTCTCAATCGGTAATCGACAATCAAAATCAAAAAATATT

6270409 TTTAAAATATGTTAGTACTTATGTTGTCAAATTCAAAAAATATTTAATATTATCGTTATA

1923314 TTAAAAATAAGTAAGTACTTATGTTGTTTTGATAATCGACAATCAAATTCAAAAAATATT

151837 TTTAAAATAAGTAAGTACTTATGTTGTTTTGATAATCGACGATCAAAATCAAAAAATATT

1920360 TTTAAAATAAG----TTCTTATGTTGTTTTGATAATCGACAATCAAAATCAAAAAATATT

5941215 TTTAAAATAAGTAAGTACTTATGTTGTTTTGATAATCGACAATCAAATTCAAAAAATATT

1265444 TTTAAAATAAGTAAGTACTTATGTTGTTTTGATAATCGGCAATCAAACTAAAAAAATTTA

205311 TTTAAAATAAGTAAGTACTTATGTTGTTTTGATAATCGGCAATCAAACTAAAAAAAAA--

539283 TTTAAAATAAGTAAGTACTTATGTTGTTTTGATAATCGACAATCAAATTCCAAAAATATT

2447119 TCTAAAATAAG----TTCTTATGTTGTTTTGATAATCGACAATCAAAATCAAAAAATATT

7001362 TTTAAAATAAG----TTCTTATGTTGTTTTGATAATCGACAATCAAAATCAAAAAATATT

4714083 CGTTTCGTACTTTTTCATGCATTCA-----------------------------------

5287660 ------------------------------------------------------------

1297993 ------------------------------------------------------------

7548560 CTTGTG---ATGATTACAACGATGGAGCA-CCGAGATACAATAACATAATGCCG------

1836793 TAATAT---TAATTAGCGTTTATTGTGTTTACAAATAACACTAAACTTGTAATGGT----

151978 -AATAT---TAATTATCGTTTATTGTGTTTACAAATAACACTAAACTTGTAATGGTTT--

7474011 ------------------------------------------------------------

5474543 CAAACT---TGGCGCTCTCCCCCATTTCGCTCTTTA-TCTCGGCTCTCTTTTGAGTTTTA

6787007 TAATAT---TAATTAGCGTTTATTGTGTTTACAAATAACACTAAACTTGTA---------

5125404 TCCATT---GGTTCCGACCTTTGATGTTGTGGATCT-----TGACTTTGGAACCGTTGTC

8140913 TAATAT---TAATTAGCGTTTATTGTGTTTACAAATAACA--------------------

311304 TAATAT---TAATTAGCGTTTATTGTGTTTACAAATAACACTAAACTTGTAATGGTTTTG

4980959 TAATAT---TAATTAGCGTTTATTGTGTTTACAAATAACACTAAACTTGTAATGGTTTTG

6156233 TAATAT---TAATTAGCGTTTATTGTGTTTACAAATAACACTAAACTTGTAATGGTTTTG

3580903 TAATAT---TAATTAGCGTTTATTGTGTTTAAAAATAACACTAAACTTGTAATGGTTTTG

1108768 TAATAT---TAATTAGCGTTTATTGTGTTTACAAATAACACTAAACTAGTAATAGTTTTT

1302316 TAATAT---TAATTAGCGTTTATTGTGTTTACAAATAACACTAAACTTGTAATGGTTTTG

6405472 ACTTTGACCCCGATGAATTATGCAGCGTCACT--GTGACCAGATGCCAAGA---GTTCT-

5288992 TAATAT---TAATTAGCGTTATGTGCTTCA---AGAGAAATGTTTTTTTCTAGGACTGGTTTG

2785757 TAATATTATCGTTATATATATATTGTGTTTACAAATAACACTAAACATGTAATGGTTTTG

2244836 TAATAT---TAATTAGCGTTTATTGTGTTTACAAATAACACTAAACTTGTAATGGTTTTG

4301688 TAATAT---TAATTAGCGTTTATTGTGTTTACAAATAACACTAAACTTGTAATGGTTTTG

988140 ---------------------------------------TCTAAACTTGTAATGGTTTTG

2400817 TAATAT---TAATTAGCGTTTATTGTGTTTACAAATAACACTAAACTTGTAATGGTTTTG

6084262 TAATAT---TAATTAGCGTTTATTGTGTTTACAAATAACACTAAACTTGTAATGGTTTTG

7206364 TAATATTATCGTTATATATATATTGTGTTTACAAATAACACTAAACATGTAATGGTTTTG

2576612 --------------ATCGGCAATCAGACTCAAAAAATATTCTAAACTTGTAATGGTTTTG

6109993 TAATAT---TAATTAGCGTTTATTGTGTTTACAAATAACACTAAACTTGTAATGGTTTTG

6769129 TAATAT---TAATTAGCGTTTATTGTGTTTACAAATAACACTAAACTTGTAATGGTTTTG

6769127 TAATAT---TAATTATCGTTTATTGTGTTTACAAATAACACTAAACTTGTAATGGTTTTG

6361867 TAATAT---TAATTATCGTTTATTGTGTTTACAAATAACACTAAACTTGTAATGGTTTTG

529887 TAATAT---TAATTAGCGTTTATTGTGTTTACAAATAACACTAAACTAGTAATAGTTTTG

8081618 TAATATTATCGTTATATATATATTGTGTTTACAAATAACACTAAACATGTAATGGTTTTG

4270451 TAATATTATCGTTATATATCAAAATCAAAAAATATTTAATAATAATTAGCAGTAAATAAG

2066977 TAATATTATCGTTATATATCAAAATCAAAAAATATTTAATATTAATTAGCAGTAAATAAG

5952646 TAATATTAATTAGCGTCAATTAAC------------------------------------

6270409 TTTTTAAAATAAGTAAGTACTTATGTTGTTTTGATAATCGGCAATCAGACTCAGAAAAT-

1923314 TAATATTATCGTTTACCGCTGTTA------------------------------------

151837 TAATAT---TAATTAGCGGTAATTAAC------------------------------------

1920360 TAATAT---TAATTAGCGTTAATTAAC------------------------------------

5941215 TAATAT---TAATTAGCGTTTATTGTG------------------------------------

1265444 A—TAT----TAATTAGCAGTTATTAAC------------------------------------

205311 ------------------------------------------------------------

539283 TAATATTATCGTT-----------------------------------------------

2447119 TAATAT---TAATTAGCGTTAATTAAC------------------------------------

7001362 TAATAT---TAATTAGCG---------------------------------------------

4714083 ------------------------------------------------------------

5287660 ------------------------------------------------------------

1297993 ------------------------------------------------------------

7548560 TGGCTATTTATCCGTGCATCTCCATGCCCACAATCAGCAGCAAGAGTCCAAGAA-TGAGG

1836793 ------------------------------------------------------------

151978 ------------------------------------------------------------

7474011 ------------------------------------------------------------

5474543 TTTGGT------------------AAACAA------------------------------

6787007 ------------------------------------------------------------

5125404 TTGAGA--AATTAGAGTG------------------------------------------

8140913 ------------------------------------------------------------

311304 TTTTAATTACATGTACCCTCTGGGAGCCAATAAGCTAGATGACCTCCGATCATAAAAATA

4980959 TTTTAATTACATGTACCCTCTGGGAGCCAATAAGCTAGATGACCTCCGATCATAAAAATA

6156233 TTTTAATTACATGTACCCTCTGGGAGCCAATAAGCTAGATGACCTCCGATCATAAAAATA

3580903 TTTTAATTACATGTACCCTCTGGGAGCCAATAAGCT------------------------

1108768 TTTA--------------------------------------------------------

1302316 T-----------------------------------------------------------

6405472 TG--AGCGTGATGTATGTAGGATTGGGCGCTGATAGCACC---------------CTCGT

5288992 TTGGCATTTAATTTCCCTTGAAAA----AATAATTTATAACACGTTCTTTACAGGGAACT

2785757 TTG---------------------------------------------------------

2244836 TTTTAATTACATGTACCCTCTGCGAGCCAACGAAGCCCAAGCCAAACTTAACTGCGAGAA

4301688 TTTTAATTACATGTACACTCTGGGAGCCAATAAGCTAGATGGCCTGCGATGTAGTTTCAG

988140 TTTTAATTACATGTACCCTCTGCGAGCCAATAAGCTAGATGACCTCCGATGTAGTTTCAG

2400817 TTTTAATTACATGTACCCTCTGCGAGCCAATAAGCTAGATGAGCTCCGATGTAGTTTCAG

6084262 TTTCATTTTCATGTACCCTCTGGGAGCCAATAAGCTAGATGACCTGCGATGTAGTTTCAG

7206364 TTTTAATTAAATGTACCCTCTGGGAGCCAATACGAGTATATAGCGGTTCTGCTCCGCCCC

2576612 TTTTAATTACATGTACCCTCTGCGAGCCAATAAGCTAGATGACCTCCGATGTAGTTTCTG

6109993 TTTTAATTACATGTACCCTCTGGGAGCCAATAAGCTAGATGACCTGCGATGTAGTTTCAG

6769129 TTTTAATTACATGTACCCTCTGGGAGCCAATAAGCTAGATGGCCTGCGATACAGTTTCAG

6769127 TTTTAATTACATGTACCCTCTGGGAGCCAATAATCTAGATGACCTGCGATGTAGTTTCAG

6361867 TTTTATTTACATGTACCCTCTGGGAGCCAATAAGCTAGATGACCTGCGATGTAGTTTCAG

529887 TTTTAATTACATGTACCCTCTGGGAGCCAATAAGCTATATGACCTGCGATACAGTTTCAG

8081618 TTTTAATTAAATGTACCCTCTGGGAGCCAATAAGCTATATGACCTGCGATATAGTTTAAG

4270451 TAGCATTATGAAATTTAAAACAATCACTAATAGATATACA--------TGACTTAATAT-

2066977 TAGCATTATGAAATTTAAAA----------------------------------------

5952646 TAGCAACATGAAATTTAAAACAATTGCTAATAAATATACA--------T-----------

6270409 ------------------------------------------------------------

1923314 TCGTGGAACCTTCGTCCACAAAATGC-CGGTCGATAGCCA--------GGGTCACTCTCA

151837 TAGCAACATGAAATTTAAAACAGTTGCTAATAGATATACA--------TAACAAAATATG

1920360 TAGCAACATGAGGTTAGCAATCCACCATGGCACTTTGGCT--------TTGTGGATCACT

5941215 TTTACAAATAACA-----------------------------------------------

1265444 TACCACATGA--------------------------------------------------

205311 ------------------------------------------------------------

539283 ------------------------------------------------------------

2447119 TA----------------------------------------------------------

7001362 ------------------------------------------------------------

4714083 ------------------------------------------------------------

5287660 ------------------------------------------------------------

1297993 ------------------------------------------------------------

7548560 AAAATT-------GGCAATGGCAACCCATTGTTGGGGGAGTTGGGGGTTCAAGGGGCAAA

1836793 ------------------------------------------------------------

151978 ------------------------------------------------------------

7474011 ------------------------------------------------------------

5474543 ------------------------------------------------------------

6787007 ------------------------------------------------------------

5125404 ------------------------------------------------------------

8140913 ------------------------------------------------------------

311304 TTGACAATATCG---AATTTTTTAAATAAAATTACAATTCATAGGAAATCTACGTACCAG

4980959 TTGACAATATCG---AATTTTTTAAATAAAATTACAATTCATAGGAAATCTACGTACCAG

6156233 TTGACAATATCG---AAT------------------------------------------

3580903 ------------------------------------------------------------

1108768 ------------------------------------------------------------

1302316 ------------------------------------------------------------

6405472 AAATGCCTATGAAACAACATACAACTA-------------TTCGGAAACCCATTATCACA

5288992 TTGTTGGGTTGA------ATTTTCCAATTTGTTGCAGCCA--------------------

2785757 ------------------------------------------------------------

2244836 TTAATTCCCTTGTCT---------------------------------------------

4301688 TAGCTGCTCGGATGTATGCGGCTTCTTTTTTTCGTACATTTATTTTTGTGTGTTTTACTG

988140 GAGCTGCGCTGATGCAAAATCAGAACAAAGGATGTCGTAA--------------------

2400817 GAGCTGCG----------------------------------------------------

6084262 AAGCTGCGCTGATGC---------------------------------------------

7206364 CGCCGCGCATAAACACACACT----------ATGGGGGAG--CAGAAACAATTTGTAGGT

2576612 GAGCTGCGCTGATGCAAAATCAGAACAAAGGATGTCGTAATTCAAGGACCATAGAAAAGA

6109993 TAGCTGCTCGGATGTAAAATCAGCACAAATGATGTAGTTAATCAAGGACCAAGGAAAAGG

6769129 GAGCTGCTTGGATGCAAAATCAGAACAAAGGATGTCGTAAATCAAGGACCAAAGAATACG

6769127 GAGCTGCGCTGATGCAAAATCAGAACAAAGGATGTCGTAATTCAAGGACCATAGAAAAAG

6361867 GAGCTGCGCTGATGCAAAATCAGAACAAAGGATGTCGTTGGCAGGAATCCGAGGTGAGCT

529887 GAGCTGCTTGGATGTAAAATTAGCACAAATGATGTCGTT---------------------

8081618 GAGCTGCTCGGATGTAAAATTAGCACA---------------------------------

4270451 ------------------------------------------------------------

2066977 ------------------------------------------------------------

5952646 ------------------------------------------------------------

6270409 ------------------------------------------------------------

1923314 TTGGTCAGCGCATGGACAATA---------------------------------------

151837 CTACTG--TGAAAGTTTTAAT---------------------------------------

1920360 CTTTTC-------GTTT-------------------------------------------

5941215 ------------------------------------------------------------

1265444 ------------------------------------------------------------

205311 ------------------------------------------------------------

539283 ------------------------------------------------------------

2447119 ------------------------------------------------------------

7001362 ------------------------------------------------------------

4714083 ------------------------------------------------------------

5287660 ------------------------------------------------------------

1297993 ------------------------------------------------------------

7548560 GGGCAAGGGGGTTGCCATGCA----------AGAGGAAGTGCA

1836793 -------------------------------------------

151978 -------------------------------------------

7474011 -------------------------------------------

5474543 -------------------------------------------

6787007 -------------------------------------------

5125404 -------------------------------------------

8140913 -------------------------------------------

311304 AG-ATGGTAT--------------TG-----ATAATTGAT---

4980959 AG-ATGGTAT---------------------------------

6156233 -------------------------------------------

3580903 -------------------------------------------

1108768 -------------------------------------------

1302316 -------------------------------------------

6405472 AA------GTCAAATAATCTTGAATGTTTATCG----------

5288992 -------------------------------------------

2785757 -------------------------------------------

2244836 -------------------------------------------

4301688 TTGTTGTTGTCGCGAGCGTTGACAGG-----------------

988140 -------------------------------------------

2400817 -------------------------------------------

6084262 -------------------------------------------

7206364 GTGGCCGGAGTG-------------------------------

2576612 CGTTTGTATTTAAATAATTCGATATATTTTTAAAATAAGTTCT

6109993 AGGG---------------------------------------

6769129 ATGATCGTATCTAAATAATT-----------------------

6769127 AGGTTTGTATAGGACAAAAGC----------------------

6361867 GCGGGAGTGGAGG------------------------------

529887 -------------------------------------------

8081618 -------------------------------------------

4270451 -------------------------------------------

2066977 -------------------------------------------

5952646 -------------------------------------------

6270409 -------------------------------------------

1923314 ------------------------GGT----------------

151837 ------------------------GGTTT--------------

1920360 -------------------------------------------

5941215 -------------------------------------------

1265444 -------------------------------------------

205311 -------------------------------------------

539283 -------------------------------------------

2447119 -------------------------------------------

7001362 -------------------------------------------

4714083 -------------------------------------------

5287660 -------------------------------------------

1297993 -------------------------------------------
