# Supplementary material for: Simple and Complex Centromeric Satellites in Drosophila Sibling Species
Source: Genetics. 2018 Jan 5;208(3):977–90. doi: 10.1534/genetics.117.300620 (PMC5844345; doi:10.1534/genetics.117.300620)
Supplement: Supplementary file 10 [file 977FileS6.docx]

**File S6. Alignment of *simcent2* reference sequences.** Colored text and shading mark homologous subregions. 10mers used in counting are underlined. Yellow highlighting in #526886 marks sequence homologous to *simcent1*.

3225452 ------------------------------------------------------------

66273 ------------------------------------------------------------

8388730 ------------------------------------------------------------

526886 ------------------------------------------------------------

3225449 ------------------------------------------------------------

2452220 ------------------------------------------------------------

2063971 ---------AAGGA-CCATTAATTTGCATAAAATTCGCATAAAATGCACTCCAT----GA

3231237 -------------------------------TGAGCGGAGATTTTTACAACACCGGAAAT

2516370 ------------------------------------------------------------

5127241 ------------------------------------------------------------

1828661 ----------TGTTATTATTGATAAATAAAATGAATATCTGGTTATGGAACACATTAAAA

4981552 ------------------------------------------------------------

607087 ------------------------------------------------------------

6085466 ATGTTTACAATGCTGGAACAAATTTAAAAAACAAAACGTTAAGGTATGAACCATTTTGTT

6089682 ------------------------------------------------------------

3225452 ------------------------------------------------------------

66273 ------------------------------------------------------------

8388730 ------------------------------------------------------TTCTCT

526886 ------------------------------------------------------------

3225449 ------------------------------------------------------------

2452220 ------------------------------------------------------------

2063971 ATAAGAAACTGTTGAGCAGACT-----GAGTCGCAGGATTACAGGGATAGCGCGAAAGTC

3231237 GTTACACAACTTTGGACTGGCATATGAATTATA----TTTTAATCATCCAGCCAGATTCT

2516370 ------------------------------------------------------------

5127241 ------------------------------------------------------------

1828661 GTTTAAGCTATTGCGTTGTTTTAATGCAGATTGGTTTAGAGCTACAACATACCATTCTCT

4981552 ------------------------------------------------ATACCATTCTCT

607087 -------------AACTATTTAAATGCAGATTGGTTTAGAGATAGAACATACCATTCCCT

6085466 ATAATTGATAATTAAAATGAAAAATGCAGATTTGTTTAGAGCTAGAACATACCATTCTCT

6089682 ------------------------------------------------------------

3225452 -----------------------------------------------AGCATGTACCAAT

66273 ---------------------AAAACAAATTTAAAACACAATCGTTTAGCATGTACCAAT

8388730 AAGTTTGAGTTTCACAATACTAAAACAAATTTAAAACACAATCGTTTAGCATGTACCAAT

526886 ------------------ACTAAAAAAAATTTAAAACACAATCGTTTAGCATGTACCAAT

3225449 -----------------------------------------------AGCATGTACCAAT

2452220 ---------------------------ACTTTAAAACACAATCGTTTAGCATGCAACAAT

2063971 CCTCATGAGTTTCACAATACTAAAACTACTTTAAAACACAATCGTTTAGCATGCAACAAT

3231237 GTCGGCGTTTTTCACAATACTAAAACTACTTTAAAACACAATCGTTTAGCATGCAACAAT

2516370 ----------------------AAACAAATTTAAAACACAATCGTTTAGCATGCACCAAT

5127241 -----------------------ATCAAGTTTAAAACACAATCGTTTAGCATGCACCAAT

1828661 AGGTGTGAGTTCCACAATACTAAATCAAGTTTAAAACACAATCGTTTAGCATGCACCAAT

4981552 AGGTGTGAGTTCCACAATACTAAATCAAGTTTAAAACACAATCGTTTAGCATGCACCAAT

607087 AGGTTTTAGTTTCACAATACTAAAACAAATTTAAAACACAATCGTTTAGCATGCACCAAT

6085466 AGGTTTGAGTGTCACAATACTAAATCAAGTTTAAAACACAATCGTTTAGCATGCACCAAT

6089682 ATGTTTGAGTTTCACAATACTAAAACTACTTTAAAACACAATCGTTTAGCATGCAACAAT

****** * ****

3225452 TTGCTTTGAGTGATGCACTGCAACGCTGATAATTATGAAAAACGTAACAAAATATAATGC

66273 TTGCTTTGAGTGATGCACTGCAACGCTGATAATTATGAAAAACGTAACAAAATATAATGC

8388730 TTGCTTTGAGTGATGCACTGCAACGCTGATAATTATGAAAAACGTAACAAAATATAATGC

526886 TTGCTTTGAGTGGTGCACTGCAACGCTGATAATTATGAAAAACGTAAAATTGTAATGGTT

3225449 TTGCATTGAGTAATTAACTGCAACGCTGCTAATTTTCCACCAATTTGCTTTGAGTGATGC

2452220 TTGCTTTGAGTGATTAACTGCAACGCTGCTAACTATCCACCAATTTGCTTCGTGTGATGC

2063971 TTGCTTTGAGTGATTAACTGCAACGCTGCTAATTATCCACCAATTTGCTTTGAGTGATGC

3231237 TTGCTTTGAGTGATTAACTGCAACGCTGCTAACTATCCACCAATTTGCTTTGAGTGATGC

2516370 TTGCTTTAAGTGATTAACTGCAACGCTGCTAATTATCCACCAATTTGCTTTAATACGTTA

5127241 TTGCTTTAAGTGATTAACTGCAACGCTGCTAATTATCCACCAATTTGCTTTGAGTGATGC

1828661 TTGCTTTAAGTGATTAACTGCAACGCTGCTAATTATCCACCAATTTGCTTTGAGTGATGC

4981552 TTGCTTTAAGTGATTAACTGCAACGCTGCTAATTATCCACCAATTTGCTTTGAGTGATGC

607087 TTGCTTTAAGTGATTAACTGCAACGCTGCTAATTATCCACCAATTTGCTTTAAGTAATGC

6085466 TTGCTTTAAGTGATTAACTGCAACGCTGATAATTATCCACCAATTTGCTTTGAATGATGT

6089682 TTGCTTTGAGTGATTAACTGCAACGCTGCTAACTATCCACCAATTTGCTTCGTGTGATGC

**** ** *** * ************ *** * * * * *

3225452 TTATGGTAAGCAATTGGTTGTTGCTTTATGCAATTTTTTAGAGCTAGAACA---------

66273 TTATGGTAAGCAATTGGTTGTTGCTTTATGAAATTGAAGATTTGTTTAGTGCTAGAA---

8388730 TTATGGTAAGCAATTGGTTGTTGCTTTATGAAATTGAAGATTTGTTTAGTGCTAGAA---

526886 TTGTTTTAATTTCATGTACCCTCTGTGAGCCAATAAGCTAG-----ATGACCTGCGATGT

3225449 ACTGCAAGCCTGATAATTATGAAGAAAATAACAAAATAGAATCCTTATAATAAGCAATTG

2452220 ACTGCAACGCTGATAATTATGAAAAACGTAACAAAATATAATGCTTATAGTAAGCAATTG

2063971 ACTGCAAAGCTGATAATTATGAAAAGCGT-------------------------------

3231237 GCTGCAACGCTGATAATTATGAAAAACACAACAAAATAGAATGCT---------------

2516370 AGGTATGGACCATTTCT-------------------------------------------

5127241 --------------------------------AAAATAGAATGCTTATGGTAAGCAATTG

1828661 --------------------------------AAAATAGAATGCTTATGGT---------

4981552 --------------------------------AAAATAGAATGCTTATGGTAAGCAATTG

607087 ACTGCAAAGATGATAATTATGAAAAACATAACAAAATTTAATGCTTATAGTAAGCAATTG

6085466 ACTGCAACG---------------------------------------------------

6089682 ACTGCAACGCTGATAATGACGGTTTGATGCCATGGCTGACGGGCAGCCACTTTAAGATTG

3225452 ----TTTGTATTCTCTAGGCTTGATATTTACAATGATGGAACAAATTTAAATAACAAAAC

66273 ----CTAGAATTCTCTAGGCTTGATGTTTACAATGCTGGAACAAATTTAAATAACAAAAC

8388730 ----CTAGAATTCTCTAGGCTTGATGTTTACAATGCTGGAACAA----------------

526886 AGTTTCAGGAGCTGCGCGGAAGTAAAATTAGAACAATGGATGTCGTAAA----TCAAGGA

3225449 GTTGTTGCTTTATGCAATTTTTTATTGCGCCCGATTCTTCTCGAAGCTGACGGCCATACT

2452220 GTTGTTGCTTTATGCAATTTTTTAGAGCTAGAACATTTGGGGCCGTTAT----GCATGCG

2063971 ------------------------------------------------------------

3231237 ------------------------------------------------------------

2516370 ------------------------------------------------------------

5127241 GTTGTTGCTTTATGCTATTTTTTAGAGCTAGAACATTTGTATTCTCTAGGCTTGAT

1828661 ------------------------------------------------------------

4981552 GTTGTTGCTTTATGCT-----------------------------------------

607087 GTTTTTGCTTTATGCAATTGAAG----------------------------------

6085466 ------------------------------------------------------------

6089682 CGCAAAAGTCCCTGGAGGCGTTCTCGGAGCTAATAAAG-----CGACTG----GGCAGCG

3225452 GTTAAGGTATGGACCATTTTATTATAATTGATAACTAAAACGAATATCTGATTATGGAAA

66273 GTTAAGGT----------------------------------------------------

8388730 ------------------------------------------------------------

526886 CCAAAGATT------AAGAGGTTCGTATCTAAATAAT-------TCGTT-----------

3225449 TCCACACTGACAACCACCTTGAGCAGCAAGATCTTGGCCAGCTTTCACTGGGTC------

2452220 CGATGCG--------TGACTGCCACAATGCCCATTTTCCAGCTTTCCGC-----------

2063971 ------------------------------------------------------------

3231237 ------------------------------------------------------------

2516370 ------------------------------------------------------------

5127241 GTTTACAATGCTGGAACAATGCAAAAGCAACTCAAACAAAAGTGTACCCATGAAAATATC

1828661 ------------------------------------------------------------

4981552 ------------------------------------------------------------

607087 ------------------------------------------------------------

6089682 ACTTCAA----------TGCATACAC--G-------------------------------

6085466 ------------------------------------------------------------

3225452 ACATTAAAAG

66273 ----------

8388730 ----------

526886 ----------

3225449 GCC-------

2452220 ----------

2063971 ----------

3231237 ----------

2516370 ----------

5127241 GAATGGGT--

1828661 ----------

4981552 ----------

607087 ----------

6085466 ----------

6089682 ----------
